# A twin delayed deep deterministic-based control method for a full vehicle semi-active suspension system

**DOI:** 10.1371/journal.pone.0359102

**Published:** 2026-09-25

**Authors:** Yongjun Wan, Xiaoming Wang, Gang Zhi, Lei Yang, Juye Wang, Xiangcheng Zhang, Yan Zhang

**Affiliations:** 1 Henan College of Transportation, Zhengzhou, China; 2 China Construction Fifth Bureau (HENAN) Construction Co., Ltd., Zhengzhou, China; 3 School of Civil Engineering, Zhengzhou University, Zhengzhou, China; National University of Singapore, SINGAPORE

## Abstract

To improve the convergence issues of deep reinforcement learning in the control of vehicle semi-active suspension, and enhance the control effectiveness of the vehicle’s semi-active suspension, a Twin Delayed Deep Deterministic-based control framework tailored for the high-dimensional nonlinear dynamics of the vehicle semi-active suspension model is proposed. Rather than merely applying standard deep reinforcement learning, this study introduces a domain-specific state-action mapping mechanism and a multi-objective reward function to physically constrain the neural network outputs and coordinate the full-vehicle dynamic responses. The simulation program for 7-degree-of-freedom vehicle semi-active suspension model embedded in the proposed control method is developed using MATLAB software. The validity of the proposed control method is verified using the developed program under different road roughness and speeds. The results indicate that compared to the passive suspension the classical linear Skyhook control method and the Fuzzy control method the proposed Twin Delayed Deep Deterministic based method achieves superior control effectiveness. Specifically in terms of the vehicle body vertical acceleration the proposed method achieves a reduction of 49.41% compared to the passive suspension and 9.33% compared to the Skyhook method on a Class A road at 60 km/h a reduction of 24.82% compared to the passive suspension on a Class A road at 120 km/h and a reduction of 3.19% compared to the Skyhook method on a Class B road at 60 km/h. Furthermore compared to the traditional Fuzzy control method the proposed method demonstrates superior vibration suppression effectiveness across all evaluated operating conditions achieving significantly lower frequency weighted vertical root mean square accelerations. Furthermore the power spectral density analysis demonstrates that the proposed method significantly suppresses the low frequency resonance of the sprung mass across varying road conditions compared to the passive Skyhook and Fuzzy methods, further validating its superior broadband vibration isolation capability. This demonstrates that the Twin Delayed Deep Deterministic-based control method for the vehicle semi-active suspension model has the advantages of fast convergence speed and good control effectiveness under road surface excitation.

## 1. Introduction

During vehicle driving, the motion relationship between the vehicle and the road surface usually includes three directions: transverse, longitudinal, and vertical. When affected by changes in the road surface, the vehicle body moves up and down. When driving or riding in a vehicle, people expect to feel the effects of as little vertical excitation as possible while not affecting driving handling. Therefore, scholars typically focus on the vehicle’s response to road surface excitation when discussing vertical dynamics. The vehicle suspension supports the body during driving, isolates road surface excitation, and provides driving comfort. Using the suspension to fix the body to the axle, the body can be isolated from the axle by the springs and dampers of the suspension, thereby reducing the impact of the body on the roughness of the road. Generally, the suspension design problem can be simplified to the coupling problem of spring and damper in the vehicle suspension design process [1]. For example, in a typical quarter suspension model, suspension design problems can be reduced to a 3-DOF system involving pavement, sprung mass, and unsprung mass [2].

In terms of controllability, vehicle suspensions are generally classified into passive suspension, semi-active suspension, and active suspension. Semi-active suspension is widely used in new passenger cars because of its lower cost and better control effect. In recent years, there also have been some developments in its design [3] and operational principles [4]. Studies have shown that the performance of semi-active dampers can closely approach that of fully active systems in balancing ride comfort and road safety [5]. To further enhance control effectiveness, advanced force-tracking approaches integrating semi-active dampers with semi-active inerters have been proposed [6]. Among the various damping devices developed, the magnetorheological fluid damper (MFD) is one of the most widely used in recent years [7], and there has been considerable research on the design methods [8] and mechanical models [9] of such dampers. The MFD, which has been successfully designed and validated even for full-scale railway vehicle suspensions to improve ride quality [10], changes the dynamic viscosity of the magnetorheological fluid in the damper at different currents, thus producing different damping forces [11], and enables various advanced control adaptations [12]. MFDs have the advantages of fast response, mature technology, and a large damping adjustment range. The response speed of magnetorheological fluids under changes in magnetic fields can reach the millisecond level [13], which is one of the reasons for the fast response speed of MFDs [14]. Not only in passenger vehicle suspension control, but MFDs are also widely applied in other engineering domains. For instance, in building structures, MR dampers are utilized to mitigate earthquake responses [15], and combined with neural network-based intelligent control strategies to resolve time-delay issues [16]. Furthermore, for high-speed rail vehicles, semi-active suspensions equipped with MR dampers and predictive model controllers have been shown to significantly improve ride quality while maintaining running safety [17]. These research efforts provide significant inspiration for the study presented in this paper on the semi-active control of vehicle suspension.

The vehicle suspension model serves to validate the damping effects of the vehicle’s suspension system. A model typically yields simulation results close to those observed in the suspension’s actual operation [18]. For individual axle-based suspension studies, employing a quarter suspension model proves effective [19]. However, due to considerations of the realism of vehicle suspension, such as excitation characteristics between front and rear wheels, suspension layout, and collecting vehicle angle information, utilizing half or full-vehicle models becomes necessary [20]. Furthermore recent studies emphasize the critical importance of integrated chassis control in vehicle models to meet modern performance demands. For example to simultaneously enhance longitudinal dynamics and vertical ride comfort advanced frameworks based on multi-agent systems have been proposed to integrate active suspension systems with torque vectoring [21]. Similarly model predictive controllers have been developed for such integrated systems to optimize energy saving and safety under linear models [22]. Driven by the necessity of such comprehensive dynamics modeling and multi-axis coordination this paper employs a seven-degree-of-freedom vehicle suspension model, which is a full vehicle model, to thoroughly investigate suspension control effects.

In research on semi-active suspension control strategies, traditional control methods such as the skyhook control method serve as baseline comparisons due to their simple and practical control algorithms, good control effects, and lower development difficulty [23]. In particular, the linear sky-hook control method, when applied to a semi-active suspension system with MFD, significantly enhances suspension performance by employing linear functions to control the current of dampers. However, the skyhook control method typically applies only to quarter suspension models, often lacking optimal solutions for entire vehicle control. Linear Quadratic Regulator (LQR) controllers are also frequently used in semi-active suspension control. Yet, due to challenges in determining weighted matrices for semi-active suspensions [24], and optimizing the control parameters under complex constraints [25], the development of these controllers poses significant difficulties. Addressing these issues, employing machine-learning and data-driven control methods significantly reduces the complexity of control strategy development. For instance, to tackle the severe nonlinearity and rate-dependent hysteresis of magneto-rheological devices without relying on complex analytical models, data-driven sliding mode control approaches have been successfully implemented [26]. Such intelligent methods show promising control effects even on entire vehicle models [27]. However, particularly for advanced machine learning branches like deep reinforcement learning (DRL) algorithms, the control effects often rely heavily on the gradient direction of convergence. Therefore, establishing models scientifically remains a key focus in these data-driven methods.

Deep reinforcement learning is a machine learning decision-making method that has attracted more attention in recent years [28]. Usually, a reinforcement learning (RL) algorithm uses a value function to judge the value of behavior [29], which is typically modeled within the framework of a Markov chain [30]. By using a neural network model to establish a value function, the established DRL algorithm can more easily achieve gradient descent. Unlike other machine learning methods, DRL, which relies on the learning process of actions based on environmental variables, is widely applied in fields such as production and daily life, where machine autonomy is crucial. Examples like AlphaGo have garnered significant attention among scholars [31]. In recent years, there have been attempts to apply DRL to production processes [32].

Within the research on DRL strategies, two commonly used structures are the value Q-function method [33] and the Actor-critic method [34]. The DQN algorithm utilizes a Q-value network to obtain Q-values for several actions and selects the best action, commonly applied to discrete action spaces. On the other hand, the DDPG algorithm employs an actor network and a critic network. It optimizes the critic network by evaluating the values of actions in the environment and then optimizes the actor network based on the critic network’s evaluations. This method is suitable for continuous action spaces. DDPG-based algorithms have demonstrated robust decision-making capabilities in complex vehicle dynamics, such as human-machine shared steering control during emergency obstacle avoidance [35]. However, DRL algorithms often face challenges regarding sample efficiency and Q-value estimation errors. To address this, recent studies have developed backtrack temporal difference learning methods to enhance target Q-value estimation precision [36]. Building on the need to improve convergence, in 2018, Fujimoto et al. introduced the Twin Delayed Deep Deterministic (TD3) algorithm [37], which uses two critic networks to enhance the convergence process. TD3 improves upon the DDPG algorithm, enhancing the convergence of the Actor-Critic method.

In recent years, driven by the rapid trends of vehicle electrification [38] and intelligent autonomous driving [39], active and semi-active suspension technologies have experienced unprecedented development to meet increasingly stringent demands for ride comfort and integrated chassis control. While the performance of traditional controllers like Skyhook is strongly influenced by hardware constraints, such as the response time of magnetorheological valves [40], DRL control algorithms using the Actor-Critic structure (such as DDPG) can often achieve more robust and adaptable control. For instance, recent studies have successfully deployed Soft Actor-Critic (SAC) models in real vehicles, enabling the suspension system to dynamically adapt to varying real-road disturbance profiles [41]. In the realm of semi-active suspensions, scholars have extensively validated the efficacy of the Actor-Critic method (such as the DDPG algorithm) in controlling the vehicle’s quarter suspension model [27]. Additionally, there is research exploring the combination of DRL methods with traditional control approaches to achieve control in a relatively stable manner [42]. However, there has been a slight scarcity of research regarding DRL in the context of full-vehicle suspension models. Through testing, the author suggests this might be related to the convergence issues of DDPG, as at times, using the DDPG algorithm can make it difficult for Q-value predictions to converge to a consistently stable level.

To address the issues of slow convergence speed and relatively low convergence in the control of semi-active suspension with the DDPG algorithm under the full vehicle suspension model, and to enhance the effectiveness of the controller, this paper first introduces a 7-DOF semi-active suspension model for vehicles and a road surface model established using the PSD specified by ISO 8608. Specifically, the fundamental improvements of the proposed TD3 framework over conventional DRL methods lie in two aspects. First, unlike the DDPG algorithm which suffers from severe Q-value overestimation in the highly coupled 7-DOF full-vehicle environment, the integrated TD3 architecture employs twin delayed critics to ensure stable policy evaluation under complex multi-axis road excitations. Second, a customized state-action mapping and a physically meaningful reward function are designed to bridge the gap between unconstrained DRL algorithms and the strict physical boundaries of magnetorheological fluid dampers (MFDs), ensuring that the generated control currents are both theoretically optimal and physically executable. Based on the TD3 algorithm, a TD3-based control method is proposed for the current of the semi-active control device in the vehicle suspension. This is achieved through the redefinition and validation of the neural network model, training parameters, and reward functions in DRL. In the control method, the state is updated based on the 7-DOF semi-active suspension model for vehicles. Using MATLAB software, this paper constructs a 7-DOF semi-active suspension model for vehicles, develops a program of the TD3-based control method for vehicle suspension, and validates the proposed controller under varying road roughness and speeds. The results of the semi-active suspension controlled by the TD3-based control method are compared with those of passive suspension, the classical linear Skyhook control method, and the traditional Fuzzy control method. The effectiveness and convergence of the TD3-based control method are validated through this comparison. Furthermore recent studies emphasize the importance of advanced integrated chassis control frameworks. For instance sophisticated methodologies like fuzzy game theoretic tube model predictive control have been developed to ensure robust constraint satisfaction and coordinate heterogeneous actuators for integrated vehicle lateral stability [43]. Inspired by the application of fuzzy logic in advanced vehicle control and to thoroughly validate the proposed TD3 algorithm this paper employs a sophisticated Fuzzy Logic Control algorithm as an advanced benchmark alongside the traditional Skyhook method.

The remainder of this paper is organized as follows. Section 2 establishes the 7-DOF vehicle semi-active suspension model and derives its dynamic equations. Section 3 introduces the road surface roughness model based on the ISO 8608 standard. Section 4 details the design of the TD3-based semi-active suspension controller, including the definitions of the state space, action space, and reward function. Section 5 presents the numerical simulation results and discussions, evaluating the proposed control method against baseline methods under different road classifications and vehicle speeds. Finally, the conclusions are drawn in Section 6.

## 2. 7-DOF vehicle semi-active suspension model

**Fig 1:**
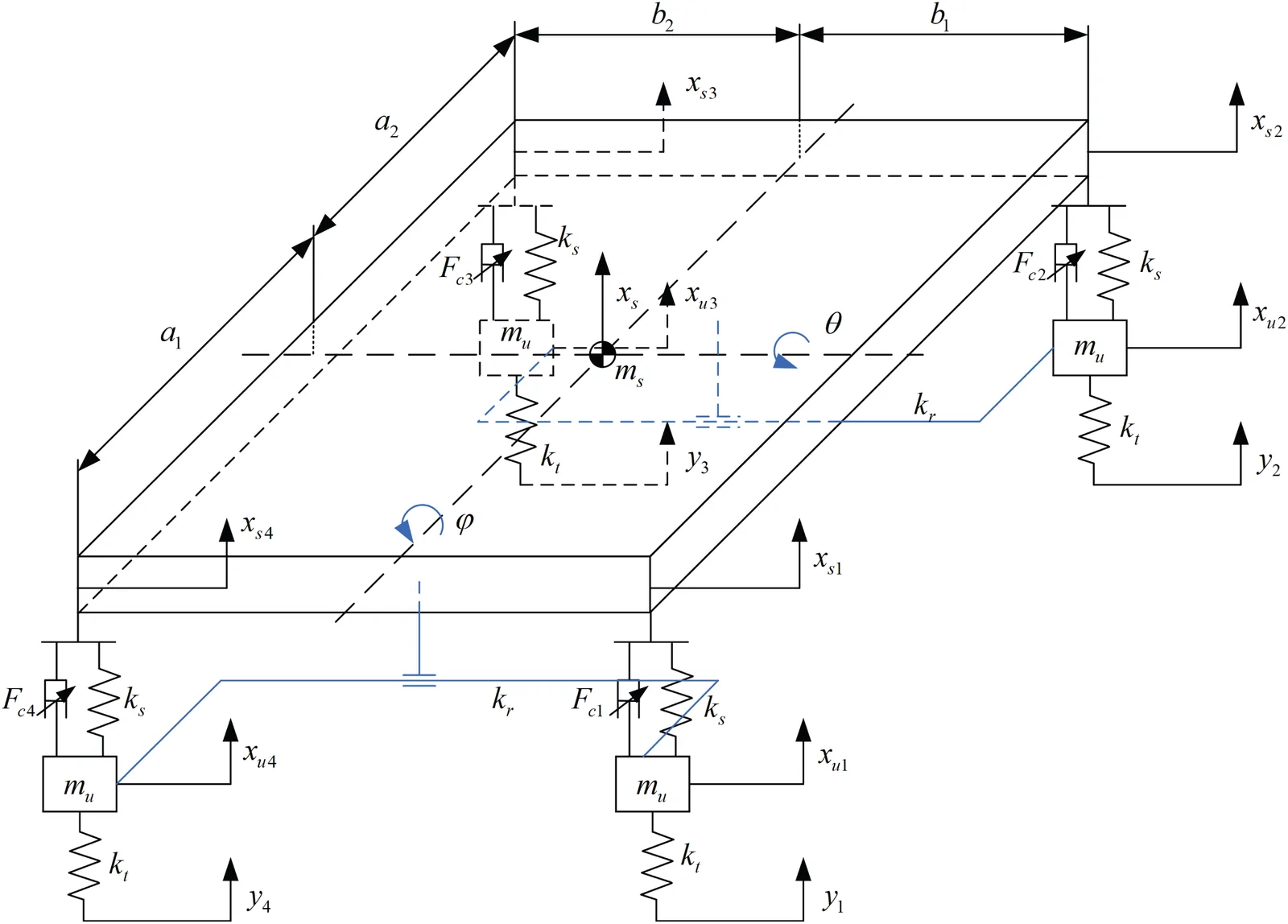
7-DOF semi-active vehicle suspension model.

The configuration of the 7-DOF vehicle semi-active suspension model adopted in this study is shown in Fig 1 [44]. It can be seen that the suspension consists of springs, adjustable dampers, and anti-roll bars, springs and adjustable dampers are installed in the suspension part between the spring-loaded (i.e., sprung) and non-spring-loaded (i.e., unsprung) masses, while two anti-roll bars are installed between the left and right unsprung masses as a vehicle roll stabilization mechanism.

In Fig 1, the center of mass of the sprung mass has three degrees of freedom: vertical displacement $x_{s}$, lateral roll angular displacement φ, and longitudinal pitch angular displacement θ, while each of the four unsprung masses has one degree of freedom, i.e., the vertical displacement of the ith (i = 1, 2, 3, 4) unsprung mass $x_{ui}$. $m_{s}$ is the sprung mass, $k_{s}$ is the spring stiffness of the suspension, $F_{ci}$ is the damping force of the ith damper, $m_{u}$ is the unsprung mass, $a_{\text{1}}$, $a_{\text{2}}$, $b_{\text{1}}$, $b_{\text{2}}$ determine the position of the sprung mass center of gravity, $y_{i}$ represents the road surface excitation experienced of the ith tire. $k_{r}$ is the stiffness of the anti-roll bar, $k_{t}$ is the equivalent spring stiffness of the tire.

When the corners of the vehicle φ and θ are considered small enough, the arc length of roll angular displacement and pitch angular displacement at the suspension hinge can be used instead of the chord length, the vertical displacement of the ith hinged position of the vehicle’s four suspensions $x_{si}$ is:


$$\begin{array}{c} \left\{ \begin{array}{c} @c@x_{s1}=x_{s}+b_{\text{1}}\varphi -a_{\text{1}}\theta \\ x_{s2}=x_{s}+b_{\text{1}}\varphi +a_{\text{2}}\theta \\ x_{s3}=x_{s}-b_{\text{2}}\varphi +a_{\text{2}}\theta \\ x_{s4}=x_{s}-b_{\text{2}}\varphi -a_{\text{1}}\theta \end{array}\right. \end{array}$$ (2.1)

Suspension spring force $F_{si}$ can be expressed as


$$\begin{array}{c} F_{si}=k_{s}(x_{si}-xui) \end{array}$$ (2.2)

The equivalent spring force of the tire $F_{ti}$ can be calculated by


$$\begin{array}{c} F_{ti}=\left\{ \begin{array}{c} @c@0,\begin{array}{cc} & y_{i}<x_{ui} \end{array}\\ k_{t}\left(x_{ui}-y_{i}\right),\begin{array}{cc} & y_{i}\geq x_{ui} \end{array} \end{array}\right. \end{array}$$ (2.3)

The torsion of the rear and front anti-roll bar $M_{rf}$, was expressed as


$$\begin{array}{c} \left\{ \begin{array}{c} @l@M_{rf}=k_{r}\left[\varphi -arcsin\left(\frac{x_{u1}-x_{u4}}{b_{\text{1}}+b_{\text{2}}}\right)\right]\\ M_{rr}=k_{r}\left[\varphi -arcsin\left(\frac{x_{u2}-x_{u3}}{b_{\text{1}}+b_{\text{2}}}\right)\right] \end{array}\right. \end{array}$$ (2.4)

The 7-DOF vehicle suspension model uses an MFD model. The damping force of the ith suspension $F_{ci}$ is obtained by the formula:


$$\begin{array}{c} F_{ci}=f_{d}\left(I_{i},x_{si}-x_{ui},{\dot{x}}_{si}-{\dot{x}}_{ui}\right) \end{array}$$ (2.5)

where $f_{d}$ represents the function of the damping force model for MFD, $I_{i}$ is the control current of the MFD in the ith suspension, and the dots above variables in Eq. (2.5) denote their derivatives concerning time.


$$\begin{array}{c} \dot{x}=\frac{\partial x}{\partial t} \end{array}$$ (2.6)

After obtaining the force parameters in the suspension, the accelerations of each component of the vehicle can be obtained by using Newton’s second law:


$$\begin{array}{c} {\ddot{x}}_{s}=\frac{\text{1}}{m_{s}}\sum_{i=1}^{\text{4}}\left(F_{ti}-F_{ci}-F_{si}\right)-g \end{array}$$ (2.7)

$$\begin{array}{c} \ddot{\varphi }=\frac{\text{1}}{I_{y}}\left[b_{\text{1}}\left(F_{t1}-F_{c1}-F_{s1}+F_{t2}-F_{c2}-F_{s2}\right)-b_{\text{2}}\left(F_{t3}-F_{c3}-F_{s3}+F_{t4}-F_{c4}-F_{s4}\right)-\frac{M_{rf}+M_{rr}}{b_{\text{1}}+b_{\text{2}}}\right] \end{array}$$ (2.8)

$$\begin{array}{c} \ddot{\theta }=\frac{\text{1}}{I_{z}}\left[a_{\text{2}}\left(F_{t2}-F_{c2}-F_{s2}+F_{t3}-F_{c3}-F_{s3}\right)-a_{\text{1}}\left(F_{t1}-F_{c1}-F_{s1}+F_{t4}-F_{c4}-F_{s4}\right)\right] \end{array}$$ (2.9)

$$\begin{array}{c} \left\{ \begin{array}{c} @l@{\ddot{x}}_{u1}=\frac{\text{1}}{m_{u1}}\left(F_{t1}-F_{s1}-F_{c1}+\frac{M_{rf}}{b_{\text{1}}+b_{\text{2}}}-m_{u1}g\right)\\ {\ddot{x}}_{u2}=\frac{\text{1}}{m_{u2}}\left(F_{t2}-F_{s2}-F_{c2}+\frac{M_{rr}}{b_{\text{1}}+b_{\text{2}}}-m_{u2}g\right)\\ {\ddot{x}}_{u3}=\frac{\text{1}}{m_{u3}}\left(F_{t3}-F_{s3}-F_{c3}-\frac{M_{rr}}{b_{\text{1}}+b_{\text{2}}}-m_{u3}g\right)\\ {\ddot{x}}_{u4}=\frac{\text{1}}{m_{u4}}\left(F_{t3}-F_{s4}-F_{c1}-\frac{M_{rf}}{b_{\text{1}}+b_{\text{2}}}-m_{u4}g\right) \end{array}\right. \end{array}$$ (2.10)

where g is the acceleration of gravity, $I_{y}$ and $I_{z}$ are the moments of inertia of the sprung mass in the φ and θ directions, respectively.

In the 7-DOF vehicle suspension model, each step uses the velocity and displacement of each part of the vehicle to calculate the force of suspension and get the acceleration of each part, updating the state of the suspension.

## 3. Road surface roughness model

In vehicle motion, the road surface typically determines the working condition of the suspension system. Therefore, setting an appropriate road surface roughness can make the simulation closely match the actual vehicle motion to a greater extent. In this simulation, it is stipulated that the vehicle is traveling on a straight road with vertical displacement $y_{i}$ in the vertical direction of the road. The input of road excitation can be considered as two separate channels, one for the left and one for the right, each independently meeting the standard for road surface excitation. At the same time, the road surface excitation input between the front and rear wheels has a time delay of length based on the vehicle’s speed.

In the normal operation of passenger vehicles, the road surface roughness typically complies with the road standards of the local country or region. In this paper, ISO 8608 is chosen as a reference to establish a road surface roughness model [45], the power spectral density (PSD) of vertical displacement is used to represent the road surface roughness model.

PSD is a commonly used method for testing road surface roughness, specifically described as the power of vertical displacement in angular spatial frequency $G_{d}\left(\Omega \right)$ (m3) under the angle spatial frequency Ω (rad/m). Referring to the description of ISO 8608 by Mucka in 2018 [46], the relationship between Ω and $G_{d}\left(\Omega \right)$ is established by the following formula:


$$\begin{array}{c} G_{d}\left(\Omega \right)=G_{d}\left(\Omega_{\text{0}}\right){\left(\Omega /\Omega_{\text{0}}\right)}^{-w} \end{array}$$ (3.1)

The function uses an angular spatial reference frequency $\Omega_{\text{0}}$ and fit exponent w to define a straight line in the logarithmic space of PSD. In ISO 8608, Table 1 illustrates the limits and geometric mean values of $G_{d}\left(\Omega_{\text{0}}\right)$ under different road surface standards (w=2, $\Omega_{\text{0}}=1$ rad/m).

**Table 1:** Road classification.

Road class	Angular spatial frequency units, $G_{d}\left(\Omega_{0}\right)$(10^−6^ m^3^)	
	limit	Geometric mean
A	<2	1
B	2-8	4
C	8-32	16
D	32-128	64
E	128-512	256

*Note*: The fit exponent w=2 is assumed.

Typical displacement settings are verified by selecting vertical displacements at specific PSD center frequencies to assess whether the road surface meets the smoothness standards. When establishing the road surface roughness model in this article, first take a certain length of road surface, superimpose excitation signals within all sampled frequency ranges, and then use the PSD method to validate the generated road surface signal. The signal output by the road surface excitation model is stored as a vector $Y={\left[\begin{array}{cccc} @cccc@y_{\text{1}} & y_{\text{2}} & y_{\text{3}} & y_{\text{4}} \end{array}\right]}^{T}$ that changes over time at the model input interface.

## 4. TD3 semi-active suspension controller

When dealing with the problem of semi-active suspension control in vehicles, one often encounters very severe nonlinearities. This issue arises due to the nonlinearity of the damper control and the nonlinearity of suspension response when going from the semi-active suspension state to the control signal in the reverse direction. When using the linear skyhook control method, the suspension model propagates forward through the state space, and the control signal is generated using linear interpolation, thereby achieving closed-loop control. Similarly, using DRL methods can bypass the study of nonlinear problems and find a control law for the suspension that is better suited to the specified suspension compared to traditional control methods through training models.

### 4.1. TD3 algorithm

DRL is a machine decision-making method used in RL, which employs neural network techniques to build functions for gradient descent and solve complex problems. In DRL, an agent is typically used as the entity making decisions. The agent interacts with the environment, updates its strategy, and aims to obtain higher expected rewards. In classical RL scenarios, the agent takes actions in the environment, receives rewards, and updates its state, forming a decision process based on Markov chains (Markov Decision Process). A standard Markov chain can be represented as $\left(s,a,r,s^{\prime },a^{\prime },r^{\prime },...\right)$, where s,a,r are the current time-step state, action, and reward; $s^{\prime },a^{\prime },r^{\prime }$ are the next time-step state, action, and reward. The sum of expected subsequent rewards after an action is taken is defined as the value, denoted as $y=r+r^{\prime }+...$. In DRL, the decision-making process in the Markov chain is achieved by establishing a neural network.

For suspension control, the TD3 control method has lower generalization ability than skyhook damping control but higher than fuzzy PID control. For different suspensions of vehicles, because road excitations generally remain within the range specified by ISO8608, the variations in suspension output, such as suspension velocity and acceleration, are limited across different vehicles. Therefore, by simply adjusting the reward function, a reasonable TD3 controller can be obtained, enabling the transfer of the controller from one suspension set to another. At the same time, the generalization ability of the TD3 controller results has been preliminarily verified: when driving speed and road grade change, the same training results can produce similar outcomes, and the output damping force remains consistently within the normal range without showing prolonged periods of excessively high or low damping. Thus, it can be considered that using the TD3 algorithm to design a controller in a suspension model does not lead to significant overfitting under reasonable parameters. Through multiple training sessions, it is considered that under the same speed condition, approximately 30,000 seconds of training is sufficient to obtain relatively reasonable controller results. For suspension variations occurring over time due to usage, retraining can be performed to obtain more reasonable controller parameters, thereby ensuring the reliability of passenger cars throughout their entire operational cycle.

The Actor-Critic method is a typical RL approach effective for continuous action spaces. In this framework, the agent typically comprises two networks: an actor network and a critic network. The actor network selects actions, and the critic network estimates the expected rewards of those actions. Using deep learning, constructing the actor network and critic network with neural networks can provide reasonable outputs for continuous action spaces. The TD3 algorithm is employed in this article to implement the process of DRL. TD3 algorithm utilizes two Q-networks, making it more resistant to overfitting of the value network. Additionally, TD3 supports delayed updates of target networks, which helps the system compute gradients more accurately for better convergence.

**Fig 2:**
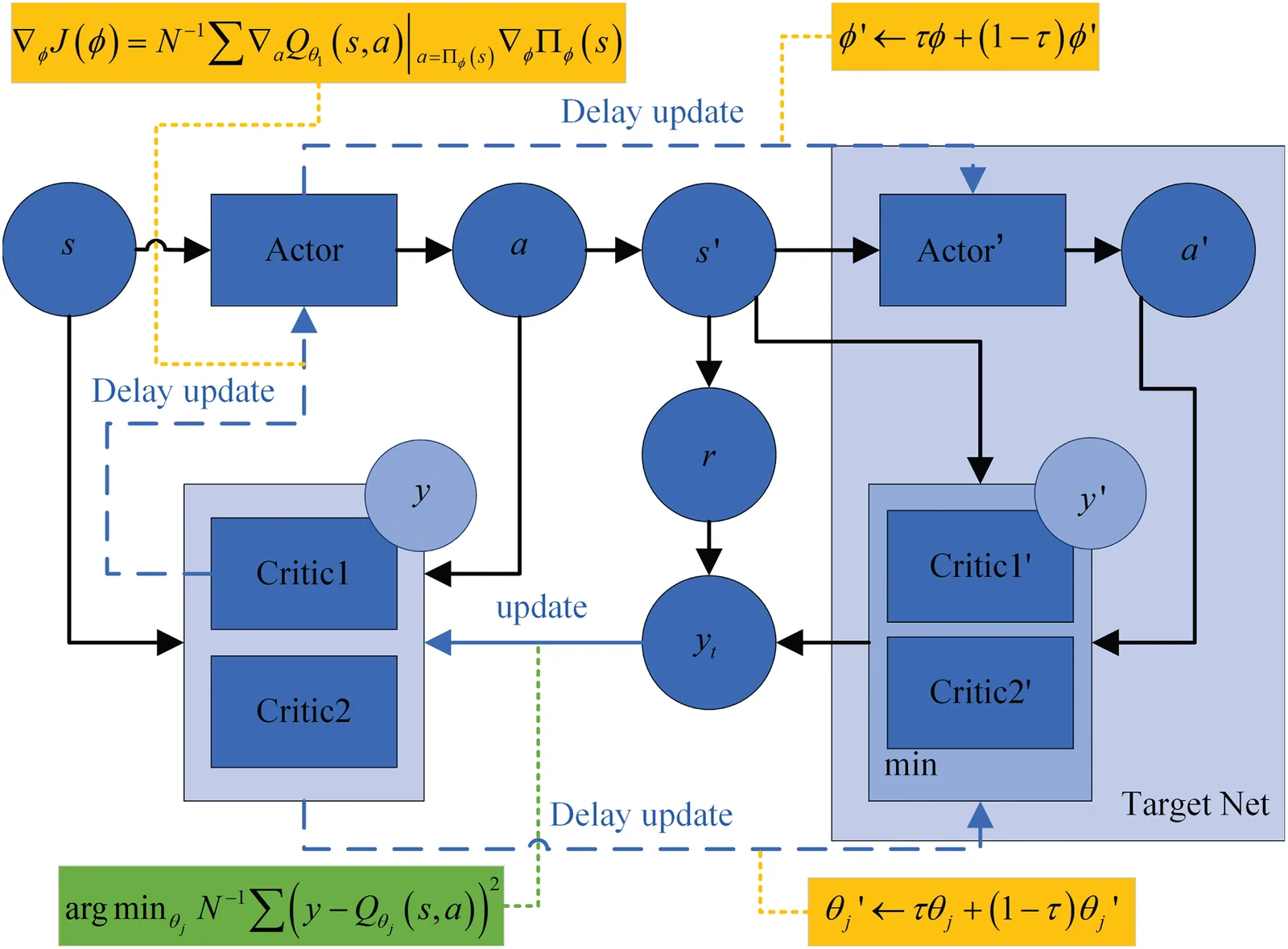
TD3 algorithm diagram.

The workings of the TD3 algorithm’s networks during training can be depicted in Fig 2, The figure illustrates the tasks performed by the actor network and critic network in a loop. The parameters $a^{\prime }$ and $y^{\prime }$ generated by the Target Net section in the figure will not participate in the next iteration; they only contribute to updating the network in the current iteration. The TD3 algorithm features one actor network and two critic networks (Critic1, Critic2). Each network has a corresponding target network for reference, and the target network undergoes delayed updates synchronized with the actor network at fixed intervals. Table 2 displays the correspondence between the network names in the diagram and the function names in this paper.

**Table 2:** Network name – function correspondence table.

Current networks		Target networks	
Name	Function	Name	Function
Actor	$\Pi_{\phi }\left(s\right)$	Actor’	$\Pi_{\phi^{\prime }}\left(s^{\prime }\right)$
Critic1	$Q_{\theta_{\text{1}}}\left(s,a\right)$	Critic1’	$Q_{\theta_{\text{1}}^{\prime }}\left(s^{\prime },a^{\prime }\right)$
Critic2	$Q_{\theta_{\text{2}}}\left(s,a\right)$	Critic2’	$Q_{\theta_{\text{2}}^{\prime }}\left(s^{\prime },a^{\prime }\right)$

In DRL, the reward function can be defined by taking the environmental state s as input and outputting a reward r, thus evaluating the value of an action after it is taken. The reward can be determined by the reward function:


$$\begin{array}{c} r=R\left(s\right) \end{array}$$ (4.1)

where R represents the reward function. In the TD3 algorithm, the action a is computed by the actor network:


$$\begin{array}{c} a=\Pi_{\phi }\left(s\right) \end{array}$$ (4.2)

where $\Pi_{\phi }$ represents the actor network under the parameters parameterized by ϕ. In the TD3 algorithm, s can be represented as a state vector of the continuous action spaces. A single set of network parameters can represent the entire network. Therefore, in this paper, the parameters used for the target actor network $\Pi_{\phi^{\prime }}\left(s^{\prime }\right)$ are denoted as $\phi^{\prime }$. Furthermore, following Q-learning, the value y of the action computed by the critic network in different states can be represented as:


$$\begin{array}{c} y=Q_{\theta_{j}}\left(s,a\right) \end{array}$$ (4.3)

In the equation, y represents the value of an action, which is the sum of rewards for the state vector s and action a. $Q_{\theta_{j}}$ represents the jth critic network under the parameters parameterized by $\theta_{j}$ (j=1,2). In the TD3 algorithm, the parameters used for the target critic networks $Q_{\theta_{j}^{\prime }}\left(s^{\prime },a^{\prime }\right)$ are denoted as $\theta_{j}^{\prime }$.

In the computation, the critic network is passed through the following equation:


$$\begin{array}{c} Q_{\theta_{j}}\left(s,a\right)=r+\gamma 𝔼\left[Q_{\theta_{j}}(s^{\prime },a^{\prime })\right]-\delta \left(s,a\right) \end{array}$$ (4.4)

where γ represents a weighting coefficient, while δ(s,a) denotes the introduced error, and 𝔼 represents the expectation. This formula illustrates the way the critic network operates, indicating that when the future actions cannot be determined, the value of the current action can be expressed as the sum of the expected values of future actions and the current action’s reward, subtracted by an error. Therefore, the value output by the critic network is predictably within a certain range. In the TD3 algorithm used in this paper, Equation 14 can be expressed as $y_{t}=r+\gamma minQ_{\theta_{j}^{\prime }}\left(s^{\prime },a^{\prime }\right)$, representing the value of the action a transmitted by the target critic network for updating the current critic network.

During training, in each iteration, the current state, influenced by the actions of the current actor network, transitions to the next state and receives a reward. The action obtained from the next state, which serves as input, is used to obtain the value $y^{\prime }$ from the target critic network, which is then used to update the current critic network.


$$\begin{array}{c} \theta_{j}={argmin}_{\theta_{j}}N^{-1}\sum {\left(y-Q_{\theta_{j}}\left(s,a\right)\right)}^{\text{2}} \end{array}$$ (4.5)

where argmin denotes the argument that minimizes the subsequent expression, N is the number of samples. In this looping process, the critic network receives gradients based on the target critic network and the rewards obtained from the current actions. At periodic intervals of updates, the gradients from the critic network are used to update the actor network by the deterministic policy gradient network:


$$\begin{array}{c} \nabla_{\phi }J\left(\phi \right)=N^{-1}\sum \nabla_{a}Q_{\theta_{\text{1}}}\left(s,a\right)|a=\Pi_{\phi }\left(s\right)\nabla_{\phi }\Pi_{\phi }\left(s\right) \end{array}$$ (4.6)

where ∇ represents the gradient, and J(ϕ) is the expected return. The current network is employed to update the target network:


$$\begin{array}{c} \left\{ \begin{array}{c} @l@\theta \prime_{j}\leftarrow \tau \theta_{j}+\left(1-\tau \right)\theta \prime_{j}\\ \phi^{\prime }\leftarrow \tau \phi +\left(1-\tau \right)\phi^{\prime } \end{array}\right. \end{array}$$ (4.7)

where τ represents the weighting coefficient used to determine the extent of parameter updates for the Target Networks.

### 4.2. Determining vehicle parameters in DRL

In the scenario of the 7-DOF suspension model of a semi-active vehicle suspension system used in this paper, the TD3-based control method receives a state vector provided by the vehicle suspension. The vehicle model possesses seven degrees of freedom, which are represented using vectors.


$$\begin{array}{c} D=\left[\begin{array}{ccccccc} @ccccccc@x_{s} & \varphi & \theta & x_{u1} & x_{u2} & x_{u3} & x_{u4} \end{array}\right] \end{array}$$ (4.8)

The state vector received by the DRL network is a combined vector of displacements, velocities, and accelerations of the degrees of freedom provided by the vehicle model.


$$\begin{array}{c} s={\left[\begin{array}{ccc} @ccc@D & \dot{D} & \ddot{D} \end{array}\right]}^{T} \end{array}$$ (4.9)

The actions that the actor network should output are the raw values eventually mapped to the control current space of the MFD.


$$\begin{array}{c} a={\left[\begin{array}{cccc} @cccc@\alpha_{\text{1}} & \alpha_{\text{2}} & \alpha_{\text{3}} & \alpha_{\text{4}} \end{array}\right]}^{T} \end{array}$$ (4.10)

In this paper, each real number in the action space vector of the TD3 algorithm is a value that has been processed through the arctangent function in the TD3 actor network, with a characteristic parameter $a_{i}\in \left(-1,1\right)$. In practical control, each original value in the action space output is mapped to the control current vector through a linear function. In conventional DRL applications, unbounded action outputs often lead to actuator saturation or sudden jolts in physical systems. To fundamentally address this, each real number in the action space vector of the proposed TD3 algorithm is processed through the arctangent function. This specific design acts as a smooth, continuous constraint mechanism, intrinsically mapping the neural network’s exploratory outputs into the strict physical boundary of the MFD’s maximum and minimum current limits, thereby preventing high-frequency chattering in the semi-active dampers. Therefore, the process of obtaining the output current through the actor network and mapping can be represented by the following function:


$$\begin{array}{c} I_{\text{target}}=I_{min}+0.5\cdot \left(\Pi_{\phi }\left(s\right)+[\begin{array}{cccc} 1 & 1 & 1 & 1 \end{array}{]}^{T}\right)\cdot \left(I_{max}-I_{min}\right) \end{array}$$ (4.11)

where $I_{max}$ and $I_{min}$ represent the maximum current column vector and the minimum current column vector of the MFD, determined by the effective range of the control current in the MFD. $I_{\text{target}}$ represents the current output column vector.

This specific mapping mechanism explicitly addresses the actuator limitations of the semi-active suspension system. By structurally encoding the physical current boundaries into the neural network’s output via the arctan function, the policy is fundamentally prevented from learning physically un-executable commands. Furthermore, combined with the discrete simulation time step of 0.01 s which implicitly incorporates the physical response delay of the MFD, the reinforcement learning framework ensures that the obtained control strategy strictly respects both the amplitude and temporal limitations of practical actuators.

The reward function R(s) serves as an evaluation criterion for the state of the vehicle suspension, taking in the vehicle state and outputting a scalar value to represent a score. The reward function has been tested and employs a weighted sum of the vertical acceleration and vertical velocity at the transformed vehicle center of mass, achieving relatively effective control performance: in the vehicle suspension model, angular velocity, angular acceleration, vertical velocity, acceleration, and vibration frequency at the vehicle center of mass are key parameters for damping, while our primary concern is vertical acceleration and frequency. Extensive testing shows that using angular velocity/angular acceleration causes the TD3 control method to favor obtaining more rewards for angular velocity/angular acceleration, and setting a reward cap provides little benefit. When vertical acceleration alone is used as the reward function input, the control algorithm tends to prioritize higher rewards at the expense of controlling the suspension’s deformation direction, resulting in uncontrollable vertical displacement changes under control. Therefore, introducing velocity weighting not only keeps the suspension’s motion range within a controllable scope but, as tests indicate, a reasonable velocity weight can adjust the suspension’s vibration frequency to a range less sensitive to human perception. For the reward function in the TD3 controller, frequency inputs need to be considered holistically in the frequency domain, which leads to more complex policy outcomes that are difficult to derive quickly. Thus, acceleration weighting can reduce the vibrations transmitted from the suspension to the vehicle body, while velocity weighting can control the suspension’s motion range and implicitly regulate the vibration frequency transmitted to the vehicle body. In the context of the vehicle suspension problem, the primary focus is on the motion states of three degrees of freedom at the vehicle’s center of mass. Among these, velocity and acceleration have a significant impact on the human body. Unlike standard DRL tasks that rely on simple goals, the reward function in this proposed framework is fundamentally designed as an optimization index with multiple objectives tailored for the dynamics of the entire vehicle. Specifically, the reward for the current action is formulated as a comprehensive weighted combination of the velocities and accelerations associated with the vertical, roll, and pitch degrees of freedom at the vehicle center of mass. The weighting coefficients are meticulously assigned based on human physiological sensitivity, prioritizing the minimization of vertical acceleration to enhance ride comfort, while simultaneously restricting roll and pitch angular accelerations to ensure handling stability. This specialized reward structure compels the TD3 agent to learn a globally optimal damping strategy across all four wheels, representing a clear methodological improvement over conventional decentralized controllers that rely primarily on localized feedback.

To further elaborate on how the reward function reflects these multi-objective requirements, the optimization goals are integrated into a cohesive weighting hierarchy. To effectively isolate the passenger cabin from road disturbances, the absolute highest penalty weights are assigned to the vertical acceleration and velocity, establishing ride comfort as the primary optimization objective. This ensures that the TD3 agent’s principal direction focuses strictly on minimizing human body vibration exposure. Simultaneously, because semi-active suspensions must also maintain the vehicle’s posture and safety, secondary penalty weights are applied to the roll and pitch angular accelerations to ensure handling stability. This deliberately prevents the agent from adopting overly soft damping strategies that would otherwise cause excessive body roll or pitch during dynamic excitations. By balancing these hierarchical weights, the reward function systematically resolves the inherent physical trade-offs in full-vehicle chassis control, preventing the DRL agent from greedily optimizing for vertical comfort at the catastrophic expense of vehicle stability. Therefore, the learned control policy is profoundly shaped by the inherent dynamic characteristics of the vehicle. By penalizing pitch and roll angular accelerations within the seven degree of freedom full vehicle model, the policy intrinsically accounts for the dynamic cross coupling effects across the longitudinal and lateral axes. This compels the TD3 agent to learn a globally coordinated damping strategy, thereby mitigating the typical waterbed effect wherein enhancing vertical ride comfort at an individual corner severely deteriorates the overall dynamic posture of the vehicle.

It is worth noting that while the reward function primarily focuses on variables related to velocity and acceleration to maximize ride comfort actuator constraints are strictly and explicitly guaranteed by the proposed state to action mapping mechanism. As shown in Eq (4.11) the arctangent activation function inherently bounds the exploratory actions within the strict physical limits of the MFD minimum and maximum currents. Furthermore instead of artificially adding penalty terms for the suspension working space and dynamic tire load into the reward function as this often leads to reward shaping complexity and conflicting gradient directions this study relies on the coupled nature of the 7 DOF vehicle dynamics. By suppressing the overall roll and pitch angular accelerations the extreme deflections at the four corners are intrinsically mitigated thus implicitly ensuring contact between the tires and the road and preventing suspension stroke saturation.

## 5. Simulation results

Based on the theory introduced earlier, simulation can be used to verify the applicability of the TD3 algorithm in the control of semi-active vehicle suspensions. In this context, MATLAB and Simulink are used to build the TD3 algorithm and the 7-DOF semi-active suspension model for the vehicle. After setting initial values, the training and validation processes are distinguished, and the simulation is performed with a fixed step size in Simulink. Pseudo-code for the simulation under road excitation is provided in Table 3.

**Table 3:** TD3 controller of vehicle 7-DOF suspension model.

Algorithm 1 TD3-based control method under vehicle 7-DOF semi-active suspension model
Initialize suspension model with parameter $D=D_{\text{0}}$. Initialize critic networks $Q_{\theta_{\text{1}}}$, $Q_{\theta_{\text{2}}}$, and actor network $\Pi_{\phi }$ with random parameters $\theta_{\text{1}}$, $\theta_{\text{2}}$, ϕ. Initialize target networks $Q_{\theta_{\text{1}}^{\prime }}$, $Q_{\theta_{\text{2}}^{\prime }}$, $\Pi_{\phi^{\prime }}$ with weight $\theta_{\text{1}}^{\prime }\leftarrow \theta_{\text{1}}$, $\theta_{\text{2}}^{\prime }\leftarrow \theta_{\text{2}}$, $\phi^{\prime }\leftarrow \phi$. Initialize replay buffer R **for** t = (0, step, T) **do** Determine training parameters doTrain and doDelayUpdate based on the current iteration. Determine the current values of road surface excitation $Y={\left[y_{\text{1}},y_{\text{2}},y_{\text{3}},y_{\text{4}}\right]}^{T}$ based on the road roughness model. $F_{si}$, $F_{ti}$, $M_{rr}$, $M_{rf}=f_{fs}\left(\varphi ,\dot{\varphi },\theta ,\dot{\theta },x_{s},{\dot{x}}_{s},X_{u},{\dot{X}}_{u},Y,\dot{Y}\right)$ Calculate Fci using the formula $F_{c}=f_{d}\left(i,x_{ls}-x_{u},{\dot{x}}_{ls}-{\dot{x}}_{u}\right)$. $\ddot{D}=f_{as}\left(F_{si},F_{ti},M_{rr},M_{rf},F_{ci},m_{s},m_{u}\right)$ update D, $\dot{D}$ using $\ddot{D}$ **if** doTrain = 1 **then** update s using D, $\dot{D}$, $\ddot{D}$ Store state as $s^{\prime }$, store reward as $r=R\left(s^{\prime }\right)$, store transition tuple $\left(s,a,r,s^{\prime }\right)$ in R Sample mini-batch from R $a^{\prime }=\Pi_{\phi^{\prime }}\left(s^{\prime }\right)$, Update critics $Q_{\theta_{j}}$ **if** doDelayUpdate = 1 **then** Update ϕ by $\nabla_{\phi }J\left(\phi \right)$ Update target networks **end if** Select next action $a^{\prime }=\Pi_{\phi }\left(s^{\prime }\right)+\varepsilon$. Store $a=a^{\prime }$, $s=s^{\prime }$. Calculate output current $I_{i}$. **end if** **end for**

### 5.1. MFD model

**Fig 3:**
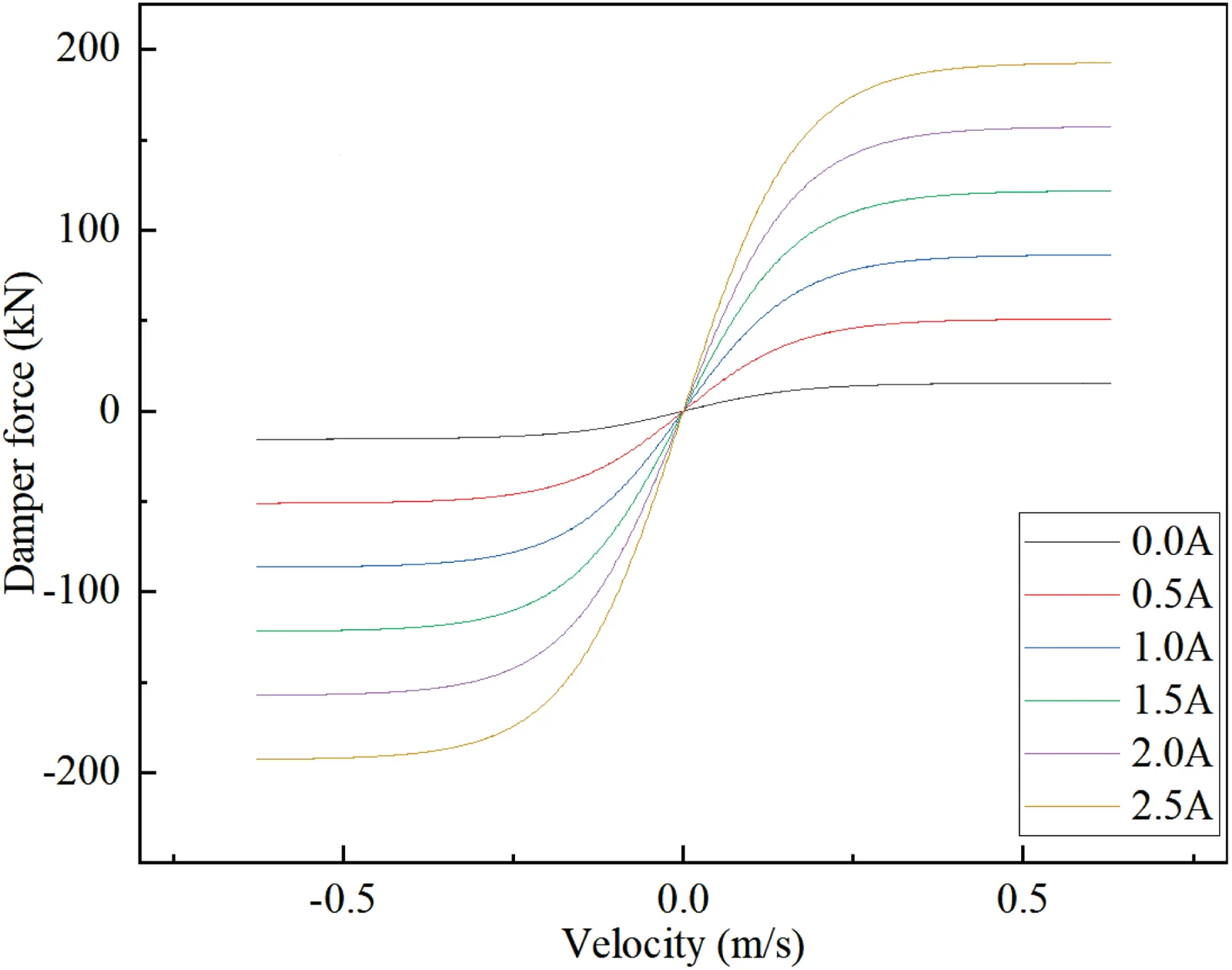
The mechanical properties of the MFD model.

In the model used in this paper, the semi-active suspension system utilizes an MFD, which exhibits variable damping forces as a response to changes in electrical current. Fig 3 illustrates the relationship between the damping force and the variations in velocity and electrical current, and the specific damping force formula is provided in the reference [47]:


$$\begin{array}{c} \left\{ \begin{array}{c} @c@f_{yi}=70.86I_{i}+15.2\\ c_{pi}=0.105I_{i}+0.609\\ F_{ci}=f_{yi}tanh\left(6\cdot \left({\dot{x}}_{si}-{\dot{x}}_{ui}\right)\right)+c_{pi}\left({\dot{x}}_{si}-{\dot{x}}_{ui}\right)-0.126\left(x_{si}-x_{ui}\right) \end{array}\right. \end{array}$$ (5.1)

The hysteresis characteristics of the MFD were neglected in this study to reduce computational complexity. It is acknowledged that real world MFDs exhibit strong rate dependent hysteresis and omitting this is a simplification. However incorporating complex differential equation based hysteretic models like the Bouc Wen model into a highly coupled 7 DOF full vehicle environment would exponentially increase the computational burden and potentially destabilize the gradient convergence of the deep reinforcement learning training process. Since the primary focus of this study is the multi objective posture coordination of the full vehicle dynamics namely minimizing vertical roll and pitch accelerations and the validation of the continuous state action mapping mechanism the hyperbolic tangent approximation is deemed an appropriate compromise. It effectively captures the primary physical bounds specifically maximum and minimum damping forces and the macroscopic nonlinear saturation characteristics of the MFD which provides a sufficient dynamic baseline for evaluating the global control policy of the TD3 agent at this theoretical stage. Where $f_{yi}$ represents the maximum damping force described using a hyperbolic tangent function, $c_{pi}$ is the linear damping component of the MFD, and the remaining terms in the formula represent the equivalent spring force in the damper. The MFD used in this paper has a maximum current of $I_{max}=3\text{A}$ and a minimum current of $I_{min}=0\text{A}$. Fig 3 shows the relationship between the velocity, the damping force, and the current.

### 5.2. Condition of simulation

The simulation primarily considers two operating conditions: the training process of DRL and the testing process. In the simulation, a lower simulation time step is used for the vehicle suspension simulation to achieve more precise control. It is worth noting that the simulation time step is set to 0.01 s. This discrete step size intrinsically reflects the typical physical response delay of practical magnetorheological fluid dampers (MFDs), which generally operate with a millisecond-level response time. Thus, a basic level of discrete actuator delay has been implicitly incorporated into the control loop. Furthermore, the online execution of the trained Actor network requires only a single forward pass per time step. With approximately 10,000 network parameters that require roughly 40 KB of memory and 20,000 floating-point operations, the computational time on a standard cost-effective microcontroller is well within the 0.01 s discrete step size, ensuring real-time control feasibility. The parameters for the vehicle model are determined as shown in Table 4. In realistic physical environments, vehicle parameters are inevitably subjected to uncertainties, which primarily originate from changes in passenger loading [44]. For instance, assuming a conservative average passenger weight of approximately 80 kg within a four-seat vehicle configuration, the variation of the sprung mass can reach up to 320 kg [44]. Consequently, the parameter variations in this study are fundamentally determined based on these sprung mass fluctuations and their corresponding impact on the moments of inertia [44]. The specific parameter boundaries for the sprung mass and the inertia variations are explicitly defined in Table 4 to guarantee the robustness of the physical model under realistic operating conditions. Furthermore, the TD3 algorithm in the simulation includes the critic network and actor networks presented in Tables 5 and 6. The Actor and Critic networks are not determined arbitrarily: starting with two-layer networks, we tested results with different numbers of layers and nodes (in increments of 25). For the Critic network, two fully connected layers are sufficient, with the number of nodes being critical. Typically, a two-layer network with the same number of nodes per layer is used. Through testing, 75 nodes per layer achieved relatively high computational efficiency, and after fine-tuning, we adopted 100 nodes in the first layer and 75 nodes in the second layer to ensure convergence. Since the Actor network involves similar computations, it also uses the same first and second layers as the Critic network. Finally, the output must use an arctan layer to ensure that the output range is reliable. The hyperparameters of the TD3 controller were determined through multiple trial-and-error experiments. All hyperparameters are based on the actual hyperparameters of the model uploaded to the platform. The current hyperparameters are derived from the first training process that achieved relatively good convergence results. In that initial process, the number of cycles required for satisfactory convergence was approximately 2,500 (each training cycle simulated 30 seconds of suspension time), with a total training duration of two and a half days. After several adjustments, the current hyperparameters are capable of achieving good convergence within 1,000 cycles. The model has not yet undergone ablation experiments. Under these settings, the paper considers the following road conditions: ISO-specified A-class road and C-class road. The operating conditions used in this paper include: driving at a speed of 60 km/h on an A-class road, driving at a speed of 120 km/h on an A-class road, and driving at a speed of 60 km/h on a B-class road.

**Table 4:** Parameters of vehicle suspension model [44].

Parameter	Value	Uncertainty (Δ)	Unit	Parameter	Value	Uncertainty (Δ)	Unit
$k_{s}$	20000	0	N/m	$m_{s}$	1500	165	kg
$k_{t}$	200000	0	N/m	$m_{u}$	28	N/A	kg
$k_{r}$	10000	0	N/m	$I_{y}$	1423.8	149.16	kg·m^2^
$b_{\text{1}},b_{\text{2}}$	0.71	N/A	m	$I_{z}$	504	52.8	kg·m^2^
$a_{\text{1}}$	0.91	N/A	m	g	9.8	N/A	m/s^2^
$a_{\text{2}}$	1.44	N/A	m				

**Table 5:** Specification of critic network.

Name of layer	Type	Input	Number of neurons
Observer layer1	Fully connected	Observer	100
ReLU1	ReLU	Sub_layer1	100
Observer layer2	Fully connected	Relu1	75
Act layer	Fully connected	Action	75
Addition layer	Addition	(Observer layer2, Act layer)	75
ReLU2	ReLU	Addition layer	75
Out layer	Fully connected	ReLU2	1

**Table 6:** Specification of actor network.

Name of layer	Type	Input	Number of neurons
Layer1	Fully connected	Observer	100
ReLU1	ReLU	Layer1	100
Layer2	Fully connected	ReLU1	75
ReLU2	ReLU	Layer2	75
Layer3	Fully connected	Relu2	4
Tanh layer	tanh	Layer3	4

For these conditions, the paper investigates the control performance of the TD3 algorithm compared to passive suspension and Skyhook control methods. In the passive suspension, the fixed MFD current is set to 1.5A. The Skyhook control method, through multiple simulations, uses the optimal linear Skyhook control results.

**Fig 4:**
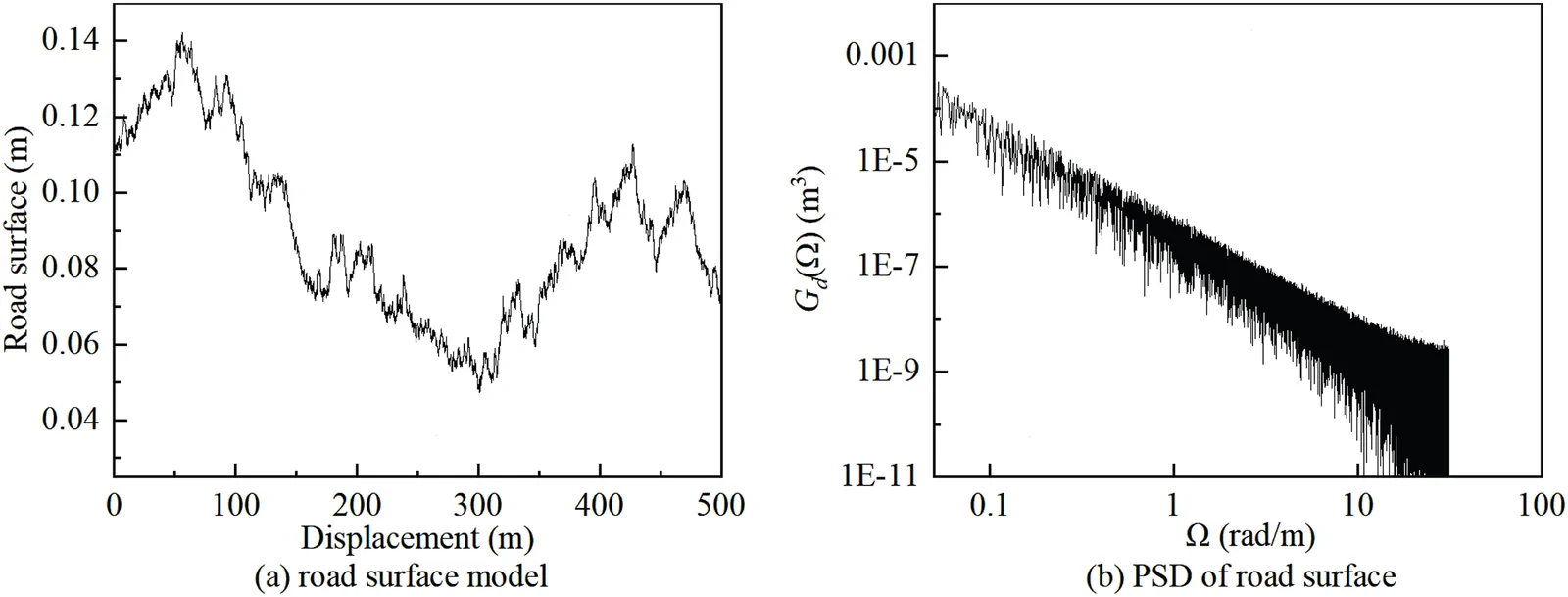
Road surface model.

In vehicle driving excitation, the excitation models used in simulations for Class A and Class B road surfaces are determined by the road surface power spectral density model established by ISO8608. Fig 4 displays the road surface excitation model used for the left front wheel of a vehicle in simulation on a Class A road surface, with the PSD graph on the right indicating that the excitation-displacement profile satisfies the ISO 8608 specification.

When assessing passenger comfort, considering that the human body is most sensitive to the vertical accelerations experienced, our priority is to reduce the vertical accelerations at the vehicle’s center of gravity. Simultaneously, minimizing the angular accelerations at the vehicle’s center of gravity is also desired. To accurately evaluate this ride comfort, the frequency-weighted root mean square (RMS) acceleration is calculated in accordance with the ISO 2631–1:1997 standard and its 2010 amendment [48]. Specifically, the vertical acceleration of the sprung mass is evaluated using the $W_{k}$ frequency weighting filter. To facilitate efficient continuous-time domain simulation, a highly accurate third-order approximation of the $W_{k}$ weighting filter [49] was adopted in the MATLAB model. Furthermore, to comprehensively assess the rotational dynamics affecting passenger comfort, the roll and pitch angular accelerations are evaluated using the $W_{e}$ frequency weighting network. The $W_{e}$ filter was constructed based on the exact parameters specified in Appendix A of the ISO 2631−1 standard [48] ($f_{\text{1}}=0.4$ Hz, $f_{\text{2}}=100$ Hz, $f_{\text{3}}=1.0$ Hz, $f_{\text{4}}=1.0$ Hz, $Q_{\text{4}}=0.63$).

### 5.3. The simulation results of class A road surface at 60 km/h excitation

**Fig 5:**
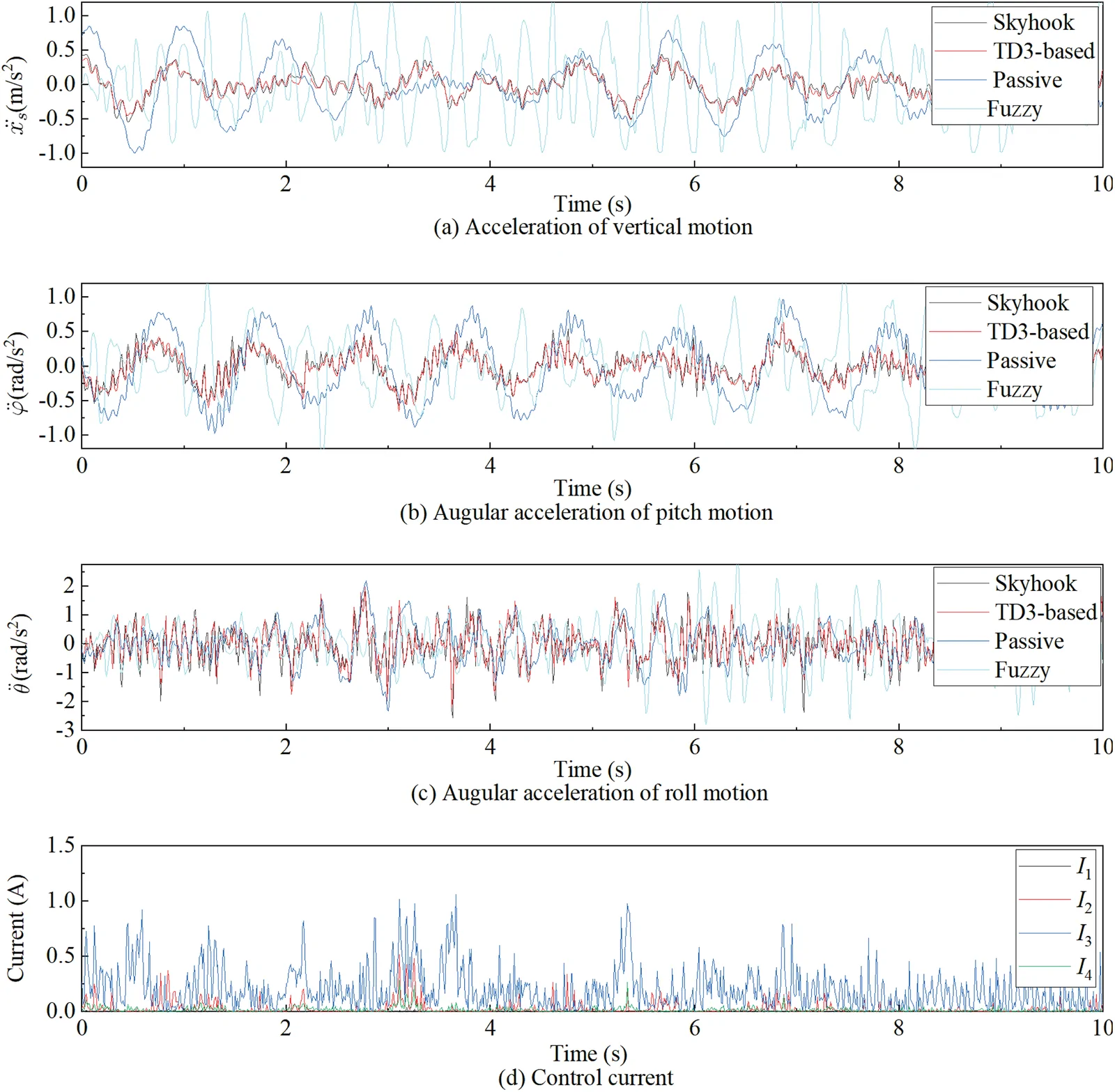
Results of condition: Class A(1).

Under the excitation of a Class A road surface at a vehicle speed of 60 km/h, the vehicle suspension operates under relatively smooth conditions. Fig 5 illustrates the response at the vehicle’s center of gravity under this scenario.

Upon calculation, at the vehicle’s center of gravity, the overall average vertical acceleration using the TD3-based control method is 49.41% lower compared to the passive suspension and 9.33% lower compared to the skyhook control method. This suggests that the TD3-based control method is effective in reducing vertical acceleration, contributing to improved ride comfort when compared to both the passive suspension and the skyhook control method. Meanwhile, the angular acceleration differs only slightly compared to the controller using the skyhook control method. This indicates that under these conditions, employing the TD3-based control method can provide passengers with better ride comfort. Additionally, Fig 5(d) displays the control currents at the four MFDs.

Within the time range shown in Fig 5, it can be observed that under the condition of smooth road surfaces and lower vehicle speeds, the vertical acceleration output from the passive suspension consistently has a higher amplitude. Vehicles with suspensions controlled using the skyhook method and the TD3-based control method exhibit similar trends in vertical response. However, the TD3-based control method responds faster to changes in road surface excitation, and in some regions with larger angular acceleration amplitudes, the TD3-based control method has a positive impact on reducing vertical acceleration. This may be related to the current control strategy of the skyhook control method being dependent on velocity. Although the MFD model linearizes the current for the skyhook control method, which is advantageous, the results obtained by using velocity for control and linear mapping are still relatively conservative. In the full-vehicle model, the TD3-based control method receives inputs from all vehicle degrees of freedom, enabling more accurate control of the vehicle’s body state, and reducing some of the vertical acceleration due to body rotation. Since the reward function of the TD3-based control method tends to decrease the overall vertical acceleration, there may be regions where angular accelerations are higher in the other two degrees of freedom. However, these regions usually correspond to areas where vertical acceleration is reduced, contributing to improved ride comfort. Also, in the control current curve, it is noted that the control current of the third MFD varies significantly, influenced by the convergence direction of DRL, which is inevitable in the model established in this paper. More importantly, these control current profiles demonstrate how the inherent damper nonlinearities actively influence the learned policy. As mathematically described in Eq. (5.1), the MFD exhibits strong nonlinear characteristics, particularly near its saturation boundaries represented by the hyperbolic tangent function. Through continuous environmental interactions, the TD3 agent implicitly learns to navigate this physical nonlinearity. Rather than generating aggressive, unbounded current commands that would fall into the unresponsive saturation zones of the damper, the learned policy adapts by continuously adjusting the control current to operate preferentially within the sensitive pseudo-linear regions of the force-velocity profile of MFD, thereby maximizing vibration isolation efficiency.

In addition to the passive suspension and the Skyhook method Fig 5 also presents the response of the Fuzzy control method. As illustrated in Fig 5 the red curve representing the proposed TD3 based method consistently achieves the smallest acceleration amplitudes and the smoothest trajectory among all compared strategies. Although the Fuzzy control method shows a certain degree of vibration reduction compared to the passive suspension its dynamic response exhibits larger peak fluctuations than the proposed method. This occurs because the fixed rule base of the Fuzzy controller lacks sufficient adaptiveness when dealing with the complex multi axis coupling of the 7 DOF full vehicle model. Consequently the proposed TD3 based method demonstrates superior suppression effectiveness and dynamic stability under road surface excitation.

### 5.4. The simulation results of class A road surface at 120 km/h excitation

**Fig 6:**
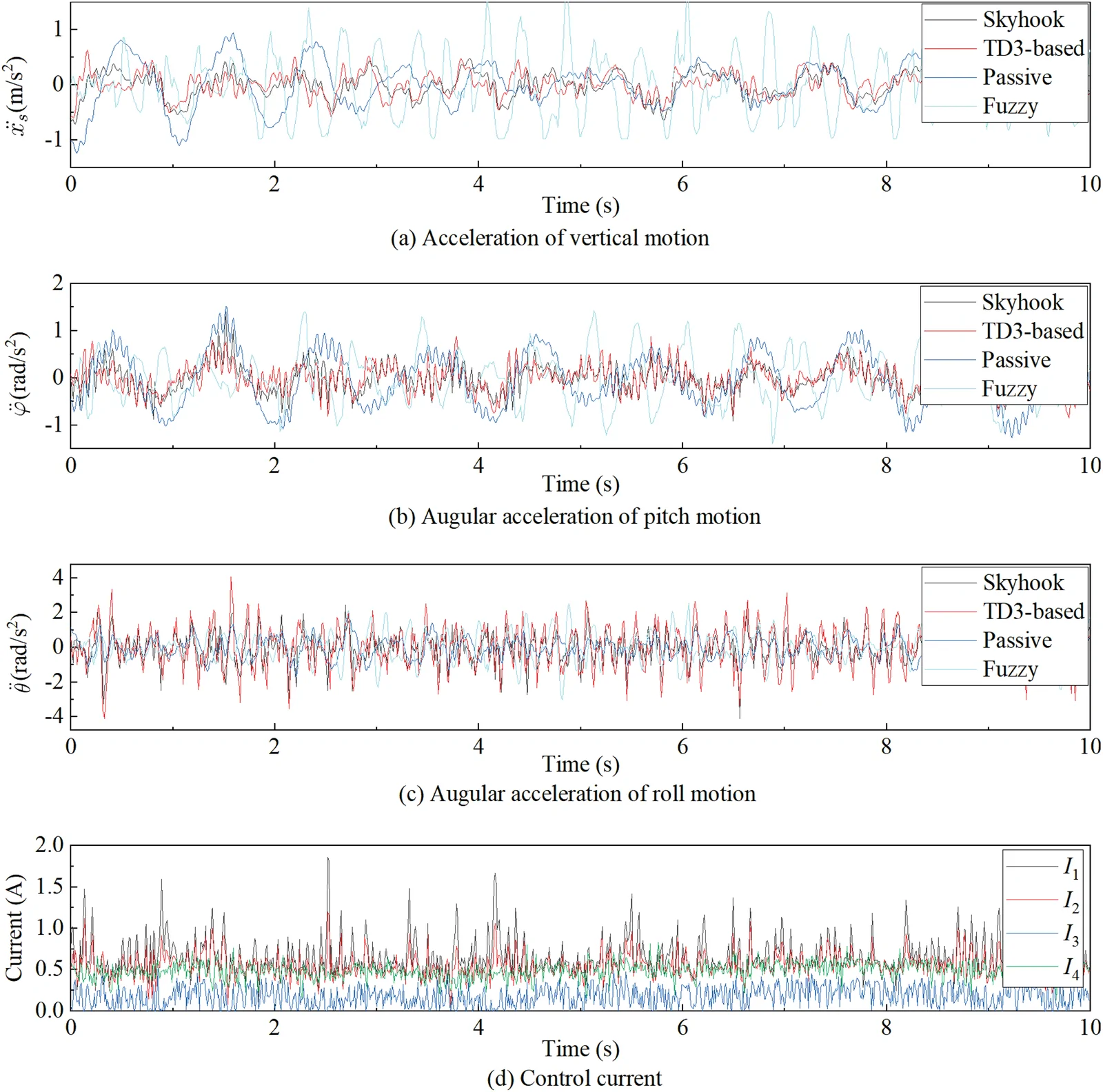
Results of condition: Class A(2).

Under the excitation of a Class A road surface at a speed of 120 km/h, the vehicle suspension experiences higher-frequency excitation, leading to increased velocity and acceleration of the vehicle body’s non-sprung mass displacement. Under such conditions, Fig 6 illustrates the response at the vehicle’s center of gravity.

At high speeds, at the vehicle’s center of gravity, the overall average vertical acceleration using the TD3-based control method is 24.82% lower compared to the passive suspension. While it exhibits a distinct dynamic response profile compared to the skyhook control method under high-frequency excitations due to multi-axis posture coordination, the vertical ride comfort remains highly competitive. In the calculation of longitudinal angular acceleration, there’s an observed increase when using the TD3-based control method, possibly due to an overall improvement reward. In reality, the vertical acceleration for most passenger positions in the vehicle using the TD3-based control method remains generally lower than when using the skyhook control method.

Under high-speed driving conditions, the passive suspension still exhibits the poorest performance in terms of vertical acceleration. However, unlike slow-speed driving, there is a significant difference in the control effects between the TD3-based control method and the skyhook control method. This difference is primarily reflected in the handling capability of the entire vehicle model. Within the range shown in the graph, the TD3-based control method, compared to the skyhook control method, can significantly reduce vertical acceleration in most states. It’s also evident that, even under high-speed driving conditions with relatively smooth road surface states, the control current does not reach its maximum. The value of the control current still has some correlation with the convergence direction.

Similarly as illustrated in Fig 6 the Fuzzy control method struggles to effectively suppress the high frequency excitations at 120 km/h resulting in consistently higher vertical acceleration peaks than the proposed algorithm. Because the predefined fuzzy rules cannot rapidly adapt to the intensified road inputs the proposed TD3 based control method demonstrates significantly superior high speed handling capability and continuous adaptiveness compared to the conventional Fuzzy control method.

### 5.5. The simulation results of the class B road surface at 60 km/h excitation

**Fig 7:**
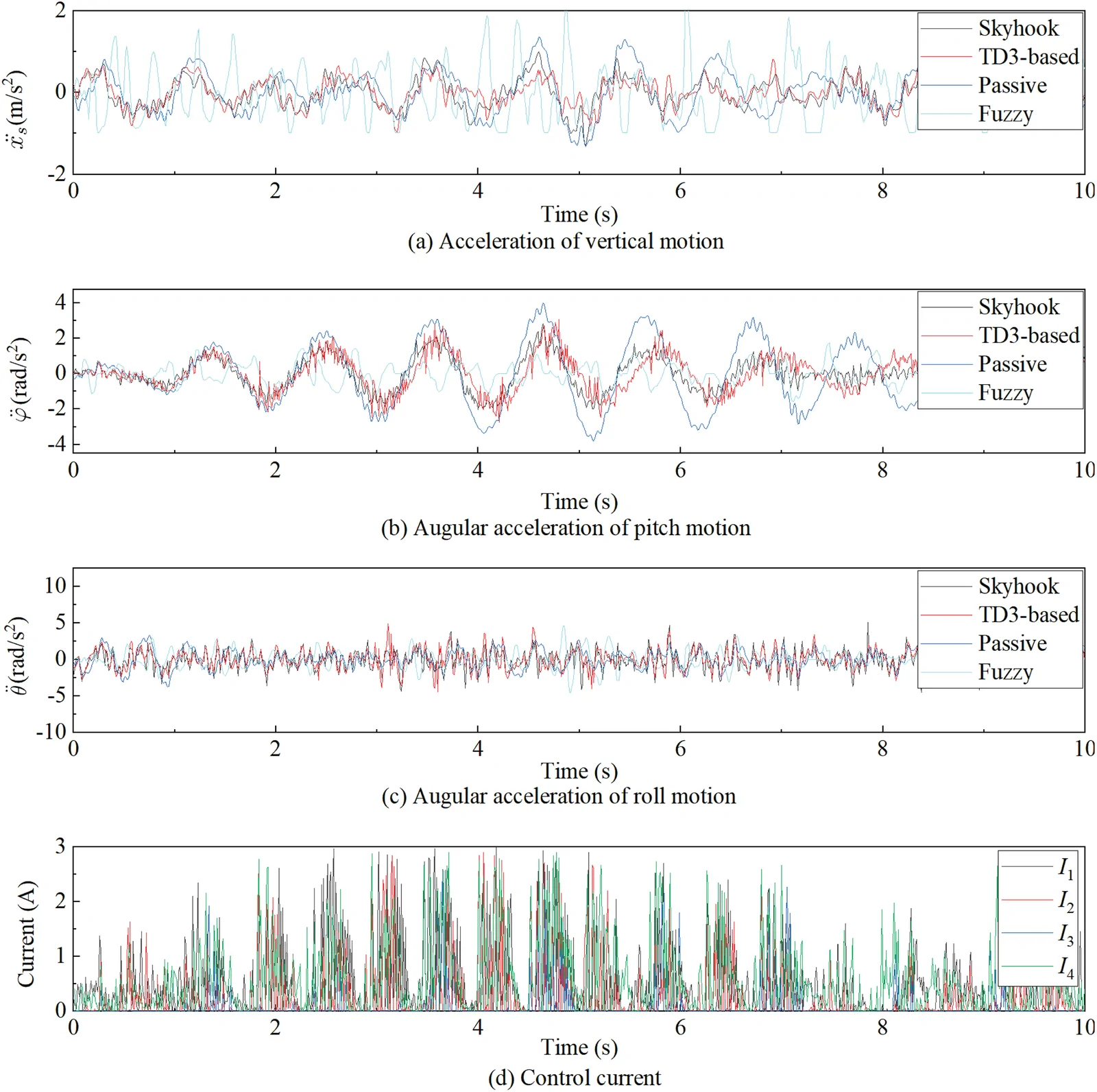
Results of condition: Class B.

Under the conditions of a Class B road surface, Fig 7 displays the vehicle’s center of gravity response under three different control methods. At the vehicle’s center of gravity, the overall average vertical acceleration using the TD3-based control method is 19.76% lower compared to the passive suspension and 3.19% lower compared to the skyhook control method.

Simultaneously, this scenario illustrates that while the overall data in the roll direction is better with the TD3-based control method than the skyhook control method, due to overfitting in the output values of DRL, certain angular accelerations reach higher peaks, weakening the control effectiveness. Fig 7(c) precisely showcases this overfitting region in the data.

Within the displayed time range in the graph, it is evident that, although the passive suspension still performs poorly, it exhibits similar trends in the low-frequency range to the other two suspension systems. The graph indicates that the TD3-based control method, overall, outperforms the skyhook control method, demonstrating the advantages of using the entire vehicle model on rough road surfaces. Additionally, due to the expanded range of damping force output, the changes in control current are more pronounced, highlighting the effectiveness of the TD3-based control method in providing better control.

Under the harsher Class B road conditions depicted in Fig 7 the conventional Fuzzy control method shows pronounced oscillations and fails to optimally coordinate the multi axis dynamics. Due to the severe road undulations the fixed rule base of the Fuzzy controller becomes inadequate. In contrast the proposed TD3 based control method significantly outperforms the Fuzzy control method by maintaining a more stable vehicle body posture and effectively suppressing extreme acceleration peaks which further validates its exceptional robustness on rough road surfaces.

### 5.6. Comprehensive performance evaluation based on ISO 2631−1

To provide a comprehensive evaluation of ride comfort under different operating conditions in accordance with the ISO 2631−1 standard, the frequency-weighted RMS accelerations (including vertical $W_{k}$ and rotational $W_{e}$ indices) for the passive suspension, the classical linear Skyhook control, and the proposed TD3-based method are summarized in Table 7.

**Table 7:** Frequency-weighted RMS accelerations of vehicle suspension under different operating conditions.

Operating Condition	Control Method	Vertical ($W_{k}$) RMS ($m/s^{2}$)	Roll ($W_{e}$) RMS $rad/s^{2}$	Pitch ($W_{e}$) RMS $rad/s^{2}$
Class A (60 km/h)	Passive	0.1939	0.3907	0.2610
	Skyhook	0.1082	0.1462	0.1377
	TD3-based	0.0981	0.1532	0.1414
	Fuzzy	0.4889	0.1893	0.1456
Class A (120 km/s)	Passive	0.2031	0.5016	0.2200
	Skyhook	0.1333	0.1939	0.1442
	TD3-based	0.1527	0.1587	0.1800
	Fuzzy	0.4515	0.2341	0.1591
Class B (60 km/h)	Passive	0.2647	1.5594	0.4938
	Skyhook	0.2194	0.7359	0.2716
	TD3-based	0.2124	0.8378	0.3371
	Fuzzy	0.5366	0.2782	0.2453

As comprehensively presented in Table 7, the traditional Fuzzy control method yields vertical frequency-weighted RMS values of 0.4889 (Class A, 60 km/h), 0.4515 (Class A, 120 km/h), and 0.5366 (Class B, 60 km/h). While the Fuzzy controller operates stably within the predefined rule base, its performance in global multi-axis posture coordination (roll and pitch angles) remains constrained. In contrast, the proposed TD3-based method dynamically balances vertical acceleration with rotational angular accelerations through its multi-objective reward mechanism, achieving significantly lower RMS indices across the evaluated scenarios.

### 5.7. Evaluation of suspension working space and tire-road contact

To address potential concerns regarding the safety constraints of the suspension system, the dynamic tire load (DTL) and suspension working space (SWS) are systematically evaluated. Although these objectives were not explicitly penalized in the reward function, the comprehensive posture optimization of the 7-DOF model implicitly maintains these constraints.

**Fig 8:**
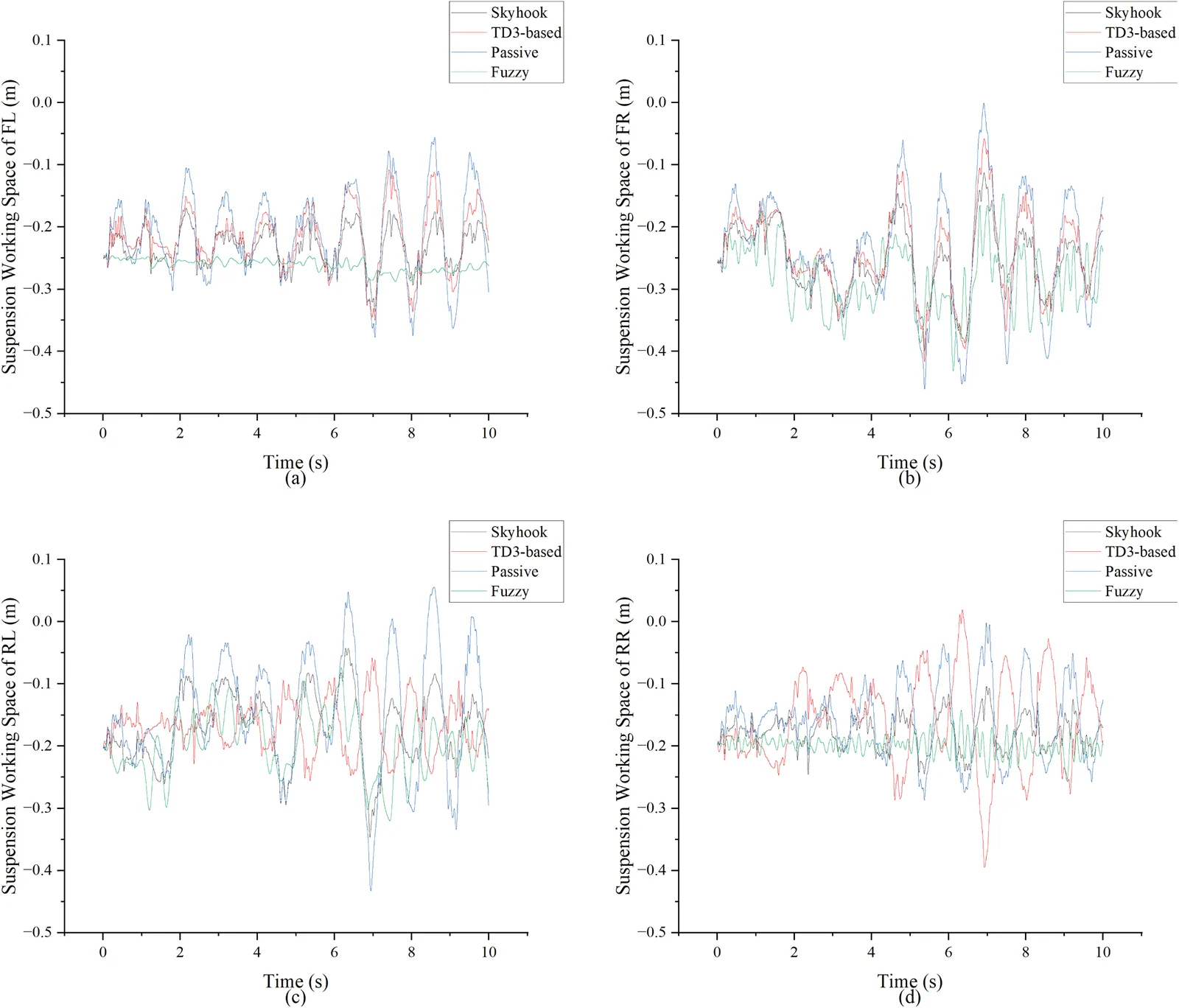
Time-domain responses of suspension working space (SWS) under Class A road excitation at 60 km/h (Condition 1). (a) FL wheel. (b) FR wheel. (c) RL wheel. (d) RR wheel.

**Fig 9:**
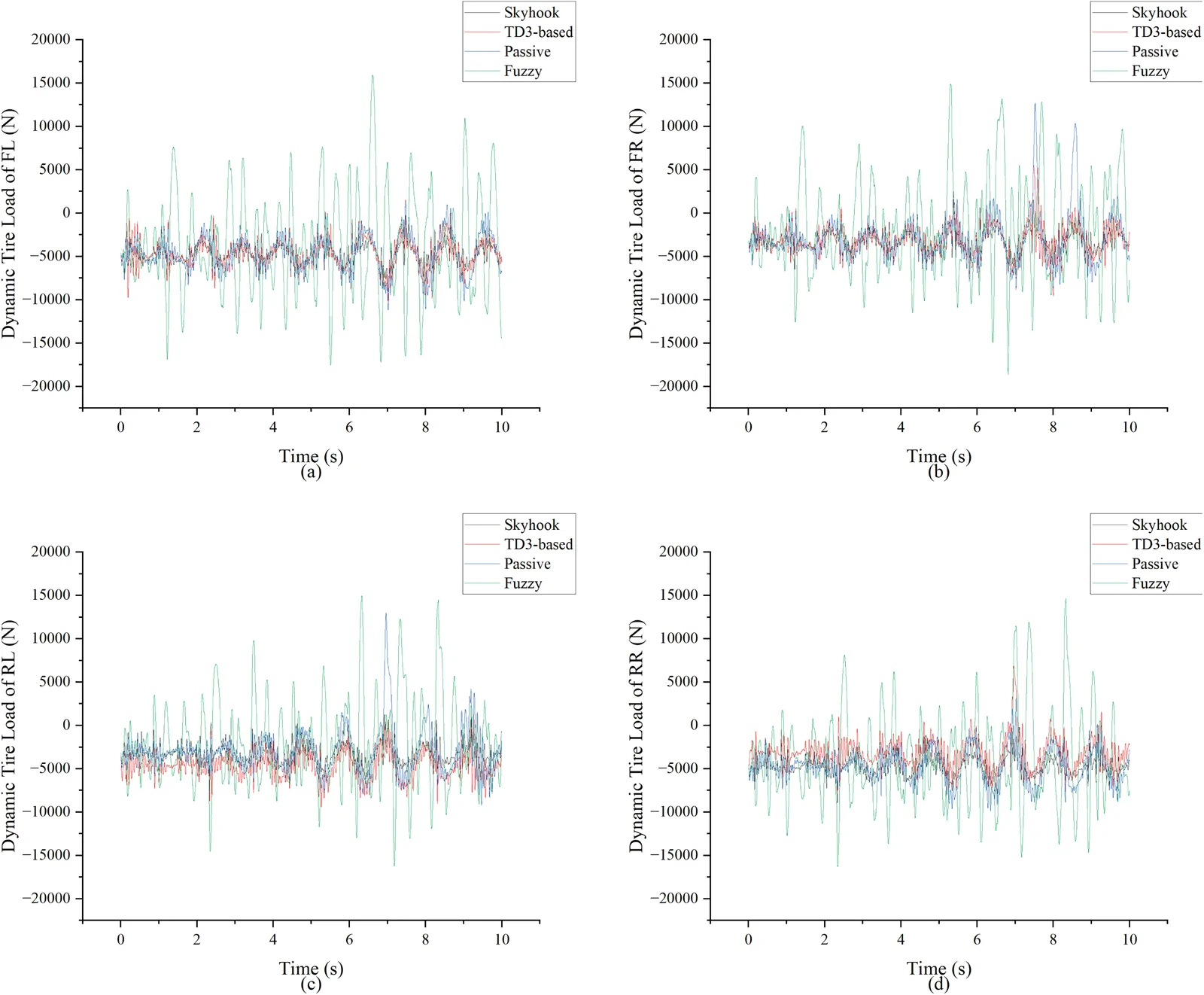
Time-domain responses of dynamic tire load (DTL) under Class A road excitation at 60 km/h (Condition 1). (a) FL wheel. (b) FR wheel. (c) RL wheel. (d) RR wheel.

**Fig 10:**
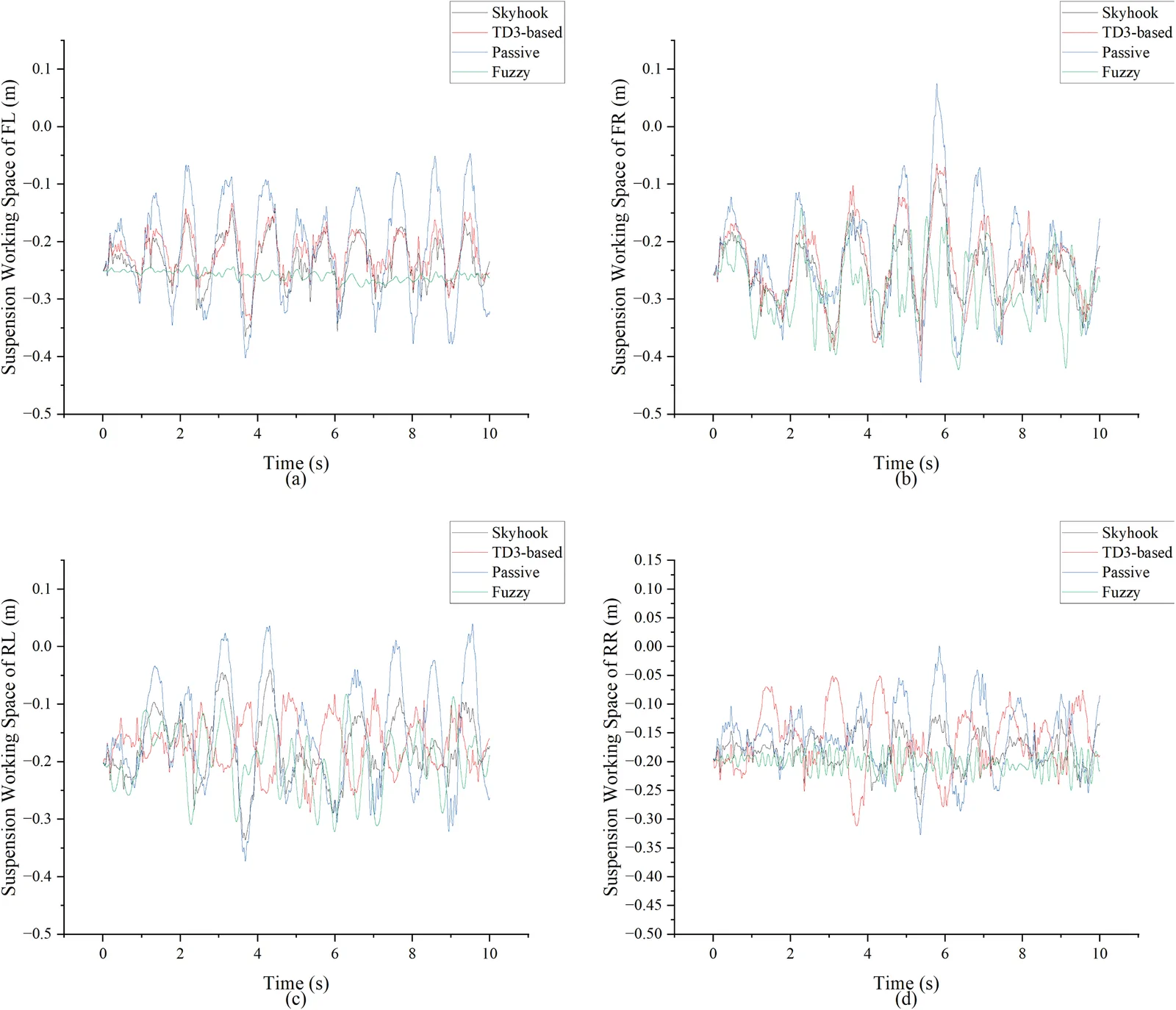
Time-domain responses of suspension working space (SWS) under Class A road excitation at 120 km/h (Condition 2). (a) FL wheel. (b) FR wheel. (c) RL wheel. (d) RR wheel.

**Fig 11:**
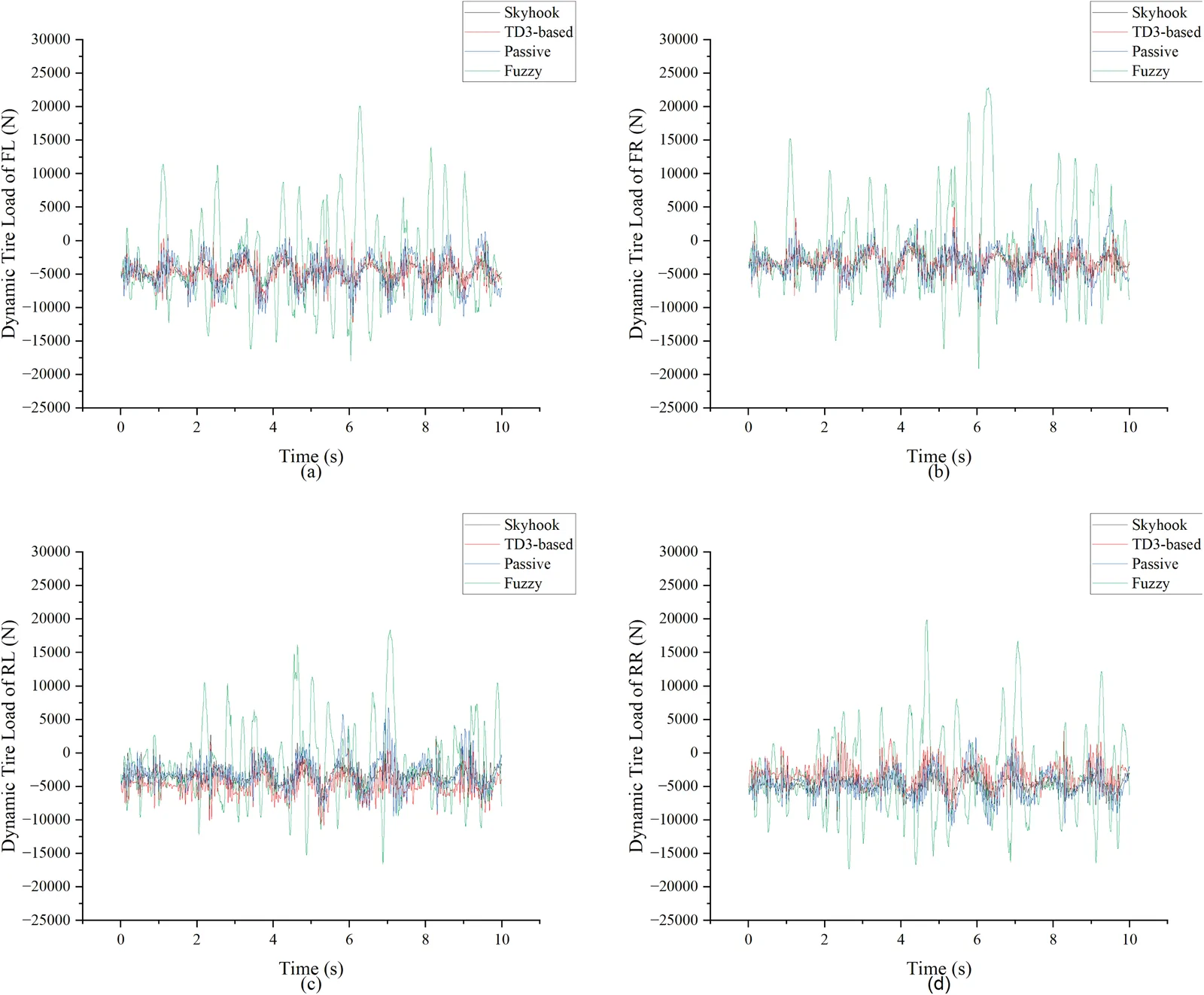
Time-domain responses of dynamic tire load (DTL) under Class A road excitation at 120 km/h (Condition 2). (a) FL wheel. (b) FR wheel. (c) RL wheel. (d) RR wheel.

**Fig 12:**
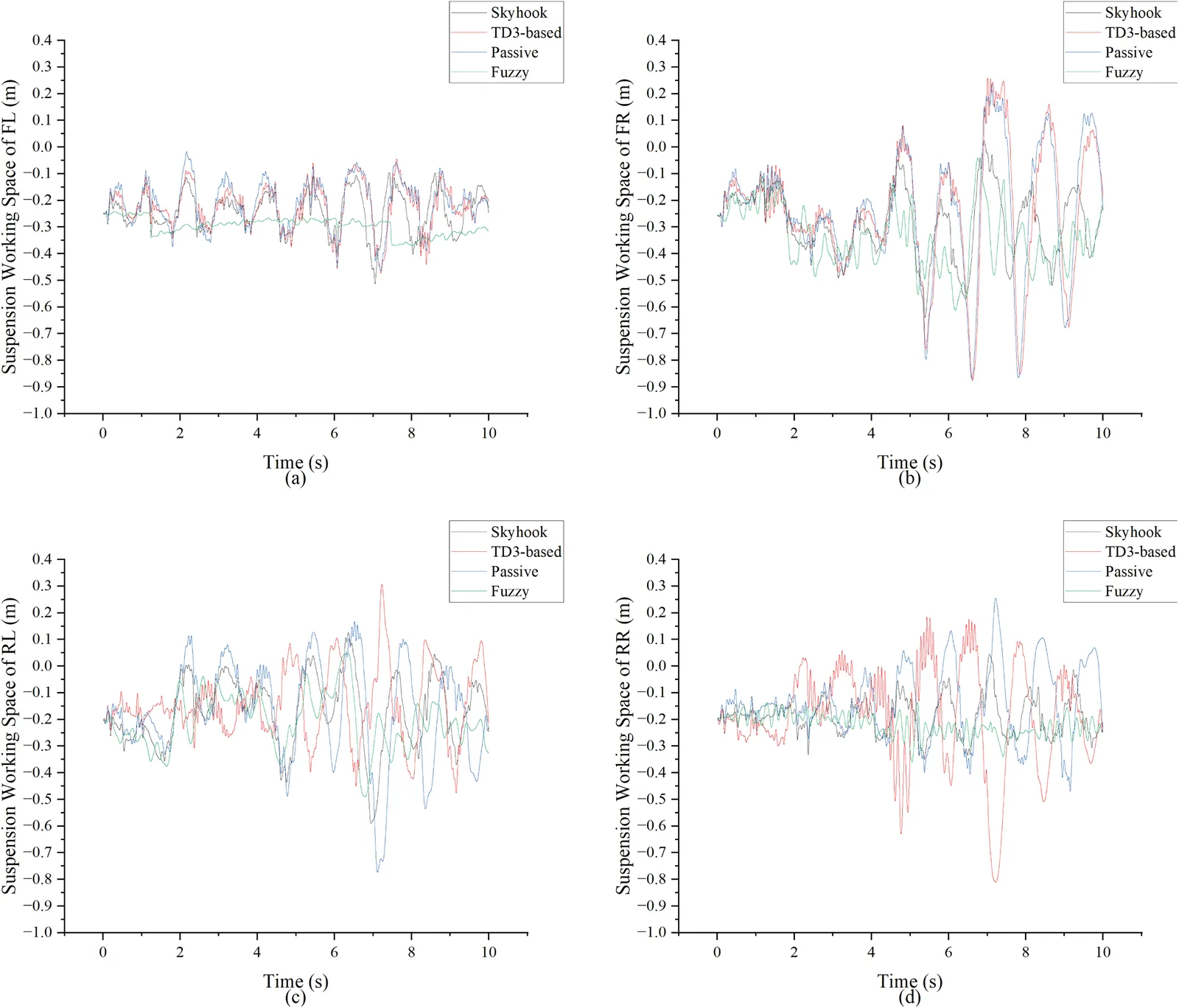
Time-domain responses of suspension working space (SWS) under Class B road excitation at 60 km/h (Condition 3). (a) FL wheel. (b) FR wheel. (c) RL wheel. (d) RR wheel.

**Fig 13:**
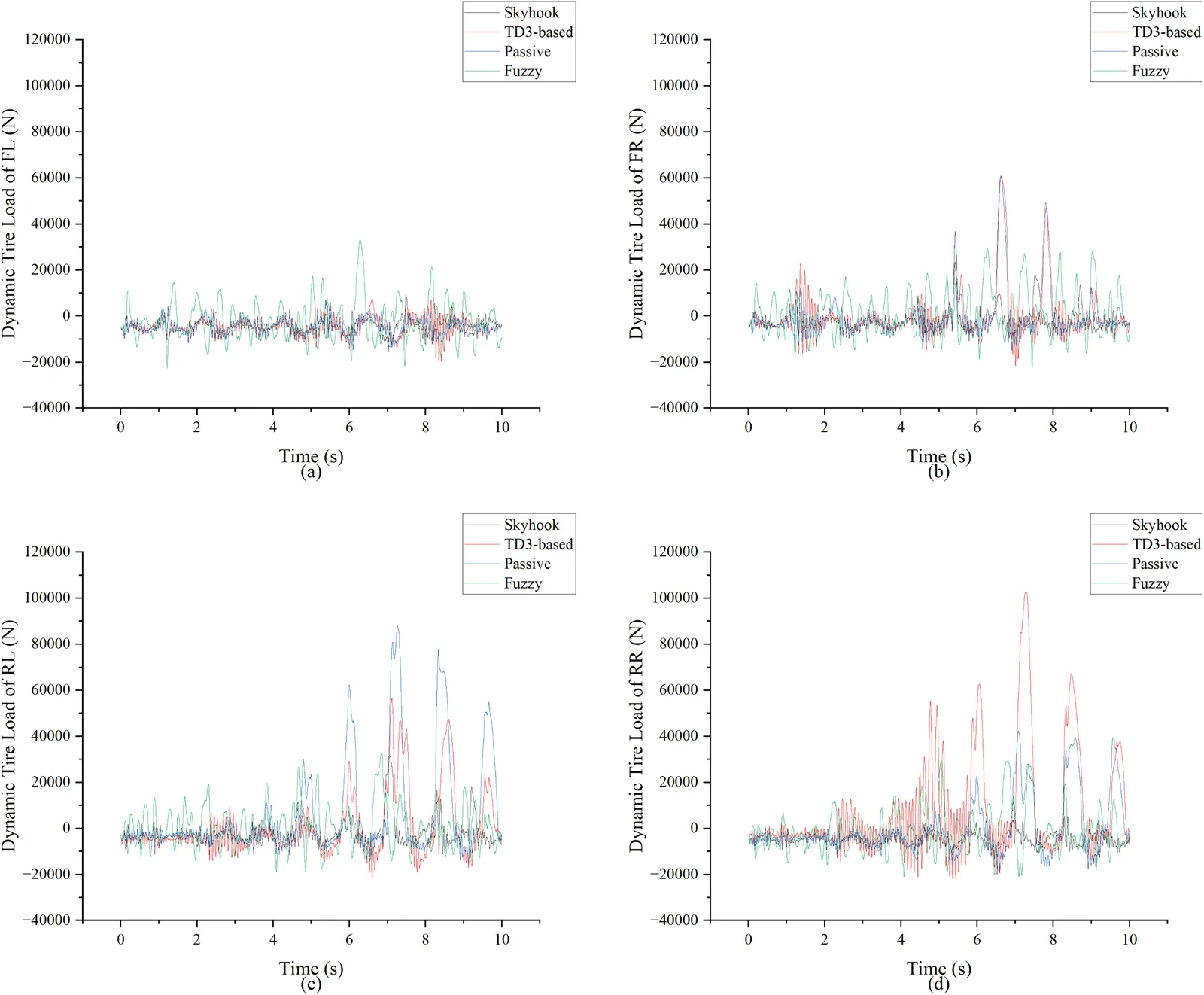
Time-domain responses of dynamic tire load (DTL) under Class B road excitation at 60 km/h (Condition 3). (a) FL wheel. (b) FR wheel. (c) RL wheel. (d) RR wheel.

To quantitatively verify the performance across all simulated scenarios, the root mean square (RMS) values of SWS and DTL under three typical conditions are summarized in Tables 8 and 9, respectively. Furthermore, the complete time-domain responses of SWS and DTL for the four wheels (FL, FR, RL, RR) are illustrated in Figs 8–13.

**Table 8:** Comparison of the RMS values of suspension working space (SWS) under different driving conditions.

Driving Condition	Position	RMS (m)			
		Passive	Skyhook	TD3-based	FUZZY
Class A (60 km/h)	FL	0.2206	0.2331	0.2229	0.2612
	FR	0.2539	0.2605	0.2517	0.2873
	RL	0.1763	0.1783	0.1754	0.2031
	RR	0.1679	0.1799	0.2242	0.2002
Class A (120 km/h)	FL	0.2285	0.2394	0.1731	0.2591
	FR	0.2500	0.2600	0.2509	0.2883
	RL	0.1794	0.1854	0.1759	0.2059
	RR	0.1678	0.1819	0.1739	0.2024
Class B (60 km/h)	FL	0.2291	0.2539	0.2354	0.2978
	FR	0.3291	0.3141	0.3326	0.3559
	RL	0.2511	0.2149	0.1941	0.2328
	RR	0.1899	0.2008	0.2652	0.2254

**Table 9:** Comparison of the RMS values of dynamic tire load (DTL) under different driving conditions.

Driving Condition	Position	RMS (N)			
		Passive	Skyhook	TD3-based	FUZZY
Class A (60 km/h)	FL	5062.93	4805.45	4937.27	6929.79
	FR	3993.74	3400.79	3594.86	5830.49
	RL	3931.91	3415.10	4912.57	5338.41
	RR	5076.81	4821.48	3611.20	6560.95
Class A (120 km/h)	FL	5271.74	4931.35	4989.81	7345.98
	FR	3897.24	3528.26	3582.37	7015.60
	RL	3830.92	3478.21	4959.32	5978.93
	RR	5125.91	4881.49	3634.92	7146.13
Class B (60 km/h)	FL	5675.98	5401.11	5894.40	8920.70
	FR	11138.51	5047.70	11779.85	9808.07
	RL	22891.64	6067.07	13965.92	9022.89
	RR	12594.65	5426.79	24778.73	9910.89

As presented in Table 8 and visually confirmed by Figs 8 and 10, under standard smooth road conditions, including a normal speed of 60 km/h and a high speed of 120 km/h, the proposed TD3 algorithm effectively restricts the SWS fluctuations within optimal physical bounds. For instance on the Class A road at 60 km/h the RMS of the TD3 method on the front left wheel is 0.2229 m which is smaller than that of the Skyhook control with 0.2331 m and the Fuzzy control with 0.2612 m. Similarly, as shown in Table 9 and Figs 9 and 11, the dynamic tire loads maintain highly stable trajectories during both low-speed and high-speed driving on Class A roads. The RMS values of the TD3 method typically range from 3500 N to 5300 N which are comparable to the vehicle static gravitational load whereas the Fuzzy control exhibits larger fluctuations up to 7345 N thereby implicitly ensuring continuous contact with the road surface without requiring explicit penalty terms.

However, an insightful control trade-off is revealed under the harsher Class B road excitation at 60 km/h. As the reward function primarily drives the agent to minimize the sprung mass accelerations, the TD3 algorithm actively allocates much larger dynamic loads to individual wheels to stabilize the vehicle body. Consequently as observed in Table 9 and clearly depicted by the extreme transient peaks in Fig 13 the DTL responses of TD3 exhibit significant increases on the rear left and front right wheels with DTL RMS values reaching up to 24778 N and 11779 N respectively compared to the conservative Skyhook and Fuzzy methods. Although the SWS limits are still physically maintained (Fig 12), this extreme operating condition demonstrates that while unconstrained DRL models excel at isolating vibrations on standard roads, they tend to aggressively sacrifice tire force stability to maximize ride comfort under severe excitations. This finding validates the importance of actuator constraints and indicates that explicitly incorporating DTL and SWS into the multi-objective reward function will be a crucial step for the future physical deployment of DRL-based active suspensions.

### 5.8. Frequency domain analysis

**Fig 14:**
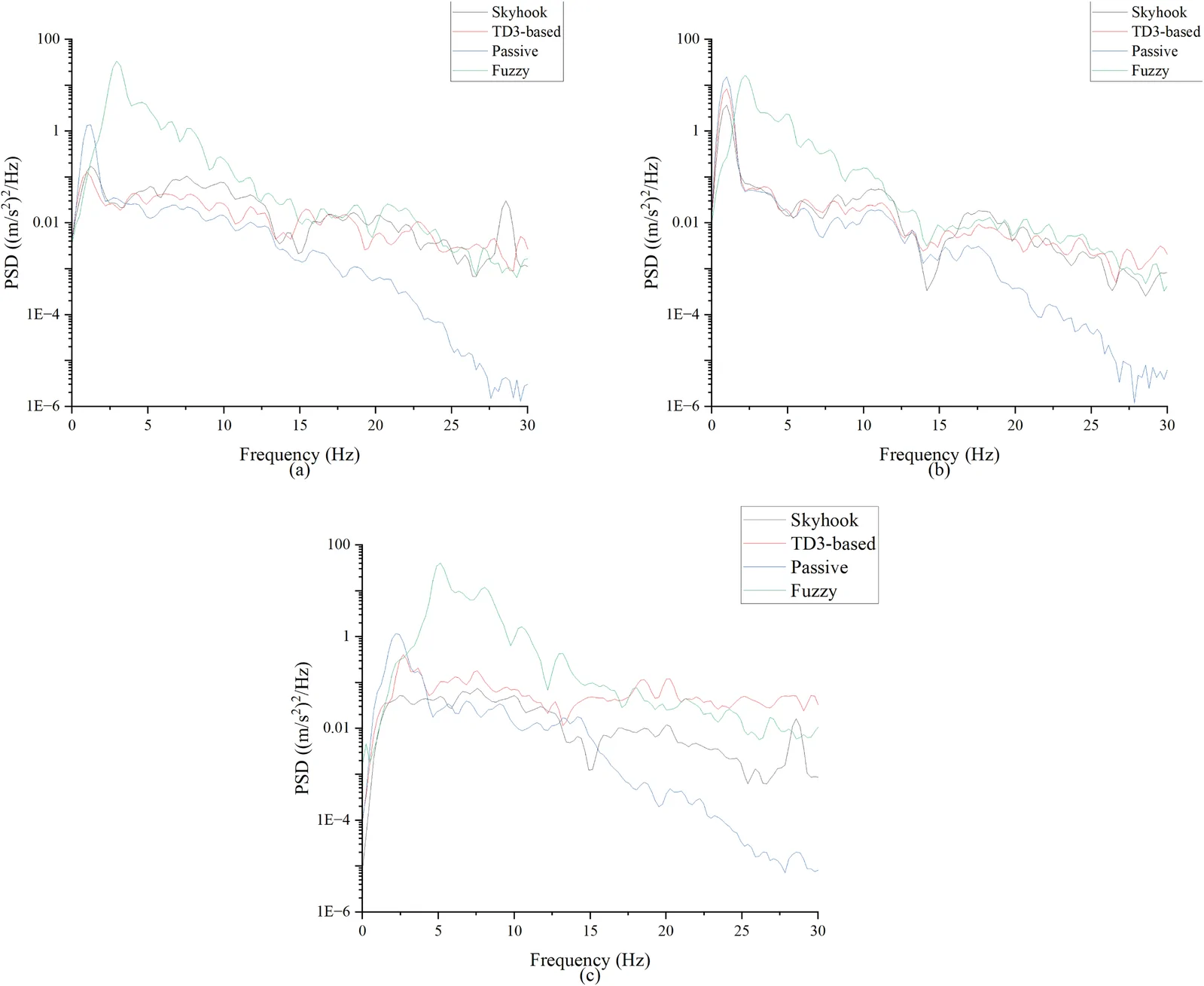
Power spectral density (PSD) of vehicle dynamic responses under Class A road excitation at 60 km/h: (a) Vertical acceleration; (b) Roll angular acceleration; (c) Pitch angular acceleration.

**Fig 15:**
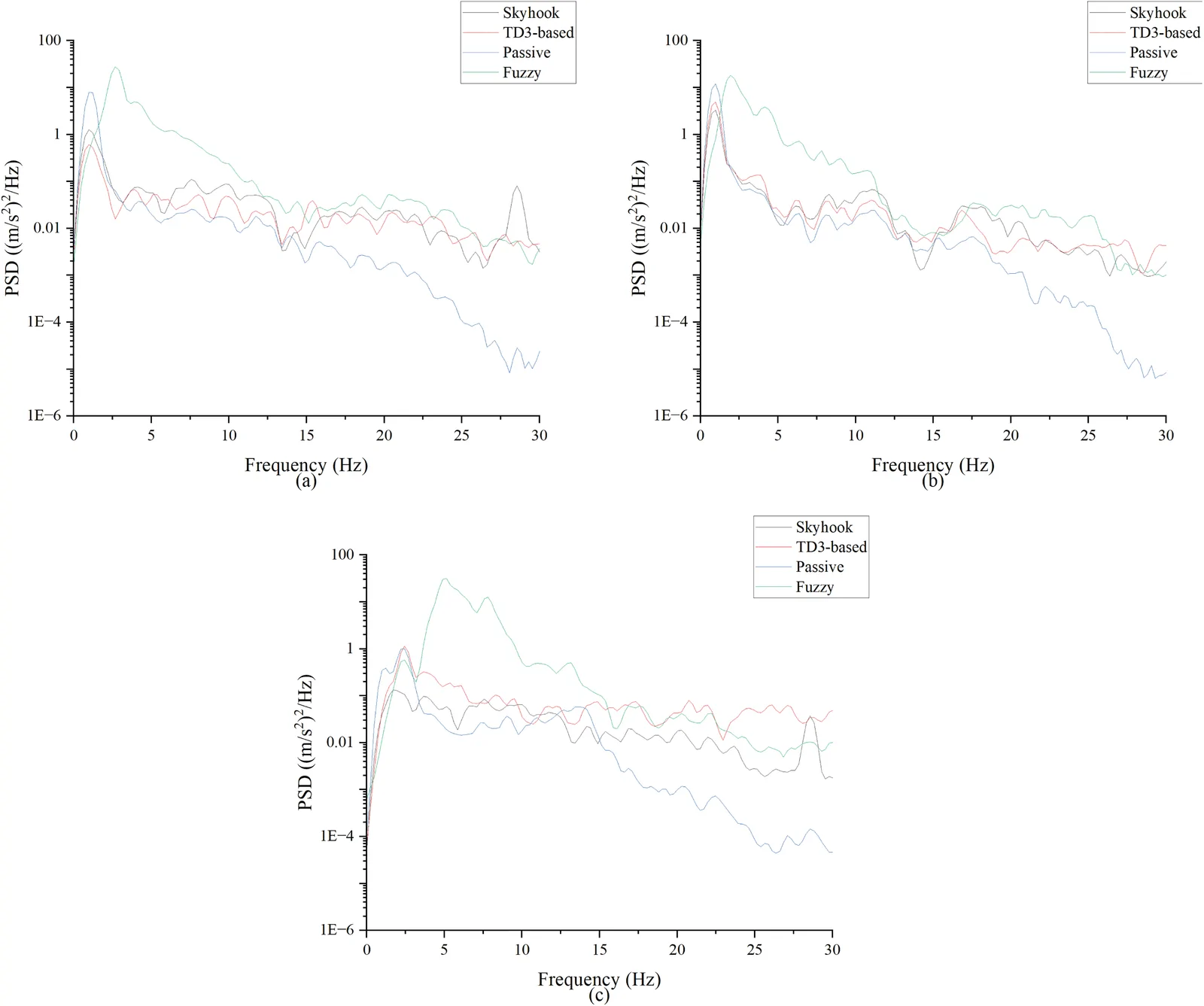
Power spectral density (PSD) of vehicle dynamic responses under Class A road excitation at 120 km/h: (a) Vertical acceleration; (b) Roll angular acceleration; (c) Pitch angular acceleration.

**Fig 16:**
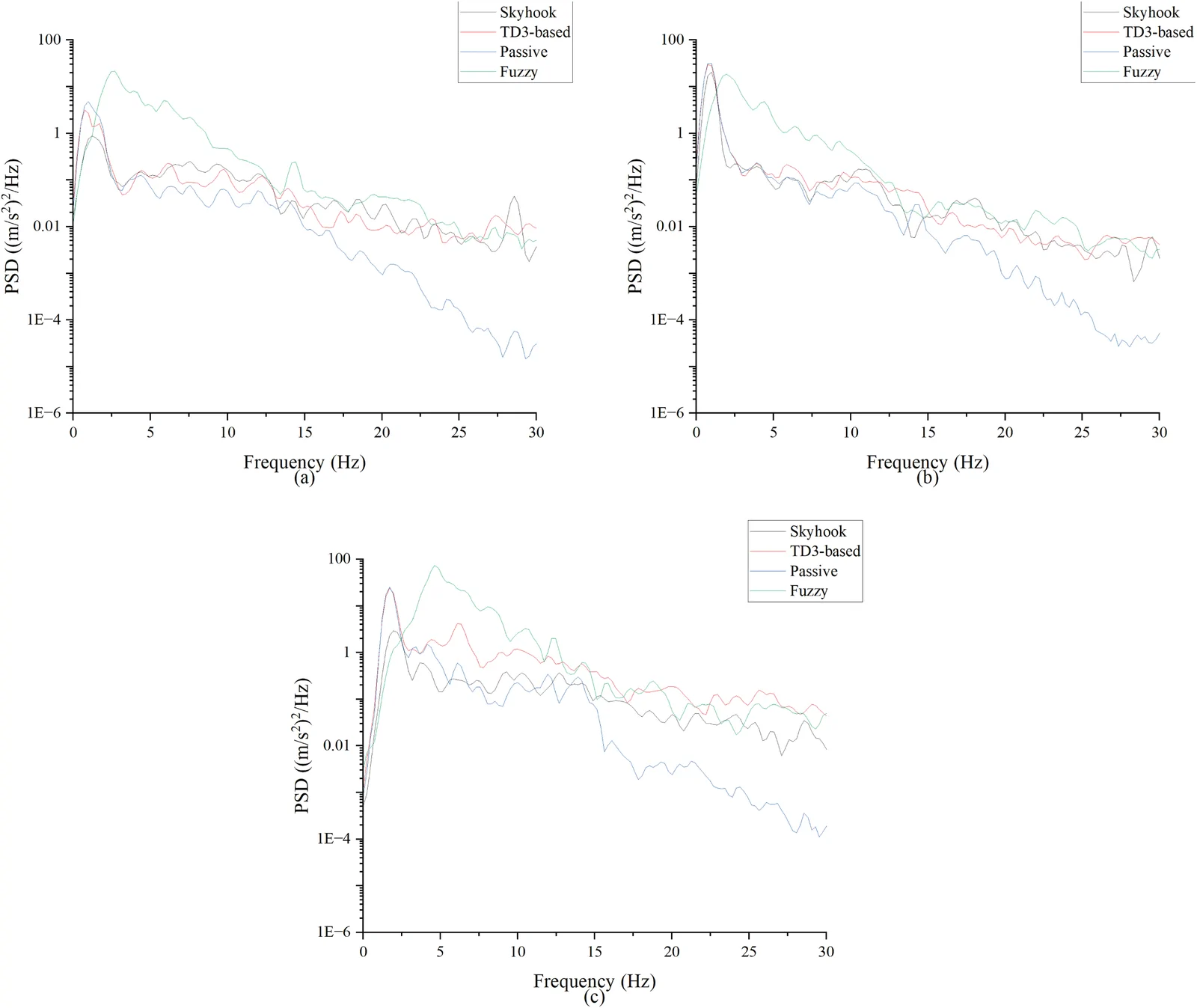
Power spectral density (PSD) of vehicle dynamic responses under Class B road excitation at 60 km/h: (a) Vertical acceleration; (b) Roll angular acceleration; (c) Pitch angular acceleration.

To further reveal the vibration isolation performance of different control strategies under realistic driving conditions, the power spectral density is introduced to analyze the dynamic responses of the vehicle system in the frequency domain. According to the ISO 2631 standard, the human body is most sensitive to vibrations at low frequencies, particularly in the range from 4 to 8 Hz for the vertical direction and 1–2 Hz for rotational directions. Furthermore, the natural frequency of the vehicle sprung mass typically concentrates within the 1–3 Hz range. Therefore, this section primarily evaluates the power spectral density curves of vertical acceleration, roll angular acceleration, and pitch angular acceleration within the frequency from 0 to 30 Hz. Figs 14, 15, and 16 illustrate the frequency responses of the vehicle under Class A at 60 km per h, Class A at 120 km per h, and Class B at 60 km per h road conditions respectively.

Under the Class A road excitation, a significant resonance peak at low frequencies emerges between 1 and 1.5 Hz for the passive suspension across both speed scenarios. Specifically, at 60 km per h, the passive suspension reaches a power spectral density peak of 1.36 ${\left(\text{m}/{\text{s}}^{\text{2}}\right)}^{\text{2}}/\text{Hz}$ at 1.22 Hz in the vertical direction. The proposed TD3 strategy effectively suppresses this peak to 0.126 ${\left(\text{m}/{\text{s}}^{\text{2}}\right)}^{\text{2}}/\text{Hz}$, outperforming the passive system, the classical Skyhook controller which has a peak of 0.17 ${\left(\text{m}/{\text{s}}^{\text{2}}\right)}^{\text{2}}/\text{Hz}$, and the Fuzzy method. Notably, the Fuzzy method exhibits a severe vertical resonance peak of 33.18 ${\left(\text{m}/{\text{s}}^{\text{2}}\right)}^{\text{2}}/\text{Hz}$ shifted to 2.93 Hz, indicating poor frequency domain performance. When the speed increases to 120 km per h, the intensified road excitation causes the vertical resonance peak of the passive suspension to surge to 7.918 ${\left(\text{m}/{\text{s}}^{\text{2}}\right)}^{\text{2}}/\text{Hz}$. In contrast, the TD3 strategy robustly limits the peak to 0.607 ${\left(\text{m}/{\text{s}}^{\text{2}}\right)}^{\text{2}}/\text{Hz}$, demonstrating strong adaptiveness to varying speeds, while the Fuzzy method again shows a massive peak of 27.64 ${\left(\text{m}/{\text{s}}^{\text{2}}\right)}^{\text{2}}/\text{Hz}$ at 2.69 Hz. Regarding rotational dynamics, both the TD3 and Skyhook methods substantially mitigate the resonance energy. For instance, at 60 km per h on the Class A road, the TD3 strategy reduces the roll angular acceleration peak from 15.26 ${\left(\text{rad}/{\text{s}}^{\text{2}}\right)}^{\text{2}}/\text{Hz}$ for the passive suspension to 8.32 ${\left(\text{rad}/{\text{s}}^{\text{2}}\right)}^{\text{2}}/\text{Hz}$, thereby enhancing the vehicle posture stability, whereas the Fuzzy method yields a peak of 16.63 ${\left(\text{rad}/{\text{s}}^{\text{2}}\right)}^{\text{2}}/\text{Hz}$ at 2.20 Hz.

Under the harsher Class B road excitation at 60 km per h, the severe road undulations at low frequencies induce intense body posture variations. The data reveals that the peaks for roll and pitch angular accelerations of the passive suspension soar to 31.83 ${\left(rad/s^{\text{2}}\right)}^{\text{2}}/Hz$ and 25.28 ${\left(rad/s^{\text{2}}\right)}^{\text{2}}/Hz$ respectively in the range of 0.7 to 1.7 Hz. The Fuzzy method exacerbates this instability, pushing the pitch peak to an extreme 73.39 ${\left(rad/s^{\text{2}}\right)}^{\text{2}}/Hz$ at 4.64 Hz and the vertical peak to 21.69 ${\left(m/s^{\text{2}}\right)}^{\text{2}}/Hz$ at 2.69 Hz. Under this condition, the TD3 controller maintains comprehensive control benefits. Although the specifically tuned Skyhook method exhibits strong forced damping characteristics in suppressing certain angular acceleration peaks, the proposed model independent data driven TD3 strategy intelligently finds a globally optimal balance between vertical vibration isolation and posture control without manual parameter tuning. This avoids the riding harshness often caused by excessive damping in a single degree of freedom.

Furthermore, observing the frequency band from 10 to 30 Hz across all conditions reveals that the power spectral density curves of the passive suspension generally remain at the lowest energy levels. Conversely, the high frequency values of the Skyhook and proposed TD3 strategies are slightly higher than that of the passive suspension. This represents a typical waterbed effect in semi active suspension control where substantially suppressing the massive energy in the low frequency resonance region causes the controller to rapidly adjust the damping force, inevitably transferring a minimal amount of energy to higher frequencies. However, since the absolute values of the vibration energy in this high frequency band are extremely small, and such high frequency chattering is easily filtered by the vehicle seats and rubber bushings, it does not negatively impact the subjective ride comfort of the passengers.

## 6. Conclusion

(1) Utilizing the TD3 algorithm, a TD3-based control method with good control effectiveness for a 7-DOF semi-active suspension model was proposed by altering the network model, setting training parameters, and defining the reward function. The 7-DOF vehicle semi-active suspension control method implemented with the TD3 algorithm, as proposed in this paper, exhibits advantages such as fast convergence speed and good control effectiveness.
(2) A TD3-based control method program, compatible with the entire vehicle suspension model, was developed using MATLAB software. This program enables numerical simulations of the 7-DOF semi-active suspension model under various road roughness using different control strategies.
(3) The article investigates the response of a vehicle suspension controller using the TD3-based control method on Class A road surfaces at speeds of 60 km/h and 120 km/h, as well as on Class B road surfaces at 60 km/h. The results indicate that the vehicle semiactive suspension controlled by the TD3 based method exhibits an average reduction of 19.76% to 49.41% in the vertical acceleration of the vehicle body compared to a passive suspension and 3.19% to 9.33% compared to the linear Skyhook control method in primary operating conditions. Furthermore the proposed deep reinforcement learning strategy consistently outperforms the traditional Fuzzy control method in suppressing low frequency multi axis vibrations without heavily relying on expert parameter tuning.
(4) Although the proposed TD3 based control method demonstrates excellent performance in vibration reduction and convergence the current study primarily focuses on the macroscopic dynamic response of the semi active suspension. The highly nonlinear hysteresis characteristics of the MFDs were simplified to prioritize the convergence of the deep reinforcement learning algorithm. In future research hardware in the loop testing will be conducted where actual physical MFDs will be integrated into the control loop to evaluate the robustness of the trained Actor network against unmodeled real world hysteresis. Additionally a multi objective reward function that explicitly balances ride comfort and energy consumption will be developed to further enhance the engineering applicability of the algorithm.
(5) The systematic evaluation of the suspension working space and dynamic tire load reveals an insightful control trade off. Under standard Class A road excitations the proposed TD3 based controller implicitly maintains these safety constraints within optimal bounds without requiring explicit penalty terms. However under harsher Class B road conditions the unconstrained DRL agent aggressively sacrifices tire force stability and suspension limits to prioritize ride comfort. This phenomenon validates the necessity of explicitly incorporating these safety constraints into a multi objective reward function which will be an essential step for the future physical deployment of DRL based active suspensions.
(6) The frequency domain analysis via power spectral density confirms that the TD3 strategy effectively mitigates the resonance energy of the sprung mass in the critical low frequency band from 0 to 30 Hz. Notably it robustly suppresses the fundamental resonance peaks around 1–2 Hz under both Class A and Class B road excitations without inducing high frequency chattering, outperforming the passive Skyhook and Fuzzy methods, thereby achieving a superior balance between vertical ride comfort and rotational posture stability.
