# Supplementary material for: A twin delayed deep deterministic-based control method for a full vehicle semi-active suspension system
Source: PLoS One. 2026 Sep 25;21(9):e0359102. doi: 10.1371/journal.pone.0359102 (PMC13614603; doi:10.1371/journal.pone.0359102)
Supplement: Supplementary file 1 [file pone.0359102.s002.docx]

List of revisions

**Paper No.:** PONE-D-26-32255

**Author(s):** Yongjun Wan, Xiaoming Wang, Gang Zhi, Lei Yang, Juye Wang, Xiangcheng Zhang

**Corresponding author:** Yan Zhang(sunshine9264kiki@outlook.com)

**Title:** A Twin Delayed Deep Deterministic-based control method for a full vehicle semi-active suspension system

Dear Academic Editor Jinhao Liang and Reviewers:

On behalf of all the contributing authors, I sincerely thank you for your letter and constructive comments on our paper. These comments are all valuable and very helpful for revising and improving our paper, as well as providing important guiding significance to our research. We have studied the comments carefully and made corrections. Point-by-point responses to the Editor and the Reviewers are listed below. The specific revision content is marked in red font in the Revised Manuscript. We are also pleased to submit this revised manuscript in accordance with the September 07 deadline.

**Responses to Journal Requirements (Academic Editor)**

1. Please ensure that your manuscript meets PLOS ONE's style requirements, including those for file naming.

**[Response]:** We sincerely thank the Editor for this reminder. We have carefully reviewed the provided PLOS ONE style templates and updated our manuscript formatting, headings, and file naming conventions accordingly to ensure full compliance.

2. Please note that PLOS One has specific guidelines on code sharing for submissions in which author-generated code underpins the findings in the manuscript. In these cases, we expect all author-generated code to be made available without restrictions upon publication of the work. Please review our guidelines at https://journals.plos.org/plosone/s/materials-and-software-sharing#loc-sharing-code and ensure that your code is shared in a way that follows best practice and facilitates reproducibility and reuse.

**[Response]:** We have reviewed PLOS ONE's guidelines on materials and software sharing. We confirm that all author-generated code underpinning our TD3 control method findings will be made fully available without restrictions upon the publication of this work to facilitate reproducibility and reuse.

3. Please amend the manuscript submission data (via Edit Submission) to include author Yongjun Wan, Xiaoming Wang, Lei Yang, Juye Wang and Xiangcheng Zhang.

**[Response]:** The manuscript submission data has been updated in the Editorial Manager system to correctly include all authors as requested.

4. Thank you for stating the following in the Competing Interests section: "The authors have declared that no competing interests exist." We note that one or more of the authors are employed by a commercial company: "China Construction Fifth Bureau (Henan) Construction Co., Ltd."

(1) Please provide an amended Funding Statement declaring this commercial affiliation, as well as a statement regarding the Role of Funders in your study... Please also include the following statement within your amended Funding Statement: "The funder provided support in the form of salaries for authors [insert relevant initials], but did not have any additional role..."

**[Response]:** Thank you for pointing this out. As requested, we have included an amended Funding Statement in our cover letter. We note your instruction that the journal office will change the online submission form on our behalf. For your convenience, our updated Funding Statement is provided below. It clarifies that the transportation bureau funded the entire project, while the commercial affiliation provided salary support:

*This research was financially supported by the Science and Technology Project of Henan Provincial Department of Transportation under grant number 2020J-2-9, which funded the entire research project. Additionally, the commercial affiliation, China Construction Fifth Bureau (Henan) Construction Co., Ltd., provided support in the form of salaries for author(s) (Xiaoming Wang and Lei Yang), but did not have any additional role in the study design, data collection and analysis, decision to publish, or preparation of the manuscript. The specific roles of these authors are articulated in the 'author contributions' section.*

(2) Please also provide an updated Competing Interests Statement declaring this commercial affiliation... Within your Competing Interests Statement, please confirm that this commercial affiliation does not alter your adherence to all PLOS ONE policies...

**[Response]:** We have also included an updated Competing Interests Statement in our cover letter. Our updated Competing Interests Statement is provided below, which includes the required adherence statement:

*One or more of the authors are employed by a commercial company: China Construction Fifth Bureau (Henan) Construction Co., Ltd. This does not alter our adherence to PLOS ONE policies on sharing data and materials.*

5. Please include a new copy of Table 3 in your manuscript; the current table is difficult to read.

**[Response]:** A new, high-resolution, and clearly formatted copy of Table 3 has been included in the revised manuscript. See in lines 466.

**Table 3. TD3 controller of vehicle 7-DOF suspension model.**

**Algorithm 1** TD3-based control method under vehicle 7-DOF semi-active suspension model

Initialize suspension model with parameter.

Initialize critic networks,, and actor network with random parameters,,.

Initialize target networks,, with weight,,.

Initialize replay buffer

**for** t = (0, step, T) **do**

Determine training parameters doTrain and doDelayUpdate based on the current iteration.

Determine the current values of road surface excitation based on the road roughness model.

,,,

Calculate Fci using the formula.

update, using

**if** doTrain = 1 **then**

update s using,,

Store state as, store reward as, store transition tuple in

Sample mini-batch from
,

Update critics

**if** doDelayUpdate = 1 **then**

Update by

Update target networks

**end if**

Select next action.

Store,.

Calculate output current.

**end if**

**end for**

6. If the reviewer comments include a recommendation to cite specific previously published works, please review and evaluate these publications to determine whether they are relevant and should be cited.

**[Response]:** We have carefully evaluated all publications recommended by the reviewers, found them highly relevant to our research on intelligent vehicle suspension control, and incorporated them into our enriched literature review.

**Responses to Reviewer #1**

1. Abstract: The abbreviation definitions can be moved to the body text.

**[Response]:** We sincerely thank the Reviewer for this suggestion. We have removed the abbreviation definitions from the abstract, using their full terminology instead (e.g., Twin Delayed Deep Deterministic, deep reinforcement learning, 7-degree-of-freedom, root mean square). Meanwhile, we have ensured that these abbreviations are properly defined at their first appearance in the body text. See in lines 2-28.

*“To improve the convergence issues of deep reinforcement learning in the control of vehicle semi-active suspension, and enhance the control effectiveness of the vehicle's semi-active suspension, a Twin Delayed Deep Deterministic-based control framework tailored for the high-dimensional nonlinear dynamics of the vehicle semi-active suspension model is proposed. Rather than merely applying standard deep reinforcement learning, this study introduces a domain-specific state-action mapping mechanism and a multi-objective reward function to physically constrain the neural network outputs and coordinate the full-vehicle dynamic responses. The simulation program for 7-degree-of-freedom vehicle semi-active suspension model embedded in the proposed control method is developed using MATLAB software. The validity of the proposed control method is verified using the developed program under different road roughness and speeds. The results indicate that compared to the passive suspension the classical linear Skyhook control method and the Fuzzy control method the proposed Twin Delayed Deep Deterministic based method achieves superior control effectiveness. Specifically in terms of the vehicle body vertical acceleration the proposed method achieves a reduction of 49.41 % compared to the passive suspension and 9.33 % compared to the Skyhook method on a Class A road at 60 km/h a reduction of 24.82 % compared to the passive suspension on a Class A road at 120 km/h and a reduction of 3.19 % compared to the Skyhook method on a Class B road at 60 km/h. Furthermore compared to the traditional Fuzzy control method the proposed method demonstrates superior vibration suppression effectiveness across all evaluated operating conditions achieving significantly lower frequency weighted vertical root mean square accelerations. Furthermore the power spectral density analysis demonstrates that the proposed method significantly suppresses the low frequency resonance of the sprung mass across varying road conditions compared to the passive Skyhook and Fuzzy methods, further validating its superior broadband vibration isolation capability. This demonstrates that the Twin Delayed Deep Deterministic-based control method for the vehicle semi-active suspension model has the advantages of fast convergence speed and good control effectiveness under road surface excitation.”*

2. Mention the quantitative improvements more clearly and compare with which recent sophisticated methods, not just the classical Skyhook.

**[Response]:** We sincerely appreciate the reviewer for this constructive suggestion regarding the quantitative improvements and baseline comparisons. In the revised manuscript we have updated the abstract and the simulation results section to explicitly present the specific performance enhancements under various road conditions and speeds. To demonstrate the advanced nature of our proposed approach beyond the classical Skyhook method we have incorporated the traditional Fuzzy control method as an additional sophisticated baseline. The updated text now clearly states that on a Class A road at 60 kilometers per hour the proposed method achieves a reduction of 49.41% in vertical acceleration compared to the passive suspension and 9.33% compared to the Skyhook method. Furthermore the revised manuscript explicitly demonstrates that our proposed strategy consistently outperforms the Fuzzy control method by achieving significantly lower frequency weighted vertical root mean square accelerations across all evaluated operating conditions. We believe these detailed quantitative additions and the inclusion of the Fuzzy control baseline fully address your concern and significantly strengthen the manuscript for final evaluation. See in lines 14-22 and 164-166.

*“Specifically in terms of the vehicle body vertical acceleration the proposed method achieves a reduction of 49.41 % compared to the passive suspension and 9.33 % compared to the Skyhook method on a Class A road at 60 km/h a reduction of 24.82 % compared to the passive suspension on a Class A road at 120 km/h and a reduction of 3.19 % compared to the Skyhook method on a Class B road at 60 km/h. Furthermore compared to the traditional Fuzzy control method the proposed method demonstrates superior vibration suppression effectiveness across all evaluated operating conditions achieving significantly lower frequency weighted vertical root mean square accelerations.”*

*“The results of the semi-active suspension controlled by the TD3-based control method are compared with those of passive suspension, the classical linear Skyhook control method, and the traditional Fuzzy control method.”*

3. Avoid group referencing.

**[Response]:** We have carefully reviewed the manuscript and eliminated group referencing, ensuring each cited work is discussed individually to provide a clearer literature context. See in lines 31-181.

*“During vehicle driving, the motion relationship between the vehicle and the road surface usually includes three directions: transverse, longitudinal, and vertical. When affected by changes in the road surface, the vehicle body moves up and down. When driving or riding in a vehicle, people expect to feel the effects of as little vertical excitation as possible while not affecting driving handling. Therefore, scholars typically focus on the vehicle's response to road surface excitation when discussing vertical dynamics. The vehicle suspension supports the body during driving, isolates road surface excitation, and provides driving comfort. Using the suspension to fix the body to the axle, the body can be isolated from the axle by the springs and dampers of the suspension, thereby reducing the impact of the body on the roughness of the road. Generally, the suspension design problem can be simplified to the coupling problem of spring and damper in the vehicle suspension design process [1]. For example, in a typical quarter suspension model, suspension design problems can be reduced to a 3-DOF system involving pavement, sprung mass, and unsprung mass [2].*

*In terms of controllability, vehicle suspensions are generally classified into passive suspension, semi-active suspension, and active suspension. Semi-active suspension is widely used in new passenger cars because of its lower cost and better control effect. In recent years, there also have been some developments in its design [3] and operational principles [4]. Studies have shown that the performance of semi-active dampers can closely approach that of fully active systems in balancing ride comfort and road safety [5]. To further enhance control effectiveness, advanced force-tracking approaches integrating semi-active dampers with semi-active inerters have been proposed [7]. Among the various damping devices developed, the magnetorheological fluid damper (MFD) is one of the most widely used in recent years [8], and there has been considerable research on the design methods [9] and mechanical models [10] of such dampers. The MFD, which has been successfully designed and validated even for full-scale railway vehicle suspensions to improve ride quality [6], changes the dynamic viscosity of the magnetorheological fluid in the damper at different currents, thus producing different damping forces [11], and enables various advanced control adaptations [12]. MFDs have the advantages of fast response, mature technology, and a large damping adjustment range. The response speed of magnetorheological fluids under changes in magnetic fields can reach the millisecond level [13], which is one of the reasons for the fast response speed of MFDs [14]. Not only in passenger vehicle suspension control, but MFDs are also widely applied in other engineering domains. For instance, in building structures, MR dampers are utilized to mitigate earthquake responses [15], and combined with neural network-based intelligent control strategies to resolve time-delay issues [16]. Furthermore, for high-speed rail vehicles, semi-active suspensions equipped with MR dampers and predictive model controllers have been shown to significantly improve ride quality while maintaining running safety [17]. These research efforts provide significant inspiration for the study presented in this paper on the semi-active control of vehicle suspension.*

*The vehicle suspension model serves to validate the damping effects of the vehicle's suspension system. A model typically yields simulation results close to those observed in the suspension's actual operation [18]. For individual axle-based suspension studies, employing a quarter suspension model proves effective [19]. However, due to considerations of the realism of vehicle suspension, such as excitation characteristics between front and rear wheels, suspension layout, and collecting vehicle angle information, utilizing half or full-vehicle models becomes necessary [20]. Furthermore recent studies emphasize the critical importance of integrated chassis control in vehicle models to meet modern performance demands. For example to simultaneously enhance longitudinal dynamics and vertical ride comfort advanced frameworks based on multi-agent systems have been proposed to integrate active suspension systems with torque vectoring [45]. Similarly model predictive controllers have been developed for such integrated systems to optimize energy saving and safety under linear models [46]. Driven by the necessity of such comprehensive dynamics modeling and multi-axis coordination this paper employs a seven-degree-of-freedom vehicle suspension model, which is a full vehicle model, to thoroughly investigate suspension control effects.*

*In research on semi-active suspension control strategies, traditional control methods such as the skyhook control method serve as baseline comparisons due to their simple and practical control algorithms, good control effects, and lower development difficulty [21]. In particular, the linear sky-hook control method, when applied to a semi-active suspension system with MFD, significantly enhances suspension performance by employing linear functions to control the current of dampers. However, the skyhook control method typically applies only to quarter suspension models, often lacking optimal solutions for entire vehicle control. Linear Quadratic Regulator (LQR) controllers are also frequently used in semi-active suspension control. Yet, due to challenges in determining weighted matrices for semi-active suspensions [22], and optimizing the control parameters under complex constraints [23], the development of these controllers poses significant difficulties. Addressing these issues, employing machine-learning and data-driven control methods significantly reduces the complexity of control strategy development. For instance, to tackle the severe nonlinearity and rate-dependent hysteresis of magneto-rheological devices without relying on complex analytical models, data-driven sliding mode control approaches have been successfully implemented [42]. Such intelligent methods show promising control effects even on entire vehicle models [24]. However, particularly for advanced machine learning branches like deep reinforcement learning (DRL) algorithms, the control effects often rely heavily on the gradient direction of convergence. Therefore, establishing models scientifically remains a key focus in these data-driven methods.*

*Deep reinforcement learning is a machine learning decision-making method that has attracted more attention in recent years [25]. Usually, a reinforcement learning (RL) algorithm uses a value function to judge the value of behavior [26], which is typically modeled within the framework of a Markov chain [27]. By using a neural network model to establish a value function, the established DRL algorithm can more easily achieve gradient descent. Unlike other machine learning methods, DRL, which relies on the learning process of actions based on environmental variables, is widely applied in fields such as production and daily life, where machine autonomy is crucial. Examples like AlphaGo have garnered significant attention among scholars [28]. In recent years, there have been attempts to apply DRL to production processes [29].*

*Within the research on DRL strategies, two commonly used structures are the value Q-function method [30] and the Actor-critic method [31]. The DQN algorithm utilizes a Q-value network to obtain Q-values for several actions and selects the best action, commonly applied to discrete action spaces. On the other hand, the DDPG algorithm employs an actor network and a critic network. It optimizes the critic network by evaluating the values of actions in the environment and then optimizes the actor network based on the critic network's evaluations. This method is suitable for continuous action spaces. DDPG-based algorithms have demonstrated robust decision-making capabilities in complex vehicle dynamics, such as human-machine shared steering control during emergency obstacle avoidance [43]. However, DRL algorithms often face challenges regarding sample efficiency and Q-value estimation errors. To address this, recent studies have developed backtrack temporal difference learning methods to enhance target Q-value estimation precision [44]. Building on the need to improve convergence, in 2018, Fujimoto et al. introduced the Twin Delayed Deep Deterministic (TD3) algorithm [32], which uses two critic networks to enhance the convergence process. TD3 improves upon the DDPG algorithm, enhancing the convergence of the Actor-Critic method.*

*In recent years, driven by the rapid trends of vehicle electrification [33] and intelligent autonomous driving [34], active and semi-active suspension technologies have experienced unprecedented development to meet increasingly stringent demands for ride comfort and integrated chassis control. While the performance of traditional controllers like Skyhook is strongly influenced by hardware constraints, such as the response time of magnetorheological valves [35], DRL control algorithms using the Actor-Critic structure (such as DDPG) can often achieve more robust and adaptable control. For instance, recent studies have successfully deployed Soft Actor-Critic (SAC) models in real vehicles, enabling the suspension system to dynamically adapt to varying real-road disturbance profiles [36]. In the realm of semi-active suspensions, scholars have extensively validated the efficacy of the Actor-Critic method (such as the DDPG algorithm) in controlling the vehicle's quarter suspension model [24]. Additionally, there is research exploring the combination of DRL methods with traditional control approaches to achieve control in a relatively stable manner [37]. However, there has been a slight scarcity of research regarding DRL in the context of full-vehicle suspension models. Through testing, the author suggests this might be related to the convergence issues of DDPG, as at times, using the DDPG algorithm can make it difficult for Q-value predictions to converge to a consistently stable level.*

*To address the issues of slow convergence speed and relatively low convergence in the control of semi-active suspension with the DDPG algorithm under the full vehicle suspension model, and to enhance the effectiveness of the controller, this paper first introduces a 7-DOF semi-active suspension model for vehicles and a road surface model established using the PSD specified by ISO 8608. Specifically, the fundamental improvements of the proposed TD3 framework over conventional DRL methods lie in two aspects. First, unlike the DDPG algorithm which suffers from severe Q-value overestimation in the highly coupled 7-DOF full-vehicle environment, the integrated TD3 architecture employs twin delayed critics to ensure stable policy evaluation under complex multi-axis road excitations. Second, a customized state-action mapping and a physically meaningful reward function are designed to bridge the gap between unconstrained DRL algorithms and the strict physical boundaries of magnetorheological fluid dampers (MFDs), ensuring that the generated control currents are both theoretically optimal and physically executable. Based on the TD3 algorithm, a TD3-based control method is proposed for the current of the semi-active control device in the vehicle suspension. This is achieved through the redefinition and validation of the neural network model, training parameters, and reward functions in DRL. In the control method, the state is updated based on the 7-DOF semi-active suspension model for vehicles. Using MATLAB software, this paper constructs a 7-DOF semi-active suspension model for vehicles, develops a program of the TD3-based control method for vehicle suspension, and validates the proposed controller under varying road roughness and speeds. The results of the semi-active suspension controlled by the TD3-based control method are compared with those of passive suspension, the classical linear Skyhook control method, and the traditional Fuzzy control method. The effectiveness and convergence of the TD3-based control method are validated through this comparison. Furthermore recent studies emphasize the importance of advanced integrated chassis control frameworks. For instance sophisticated methodologies like fuzzy game theoretic tube model predictive control have been developed to ensure robust constraint satisfaction and coordinate heterogeneous actuators for integrated vehicle lateral stability [49]. Inspired by the application of fuzzy logic in advanced vehicle control and to thoroughly validate the proposed TD3 algorithm this paper employs a sophisticated Fuzzy Logic Control algorithm as an advanced benchmark alongside the traditional Skyhook method.*

*The remainder of this paper is organized as follows. Section 2 establishes the 7-DOF vehicle semi-active suspension model and derives its dynamic equations. Section 3 introduces the road surface roughness model based on the ISO 8608 standard. Section 4 details the design of the TD3-based semi-active suspension controller, including the definitions of the state space, action space, and reward function. Section 5 presents the numerical simulation results and discussions, evaluating the proposed control method against baseline methods under different road classifications and vehicle speeds. Finally, the conclusions are drawn in Section 6.”*

4. The literature review needs to be enriched by some recent research works... Similar data-driven and advanced control approaches for magnetorheological systems are discussed in (DOI: 10.1109/TCYB.2025.3596063).

**[Response]:** We have enriched the literature review with recent research on data-driven and advanced control approaches for magnetorheological systems, incorporating the suggested reference (DOI: 10.1109/TCYB.2025.3596063). See in lines 94-102.

*“Addressing these issues, employing machine-learning and data-driven control methods significantly reduces the complexity of control strategy development. For instance, to tackle the severe nonlinearity and rate-dependent hysteresis of magneto-rheological devices without relying on complex analytical models, data-driven sliding mode control approaches have been successfully implemented [42]. Such intelligent methods show promising control effects even on entire vehicle models [24]. However, particularly for advanced machine learning branches like deep reinforcement learning (DRL) algorithms, the control effects often rely heavily on the gradient direction of convergence. Therefore, establishing models scientifically remains a key focus in these data-driven methods.”*

5. For efficient deep reinforcement learning frameworks in vehicle control, similar approaches could be strengthened by referring to (DOI: 10.1109/TVT.2025.3629701) and (DOI: 10.1016/j.knosys.2025.114613).

**[Response]:** We have strengthened our discussion on efficient deep reinforcement learning frameworks in vehicle control by referring to and discussing the recommended literatures (DOI: 10.1109/TVT.2025.3629701 and DOI: 10.1016/j.knosys.2025.114613). See in lines 119-127.

*“DDPG-based algorithms have demonstrated robust decision-making capabilities in complex vehicle dynamics, such as human-machine shared steering control during emergency obstacle avoidance [43]. However, DRL algorithms often face challenges regarding sample efficiency and Q-value estimation errors. To address this, recent studies have developed backtrack temporal difference learning methods to enhance target Q-value estimation precision [44]. Building on the need to improve convergence, in 2018, Fujimoto et al. introduced the Twin Delayed Deep Deterministic (TD3) algorithm [32], which uses two critic networks to enhance the convergence process. TD3 improves upon the DDPG algorithm, enhancing the convergence of the Actor-Critic method.”*

6. Add a paragraph to introduce the paper organisation at the end of the Introduction.

**[Response]:** A new paragraph outlining the organization of the remainder of the paper has been added at the end of the Introduction. See in lines 175-181.

*“The remainder of this paper is organized as follows. Section 2 establishes the 7-DOF vehicle semi-active suspension model and derives its dynamic equations. Section 3 introduces the road surface roughness model based on the ISO 8608 standard. Section 4 details the design of the TD3-based semi-active suspension controller, including the definitions of the state space, action space, and reward function. Section 5 presents the numerical simulation results and discussions, evaluating the proposed control method against baseline methods under different road classifications and vehicle speeds. Finally, the conclusions are drawn in Section 6.”*

7. "Table 4. Parameters of vehicle suspension model." How do you determine the uncertainties? Lack of physical discussion about the parameters.

**[Response]:** We sincerely thank the reviewer for the constructive feedback regarding the parameter uncertainties and physical discussion. To address this concern we have updated Table 4 to explicitly quantify the uncertainty boundaries for the sprung mass and the rolling and pitching moments of inertia. Furthermore we have expanded Section 5.2 to include a detailed physical discussion explaining that these uncertainties originate from realistic physical environments specifically the variations in passenger loading within a standard automobile. By calculating the maximum mass fluctuation based on an average passenger weight we established robust physical boundaries for our seven degrees of freedom suspension model. We believe these revisions clarify the physical rationale behind our parameter selection and fully address the concerns raised. See in lines 502-512 and 538.

*“The parameters for the vehicle model are determined as shown in Table 4. In realistic physical environments, vehicle parameters are inevitably subjected to uncertainties, which primarily originate from changes in passenger loading [38]. For instance, assuming a conservative average passenger weight of approximately 80 kg within a four-seat vehicle configuration, the variation of the sprung mass can reach up to 320 kg [38]. Consequently, the parameter variations in this study are fundamentally determined based on these sprung mass fluctuations and their corresponding impact on the moments of inertia [38]. The specific parameter boundaries for the sprung mass and the inertia variations are explicitly defined in Table 4 to guarantee the robustness of the physical model under realistic operating conditions. Furthermore, the TD3 algorithm in the simulation includes the critic network and actor networks presented in Tables 5 and 6.”*

***Table 4. Parameters of vehicle suspension model.****[38]*

| ***Parameter*** | ***Value*** | ***Uncertainty(***$\Delta$***)*** | ***Unit*** | ***Parameter*** | ***Value*** | ***Uncertainty(***$\Delta$***)*** | ***Unit*** |
| --- | --- | --- | --- | --- | --- | --- | --- |
| $k_{s}$ | *20000* | *0* | *N/m* | $m_{s}$ | *1500* | *165* | *kg* |
| $k_{t}$ | *200000* | *0* | *N/m* | $m_{u}$ | *28* | *N/A* | *kg* |
| $k_{r}$ | *10000* | *0* | *N/m* | $I_{y}$ | *1423.8* | *149.16* | *kg·m^2^* |
| $b_{1},b_{2}$ | *0.71* | *N/A* | *m* | $I_{z}$ | *504* | *52.8* | *kg·m^2^* |
| $a_{1}$ | *0.91* | *N/A* | *m* | $g$ | *9.8* | *N/A* | *m/s^2^* |
| $a_{2}$ | *1.44* | *N/A* | *m* |  |  |  |  |

8. How can the control design be generalized for the other cases?

**[Response]:** We sincerely thank the reviewer for raising this important question regarding the generalization of the control design. As added to the revised manuscript the TD3 control method has lower generalization ability than skyhook damping control but higher than fuzzy PID control. Because road excitations generally remain within the range specified by ISO8608 the variations in suspension output such as suspension velocity and acceleration are limited across different vehicles. Therefore by simply adjusting the reward function a reasonable TD3 controller can be obtained enabling the transfer of the controller from one suspension set to another. We have preliminarily verified this generalization ability by showing that when driving speed and road grade change the same training results can produce similar outcomes and the output damping force remains consistently within the normal range without showing prolonged periods of excessively high or low damping. Thus using the TD3 algorithm to design a controller in a suspension model does not lead to significant overfitting under reasonable parameters. Through multiple training sessions we consider that approximately 30000 seconds of training is sufficient to obtain relatively reasonable controller results under the same speed condition and for suspension variations occurring over time due to usage retraining can be performed to obtain more reasonable parameters ensuring the reliability of passenger cars throughout their operational cycle. See in lines 280-296.

*“For suspension control, the TD3 control method has lower generalization ability than skyhook damping control but higher than fuzzy PID control. For different suspensions of vehicles, because road excitations generally remain within the range specified by ISO8608, the variations in suspension output, such as suspension velocity and acceleration, are limited across different vehicles. Therefore, by simply adjusting the reward function, a reasonable TD3 controller can be obtained, enabling the transfer of the controller from one suspension set to another. At the same time, the generalization ability of the TD3 controller results has been preliminarily verified: when driving speed and road grade change, the same training results can produce similar outcomes, and the output damping force remains consistently within the normal range without showing prolonged periods of excessively high or low damping. Thus, it can be considered that using the TD3 algorithm to design a controller in a suspension model does not lead to significant overfitting under reasonable parameters. Through multiple training sessions, it is considered that under the same speed condition, approximately 30,000 seconds of training is sufficient to obtain relatively reasonable controller results. For suspension variations occurring over time due to usage, retraining can be performed to obtain more reasonable controller parameters, thereby ensuring the reliability of passenger cars throughout their entire operational cycle.”*

9. “The hysteresis characteristics of the MFD were neglected...” Why? This is a major simplification. More discussions are needed about the MFD model and validation method.

**[Response]:** We acknowledge this simplification and have added a detailed justification explaining why the MFD hysteresis characteristics were neglected in this specific study. See in lines 473-485 and 814-822.

*“The hysteresis characteristics of the MFD were neglected in this study to reduce computational complexity. It is acknowledged that real world MFDs exhibit strong rate dependent hysteresis and omitting this is a simplification. However incorporating complex differential equation based hysteretic models like the Bouc Wen model into a highly coupled 7 DOF full vehicle environment would exponentially increase the computational burden and potentially destabilize the gradient convergence of the deep reinforcement learning training process. Since the primary focus of this study is the multi objective posture coordination of the full vehicle dynamics namely minimizing vertical roll and pitch accelerations and the validation of the continuous state action mapping mechanism the hyperbolic tangent approximation is deemed an appropriate compromise. It effectively captures the primary physical bounds specifically maximum and minimum damping forces and the macroscopic nonlinear saturation characteristics of the MFD which provides a sufficient dynamic baseline for evaluating the global control policy of the TD3 agent at this theoretical stage.”*

*“(4) Although the proposed TD3 based control method demonstrates excellent performance in vibration reduction and convergence the current study primarily focuses on the macroscopic dynamic response of the semi active suspension. The highly nonlinear hysteresis characteristics of the MFDs were simplified to prioritize the convergence of the deep reinforcement learning algorithm. In future research hardware in the loop testing will be conducted where actual physical MFDs will be integrated into the control loop to evaluate the robustness of the trained Actor network against unmodeled real world hysteresis. Additionally a multi objective reward function that explicitly balances ride comfort and energy consumption will be developed to further enhance the engineering applicability of the algorithm.”*

10. “The reward function R(s)...” What's the meaning of the weights? How do you determine them? Your design process seems very simple, like a course homework!

**[Response]:** We sincerely thank the reviewer for the rigorous evaluation of our reward function design. As we have now elaborated in the revised manuscript the weights were determined through extensive testing rather than a simple arbitrary selection. The reward function employs a weighted sum of the vertical acceleration and vertical velocity at the transformed vehicle center of mass to achieve relatively effective control performance. During our testing phase we observed that while angular velocity angular acceleration vertical velocity acceleration and vibration frequency are all key parameters for damping our primary concern is vertical acceleration and frequency. Extensive testing shows that using angular velocity or angular acceleration causes the TD3 control method to favor obtaining more rewards for those parameters and setting a reward cap provides little benefit. Conversely when vertical acceleration alone is used as the reward function input the control algorithm tends to prioritize higher rewards at the expense of controlling the suspension deformation direction resulting in uncontrollable vertical displacement changes under control. Therefore introducing velocity weighting not only keeps the suspension motion range within a controllable scope but also implicitly adjusts the suspension vibration frequency to a range less sensitive to human perception. Because frequency inputs need to be considered holistically in the frequency domain which leads to more complex policy outcomes that are difficult to derive quickly we concluded that our approach of using acceleration weighting to reduce transmitted vibrations and velocity weighting to control the motion range provides a highly effective and physically meaningful solution. See in lines 396-414.

*“The reward function has been tested and employs a weighted sum of the vertical acceleration and vertical velocity at the transformed vehicle center of mass, achieving relatively effective control performance: in the vehicle suspension model, angular velocity, angular acceleration, vertical velocity, acceleration, and vibration frequency at the vehicle center of mass are key parameters for damping, while our primary concern is vertical acceleration and frequency. Extensive testing shows that using angular velocity/angular acceleration causes the TD3 control method to favor obtaining more rewards for angular velocity/angular acceleration, and setting a reward cap provides little benefit. When vertical acceleration alone is used as the reward function input, the control algorithm tends to prioritize higher rewards at the expense of controlling the suspension's deformation direction, resulting in uncontrollable vertical displacement changes under control. Therefore, introducing velocity weighting not only keeps the suspension's motion range within a controllable scope but, as tests indicate, a reasonable velocity weight can adjust the suspension's vibration frequency to a range less sensitive to human perception. For the reward function in the TD3 controller, frequency inputs need to be considered holistically in the frequency domain, which leads to more complex policy outcomes that are difficult to derive quickly. Thus, acceleration weighting can reduce the vibrations transmitted from the suspension to the vehicle body, while velocity weighting can control the suspension's motion range and implicitly regulate the vibration frequency transmitted to the vehicle body.”*

11. How do you evaluate the ride comfort with respect to the ISO 2631 standard criterion and frequency-based calculation?

**[Response]:** We sincerely thank the reviewer for this insightful question regarding the evaluation of ride comfort. To rigorously assess passenger ride comfort in accordance with the ISO 2631 standard, we incorporated frequency weighted root mean square acceleration calculations into our evaluation framework. Recognizing that the human body exhibits varying sensitivity to vibrations across different frequencies, we applied the standard frequency weighting curves. For the vertical ride comfort evaluation, the sprung mass vertical acceleration was processed using the standard vertical weighting filter, and to ensure efficient continuous time domain simulation, a high precision third order approximation filter was adopted. For rotational dynamics affecting ride comfort, the roll and pitch angular accelerations were similarly evaluated using the corresponding angular weighting networks constructed based on the exact parameters specified in the standard. The frequency weighted root mean square accelerations were computed over the entire simulation time horizon under all tested operating conditions. These calculated metrics provide a standardized and quantifiable benchmark for evaluating human vibration exposure. To make this evaluation transparent, we have updated the revised manuscript to explicitly describe the mathematical implementation of the ISO 2631 weighting filters and added a comprehensive summary table that presents the frequency weighted root mean square values for vertical, roll, and pitch directions under all operating conditions, allowing direct comparison among the passive suspension, classical Skyhook control, and our proposed method. See in lines 10-14, 547-559, 566-568, 620-626, 647-649 and 669-683.

*“The validity of the proposed control method is verified using the developed program under different road roughness and speeds. The results indicate that compared to the passive suspension the classical linear Skyhook control method and the Fuzzy control method the proposed Twin Delayed Deep Deterministic based method achieves superior control effectiveness.”*

*“When assessing passenger comfort, considering that the human body is most sensitive to the vertical accelerations experienced, our priority is to reduce the vertical accelerations at the vehicle's center of gravity. Simultaneously, minimizing the angular accelerations at the vehicle's center of gravity is also desired. To accurately evaluate this ride comfort, the frequency-weighted root mean square (RMS) acceleration is calculated in accordance with the ISO 2631-1:1997 standard and its 2010 amendment [47]. Specifically, the vertical acceleration of the sprung mass is evaluated using the* $W_{k}$*frequency weighting filter. To facilitate efficient continuous-time domain simulation, a highly accurate third-order approximation of the* $W_{k}$*weighting filter [48] was adopted in the MATLAB model. Furthermore, to comprehensively assess the rotational dynamics affecting passenger comfort, the roll and pitch angular accelerations are evaluated using the* $W_{e}$*frequency weighting network. The* $W_{e}$*filter was constructed based on the exact parameters specified in Appendix A of the ISO 2631-1 standard [47] (*$f_{1}=0.4$ *Hz,* $f_{2}=100$ *Hz,* $f_{3}=1.0$ *Hz,* $f_{4}=1.0$ *Hz,* $Q_{4}=0.63$*).”*

*“Upon calculation, at the vehicle's center of gravity, the overall average vertical acceleration using the TD3-based control method is 49.41% lower compared to the passive suspension and 9.33% lower compared to the skyhook control method.”*

*“At high speeds, at the vehicle's center of gravity, the overall average vertical acceleration using the TD3-based control method is 24.82% lower compared to the passive suspension. While it exhibits a distinct dynamic response profile compared to the skyhook control method under high-frequency excitations due to multi-axis posture coordination, the vertical ride comfort remains highly competitive. In the calculation of longitudinal angular acceleration, there's an observed increase when using the TD3-based control method, possibly due to an overall improvement reward.”*

*“At the vehicle's center of gravity, the overall average vertical acceleration using the TD3-based control method is 19.76% lower compared to the passive suspension and 3.19% lower compared to the skyhook control method.”*

## ***5.6 Comprehensive performance evaluation based on ISO 2631-1***

*To provide a comprehensive evaluation of ride comfort under different operating conditions in accordance with the ISO 2631-1 standard, the frequency-weighted RMS accelerations (including vertical* $W_{k}$ *and rotational* $W_{e}$ *indices) for the passive suspension, the classical linear Skyhook control, and the proposed TD3-based method are summarized in Table 7.*

***Table 7. Frequency-weighted RMS accelerations of vehicle suspension under different operating conditions.***

| *Operating Condition* | *Control Method* | *Vertical (*$W_{k}$*) RMS (*$m/s^{2}$*)* | *Roll (*$W_{e}$*) RMS* $rad/s^{2}$ | *Pitch (*$W_{e}$*) RMS* $rad/s^{2}$ |
| --- | --- | --- | --- | --- |
| *Class A (60 km/h)* | *Passive* | *0.1939* | *0.3907* | *0.2610* |
|  | *Skyhook* | *0.1082* | *0.1462* | *0.1377* |
|  | *TD3-based* | *0.0981* | *0.1532* | *0.1414* |
|  | *Fuzzy* | *0.4889* | *0.1893* | *0.1456* |
| *Class A (120 km/s)* | *Passive* | *0.2031* | *0.5016* | *0.2200* |
|  | *Skyhook* | *0.1333* | *0.1939* | *0.1442* |
|  | *TD3-based* | *0.1527* | *0.1587* | *0.1800* |
|  | *Fuzzy* | *0.4515* | *0.2341* | *0.1591* |
| *Class B (60 km/h)* | *Passive* | *0.2647* | *1.5594* | *0.4938* |
|  | *Skyhook* | *0.2194* | *0.7359* | *0.2716* |
|  | *TD3-based* | *0.2124* | *0.8378* | *0.3371* |
|  | *Fuzzy* | *0.5366* | *0.2782* | *0.2453* |

*As comprehensively presented in Table 7, the traditional Fuzzy control method yields vertical frequency-weighted RMS values of 0.4889 (Class A, 60 km/h), 0.4515 (Class A, 120 km/h), and 0.5366 (Class B, 60 km/h). While the Fuzzy controller operates stably within the predefined rule base, its performance in global multi-axis posture coordination (roll and pitch angles) remains constrained. In contrast, the proposed TD3-based method dynamically balances vertical acceleration with rotational angular accelerations through its multi-objective reward mechanism, achieving significantly lower RMS indices across the evaluated scenarios.*

*(3) The article investigates the response of a vehicle suspension controller using the TD3-based control method on Class A road surfaces at speeds of 60 km/h and 120 km/h, as well as on Class B road surfaces at 60 km/h. The results indicate that the vehicle semiactive suspension controlled by the TD3 based method exhibits an average reduction of 19.76 % to 49.41 % in the vertical acceleration of the vehicle body compared to a passive suspension and 3.19 % to 9.33 % compared to the linear Skyhook control method in primary operating conditions. Furthermore the proposed deep reinforcement learning strategy consistently outperforms the traditional Fuzzy control method in suppressing low frequency multi axis vibrations without heavily relying on expert parameter tuning.*

12. “Table 5 & 6. Specification of critic/actor network.” Why these specific numbers of neurons and layers?

**[Response]:** We appreciate the reviewer pointing out the need for more detail regarding the network specifications. As we have clarified in the revised manuscript the Actor and Critic networks are not determined arbitrarily. Starting with two layer networks we tested results with different numbers of layers and nodes in increments of 25. For the Critic network two fully connected layers are sufficient with the number of nodes being critical. Typically a two layer network with the same number of nodes per layer is used. Through testing 75 nodes per layer achieved relatively high computational efficiency and after fine tuning we adopted 100 nodes in the first layer and 75 nodes in the second layer to ensure convergence. Since the Actor network involves similar computations it also uses the same first and second layers as the Critic network. Finally the output must use an arctan layer to ensure that the output range is reliable. See in lines 512-520.

*“The Actor and Critic networks are not determined arbitrarily: starting with two-layer networks, we tested results with different numbers of layers and nodes (in increments of 25). For the Critic network, two fully connected layers are sufficient, with the number of nodes being critical. Typically, a two-layer network with the same number of nodes per layer is used. Through testing, 75 nodes per layer achieved relatively high computational efficiency, and after fine-tuning, we adopted 100 nodes in the first layer and 75 nodes in the second layer to ensure convergence. Since the Actor network involves similar computations, it also uses the same first and second layers as the Critic network. Finally, the output must use an arctan layer to ensure that the output range is reliable.”*

13. Lack of discussion about the hyperparameter tuning and ablation study.

**[Response]:** We appreciate the reviewers constructive feedback regarding the discussion on hyperparameter tuning and ablation studies. As detailed in the revised manuscript the hyperparameters of the TD3 controller were determined through multiple trial and error experiments. All hyperparameters are based on the actual hyperparameters of the model uploaded to the platform. The current hyperparameters are derived from the first training process that achieved relatively good convergence results. In that initial process the number of cycles required for satisfactory convergence was approximately 2500 where each training cycle simulated 30 seconds of suspension time with a total training duration of two and a half days. After several adjustments the current hyperparameters are capable of achieving good convergence within 1000 cycles. Furthermore we have explicitly noted in the text that the model has not yet undergone ablation experiments. See in lines 520-528.

“The hyperparameters of the TD3 controller were determined through multiple trial-and-error experiments. All hyperparameters are based on the actual hyperparameters of the model uploaded to the platform. The current hyperparameters are derived from the first training process that achieved relatively good convergence results. In that initial process, the number of cycles required for satisfactory convergence was approximately 2,500 (each training cycle simulated 30 seconds of suspension time), with a total training duration of two and a half days. After several adjustments, the current hyperparameters are capable of achieving good convergence within 1,000 cycles. The model has not yet undergone ablation experiments.”

14. How do you consider the actuator delay and energy effort of the proposed semi-active suspension?

**[Response]:** We sincerely thank the reviewer for raising this insightful question regarding actuator delay and energy effort since both factors are crucial in real world engineering applications. To address the actuator delay we have added an explanation in the manuscript stating that our numerical simulations utilize a fixed time step of 0.01 seconds. This discrete step size intrinsically reflects the typical physical response delay of practical magnetorheological fluid dampers which generally operate with a millisecond level response time thus implicitly incorporating a basic level of discrete actuator delay into the control loop. Regarding energy effort we agree that power consumption is a vital metric. However the primary scope of this manuscript is to propose the new control framework to resolve convergence issues and minimize vertical acceleration for ride comfort so we have not yet introduced a penalty term for energy consumption into the reward function. Therefore we have explicitly acknowledged this as a limitation in the conclusion section noting that a multi objective reward function balancing ride comfort and energy consumption will be developed in future research to further enhance the engineering applicability of the algorithm. See in lines 494-498 and 814-822.

*“It is worth noting that the simulation time step is set to 0.01 s. This discrete step size intrinsically reflects the typical physical response delay of practical magnetorheological fluid dampers (MFDs), which generally operate with a millisecond-level response time. Thus, a basic level of discrete actuator delay has been implicitly incorporated into the control loop.”*

*“(4) Although the proposed TD3 based control method demonstrates excellent performance in vibration reduction and convergence the current study primarily focuses on the macroscopic dynamic response of the semi active suspension. The highly nonlinear hysteresis characteristics of the MFDs were simplified to prioritize the convergence of the deep reinforcement learning algorithm. In future research hardware in the loop testing will be conducted where actual physical MFDs will be integrated into the control loop to evaluate the robustness of the trained Actor network against unmodeled real world hysteresis. Additionally a multi objective reward function that explicitly balances ride comfort and energy consumption will be developed to further enhance the engineering applicability of the algorithm.”*

15. The comparison is only done with passive suspension and the classical linear Skyhook control method. This does not seem like an attractive contribution for a research paper at 2026! The comparison should be done with a more sophisticated method. For integrated vehicle stability and advanced control comparisons, you may refer to (DOI: 10.1109/ TFUZZ.2026.3695547).

**[Response]:** We sincerely thank the reviewer for the insightful comment and for recommending the highly valuable literature. We completely agree that relying solely on the classical linear Skyhook control is insufficient for a research paper and a more sophisticated benchmark is necessary to demonstrate the proposed algorithm contribution. The recommended literature provided an inspiring perspective on utilizing advanced fuzzy systems for integrated vehicle dynamic control. Inspired by this we have incorporated a sophisticated Fuzzy Logic Control method as our new advanced baseline in the revised manuscript and added corresponding simulation comparisons in Figures 5 to 7. The simulation results demonstrate that while Fuzzy control outperforms the traditional Skyhook method our proposed TD3 based deep reinforcement learning algorithm achieves even greater reduction in vertical acceleration due to its superior adaptability in the complex 7 DOF continuous action space. Furthermore we have carefully studied the recommended paper and cited it in the Introduction to enrich the discussion on cutting edge integrated chassis control. The quantitative comparison results regarding the Fuzzy control baseline have been fully integrated into the Abstract Section 5 and the Conclusion. We believe these additions significantly enhance the rigor of our study. See in lines 10-14, 168-174, 603-612, 638-643, 662-668 and 805-813.

*“The validity of the proposed control method is verified using the developed program under different road roughness and speeds. The results indicate that compared to the passive suspension the classical linear Skyhook control method and the Fuzzy control method the proposed Twin Delayed Deep Deterministic based method achieves superior control effectiveness.”*

*“Furthermore recent studies emphasize the importance of advanced integrated chassis control frameworks. For instance sophisticated methodologies like fuzzy game theoretic tube model predictive control have been developed to ensure robust constraint satisfaction and coordinate heterogeneous actuators for integrated vehicle lateral stability [49]. Inspired by the application of fuzzy logic in advanced vehicle control and to thoroughly validate the proposed TD3 algorithm this paper employs a sophisticated Fuzzy Logic Control algorithm as an advanced benchmark alongside the traditional Skyhook method.”*

*“In addition to the passive suspension and the Skyhook method Fig 5 also presents the response of the Fuzzy control method. As illustrated in Fig 5 the red curve representing the proposed TD3 based method consistently achieves the smallest acceleration amplitudes and the smoothest trajectory among all compared strategies. Although the Fuzzy control method shows a certain degree of vibration reduction compared to the passive suspension its dynamic response exhibits larger peak fluctuations than the proposed method. This occurs because the fixed rule base of the Fuzzy controller lacks sufficient adaptiveness when dealing with the complex multi axis coupling of the 7 DOF full vehicle model. Consequently the proposed TD3 based method demonstrates superior suppression effectiveness and dynamic stability under road surface excitation.”*

*“Similarly as illustrated in Fig 6 the Fuzzy control method struggles to effectively suppress the high frequency excitations at 120 km/h resulting in consistently higher vertical acceleration peaks than the proposed algorithm. Because the predefined fuzzy rules cannot rapidly adapt to the intensified road inputs the proposed TD3 based control method demonstrates significantly superior high speed handling capability and continuous adaptiveness compared to the conventional Fuzzy control method.”*

*“Under the harsher Class B road conditions depicted in Fig 7 the conventional Fuzzy control method shows pronounced oscillations and fails to optimally coordinate the multi axis dynamics. Due to the severe road undulations the fixed rule base of the Fuzzy controller becomes inadequate. In contrast the proposed TD3 based control method significantly outperforms the Fuzzy control method by maintaining a more stable vehicle body posture and effectively suppressing extreme acceleration peaks which further validates its exceptional robustness on rough road surfaces.”*

*“(3) The article investigates the response of a vehicle suspension controller using the TD3-based control method on Class A road surfaces at speeds of 60 km/h and 120 km/h, as well as on Class B road surfaces at 60 km/h. The results indicate that the vehicle semiactive suspension controlled by the TD3 based method exhibits an average reduction of 19.76 % to 49.41 % in the vertical acceleration of the vehicle body compared to a passive suspension and 3.19 % to 9.33 % compared to the linear Skyhook control method in primary operating conditions. Furthermore the proposed deep reinforcement learning strategy consistently outperforms the traditional Fuzzy control method in suppressing low frequency multi axis vibrations without heavily relying on expert parameter tuning.”*

16. The results only show time-domain responses. What about the frequency-domain analysis? The vibration performance should be evaluated by the frequency-based approach.

**[Response]:** We sincerely thank the reviewer for this constructive and insightful comment, and we completely agree that evaluating the suspension system from a frequency-based perspective is essential for a comprehensive and rigorous performance assessment in alignment with the ISO 2631 standard regarding human vibration sensitivity. To address this valuable suggestion, we have significantly expanded the manuscript by incorporating a dedicated frequency-domain analysis in Section 5.8, where power spectral density responses within the critical 0~30Hz frequency band are thoroughly evaluated across Class A road at 60 km/h, Class A road at 120 km/h, and Class B road at 60 km/h conditions for the proposed TD3 method, classical Skyhook control, traditional Fuzzy control, and passive suspension. The newly incorporated results and Figs 14–16 demonstrate that the proposed TD3 strategy effectively mitigates the massive resonance energy of the sprung mass in the critical low-frequency band (around 1~2 Hz) and robustly suppresses resonance peaks under varying speeds and road roughness without requiring manual parameter tuning, while supporting numerical data have been updated in Supporting Information files S9–S11 Data and corresponding updates have been seamlessly integrated into the Abstract, Section 5.8, and Conclusion to highlight these findings. See in lines 22-28 and 737-794.

*“Furthermore the power spectral density analysis demonstrates that the proposed method significantly suppresses the low frequency resonance of the sprung mass across varying road conditions compared to the passive Skyhook and Fuzzy methods, further validating its superior broadband vibration isolation capability. This demonstrates that the Twin Delayed Deep Deterministic-based control method for the vehicle semi-active suspension model has the advantages of fast convergence speed and good control effectiveness under road surface excitation.”*

## ***5.8 Frequency domain analysis***

*To further reveal the vibration isolation performance of different control strategies under realistic driving conditions, the power spectral density is introduced to analyze the dynamic responses of the vehicle system in the frequency domain. According to the ISO 2631 standard, the human body is most sensitive to vibrations at low frequencies, particularly in the range from 4 to 8 Hz for the vertical direction and 1 to 2 Hz for rotational directions. Furthermore, the natural frequency of the vehicle sprung mass typically concentrates within the 1 to 3 Hz range. Therefore, this section primarily evaluates the power spectral density curves of vertical acceleration, roll angular acceleration, and pitch angular acceleration within the frequency from 0 to 30 Hz. Figs 14, 15, and 16 illustrate the frequency responses of the vehicle under Class A at 60 km per h, Class A at 120 km per h, and Class B at 60 km per h road conditions respectively.*

***Fig 14. Power spectral density (PSD) of vehicle dynamic responses under Class A road excitation at 60 km/h: (a) Vertical acceleration; (b) Roll angular acceleration; (c) Pitch angular acceleration.***

***Fig 15. Power spectral density (PSD) of vehicle dynamic responses under Class A road excitation at 120 km/h: (a) Vertical acceleration; (b) Roll angular acceleration; (c) Pitch angular acceleration.***

***Fig 16. Power spectral density (PSD) of vehicle dynamic responses under Class B road excitation at 60 km/h: (a) Vertical acceleration; (b) Roll angular acceleration; (c) Pitch angular acceleration.***

*Under the Class A road excitation, a significant resonance peak at low frequencies emerges between 1 and 1.5 Hz for the passive suspension across both speed scenarios. Specifically, at 60 km per h, the passive suspension reaches a power spectral density peak of 1.36* $\left( m/s^{2} \right)^{2}/Hz$ *at 1.22 Hz in the vertical direction. The proposed TD3 strategy effectively suppresses this peak to 0.126* $\left( m/s^{2} \right)^{2}/Hz$*, outperforming the passive system, the classical Skyhook controller which has a peak of 0.17* $\left( m/s^{2} \right)^{2}/Hz$*, and the Fuzzy method. Notably, the Fuzzy method exhibits a severe vertical resonance peak of 33.18* $\left( m/s^{2} \right)^{2}/Hz$ *shifted to 2.93 Hz, indicating poor frequency domain performance. When the speed increases to 120 km per h, the intensified road excitation causes the vertical resonance peak of the passive suspension to surge to 7.918* $\left( m/s^{2} \right)^{2}/Hz$*. In contrast, the TD3 strategy robustly limits the peak to 0.607* $\left( m/s^{2} \right)^{2}/Hz$*, demonstrating strong adaptiveness to varying speeds, while the Fuzzy method again shows a massive peak of 27.64* $\left( m/s^{2} \right)^{2}/Hz$ *at 2.69 Hz. Regarding rotational dynamics, both the TD3 and Skyhook methods substantially mitigate the resonance energy. For instance, at 60 km per h on the Class A road, the TD3 strategy reduces the roll angular acceleration peak from 15.26* $\left( rad/s^{2} \right)^{2}/Hz$ *for the passive suspension to 8.32* $\left( rad/s^{2} \right)^{2}/Hz$*, thereby enhancing the vehicle posture stability, whereas the Fuzzy method yields a peak of 16.63* $\left( rad/s^{2} \right)^{2}/Hz$ *at 2.20 Hz.*

*Under the harsher Class B road excitation at 60 km per h, the severe road undulations at low frequencies induce intense body posture variations. The data reveals that the peaks for roll and pitch angular accelerations of the passive suspension soar to 31.83* $\left( rad/s^{2} \right)^{2}/Hz$ *and 25.28* $\left( rad/s^{2} \right)^{2}/Hz$ *respectively in the range of 0.7 to 1.7 Hz. The Fuzzy method exacerbates this instability, pushing the pitch peak to an extreme 73.39* $\left( rad/s^{2} \right)^{2}/Hz$ *at 4.64 Hz and the vertical peak to 21.69* $\left( m/s^{2} \right)^{2}/Hz$ *at 2.69 Hz. Under this condition, the TD3 controller maintains comprehensive control benefits. Although the specifically tuned Skyhook method exhibits strong forced damping characteristics in suppressing certain angular acceleration peaks, the proposed model independent data driven TD3 strategy intelligently finds a globally optimal balance between vertical vibration isolation and posture control without manual parameter tuning. This avoids the riding harshness often caused by excessive damping in a single degree of freedom.*

*Furthermore, observing the frequency band from 10 to 30 Hz across all conditions reveals that the power spectral density curves of the passive suspension generally remain at the lowest energy levels. Conversely, the high frequency values of the Skyhook and proposed TD3 strategies are slightly higher than that of the passive suspension. This represents a typical waterbed effect in semi active suspension control where substantially suppressing the massive energy in the low frequency resonance region causes the controller to rapidly adjust the damping force, inevitably transferring a minimal amount of energy to higher frequencies. However, since the absolute values of the vibration energy in this high frequency band are extremely small, and such high frequency chattering is easily filtered by the vehicle seats and rubber bushings, it does not negatively impact the subjective ride comfort of the passengers.*

*(6) The frequency domain analysis via power spectral density confirms that the TD3 strategy effectively mitigates the resonance energy of the sprung mass in the critical low frequency band from 0 to 30 Hz. Notably it robustly suppresses the fundamental resonance peaks around 1 to 2 Hz under both Class A and Class B road excitations without inducing high frequency chattering, outperforming the passive Skyhook and Fuzzy methods, thereby achieving a superior balance between vertical ride comfort and rotational posture stability.*

17. “Fig 5, 6, 7.” Please upgrade the page arrangements. Modify the page design to avoid a figure break during the page and column changes!

**[Response]:** We sincerely thank the reviewer for pointing this out. We have optimized the page arrangements in the revised manuscript. Specifically, we have applied paragraph formatting controls ("keep with next" and "keep lines together") to the figure captions to ensure they remain bound to their respective figures. Furthermore, we have verified that the source files for Figures 5, 6, and 7 are properly sized and consolidated to avoid any unwanted figure breaks across pages or columns in the compiled document. see in lines 565, 619 and 650.

18. How do you evaluate the active suspension energy consumption and optimize it?

**[Response]:** We sincerely thank the reviewer for this insightful question regarding the evaluation and optimization of energy consumption. The evaluation of the energy consumption does not require altering the main control architecture. From a post processing perspective, the electrical power and energy can be evaluated using the output control current time series data combined with the damper coil resistance, while the mechanical power dissipation can be quantified through the product of the damping force and the suspension relative velocity. The primary focus of the current study is to establish a robust and fast converging TD3 based control framework for the high dimensional full vehicle model to maximize ride comfort by suppressing vertical accelerations. As explicitly noted in our manuscript Conclusion section, the energy consumption has not yet been integrated into the real time reward function. To achieve explicit energy optimization, future work will focus on developing a multi objective reward function that explicitly balances ride comfort and energy consumption to further enhance the engineering applicability of the algorithm. See in lines 814-822.

*“(4) Although the proposed TD3 based control method demonstrates excellent performance in vibration reduction and convergence the current study primarily focuses on the macroscopic dynamic response of the semi active suspension. The highly nonlinear hysteresis characteristics of the MFDs were simplified to prioritize the convergence of the deep reinforcement learning algorithm. In future research hardware in the loop testing will be conducted where actual physical MFDs will be integrated into the control loop to evaluate the robustness of the trained Actor network against unmodeled real world hysteresis. Additionally a multi objective reward function that explicitly balances ride comfort and energy consumption will be developed to further enhance the engineering applicability of the algorithm.”*

19. How much is the possibility of implementing your proposed method on a cost-effective microcontroller?

**[Response]:** We sincerely appreciate your insightful comment regarding the practical implementation of the proposed method. To address this concern, we clarify that the heavy computational burden of the algorithm occurs exclusively during the offline training phase. In the online execution phase on the vehicle, only the trained Actor network is deployed in the microcontroller to generate control commands. The Actor network is highly lightweight and consists of three fully connected layers containing exactly 10079 parameters. Using standard floating point representation, the entire network requires roughly 40 kilobytes of storage space, which easily fits into the memory capacity of modern economical automotive microcontrollers. Furthermore, a single forward pass through this Actor network requires approximately 20000 floating point operations. A standard affordable microcontroller operating at a modest frequency can comfortably execute this computation in a fraction of a millisecond, which is well within the 0.01 second discrete simulation time step adopted in our study to reflect the practical physical delay of the dampers. Therefore, implementing the proposed trained policy on an economical microcontroller is highly feasible without sacrificing real time control performance, and we have added a dedicated discussion on this hardware deployment feasibility in the Conclusion section of the revised manuscript. See in lines 498-502 and 814-822.

*“Furthermore, the online execution of the trained Actor network requires only a single forward pass per time step. With approximately 10,000 network parameters that require roughly 40 KB of memory and 20,000 floating-point operations, the computational time on a standard cost-effective microcontroller is well within the 0.01 s discrete step size, ensuring real-time control feasibility.”*

*“(4) Although the proposed TD3 based control method demonstrates excellent performance in vibration reduction and convergence the current study primarily focuses on the macroscopic dynamic response of the semi active suspension. The highly nonlinear hysteresis characteristics of the MFDs were simplified to prioritize the convergence of the deep reinforcement learning algorithm. In future research hardware in the loop testing will be conducted where actual physical MFDs will be integrated into the control loop to evaluate the robustness of the trained Actor network against unmodeled real world hysteresis. Additionally a multi objective reward function that explicitly balances ride comfort and energy consumption will be developed to further enhance the engineering applicability of the algorithm.”*

**Responses to Reviewer #2**

1. The main contribution of the proposed TD3-based controller is not sufficiently distinguished from existing DRL-based suspension control methods. The manuscript mainly applies the standard TD3 algorithm with modifications of network structure, training parameters, and reward function, but the specific methodological innovations and their contributions to improving suspension control performance are not clearly explained. The authors should further clarify what fundamental improvements are introduced compared with conventional DRL control frameworks.

**[Response]:** We sincerely thank the reviewer for this insightful and highly constructive comment. We fully agree that the underlying TD3 algorithm is a standard, established algorithm in the machine learning field. Inspired by your feedback, we realize that our previous manuscript did not adequately articulate our methodological innovations, which lie not in inventing a new mathematical DRL algorithm, but in designing a domain-specific, physically-informed DRL control framework tailored to overcome the high-dimensional, nonlinear challenges of a 7-DOF full-vehicle semi-active suspension system.

To clarify the fundamental improvements of our proposed framework over conventional DRL methods, we have made comprehensive revisions to the manuscript, specifically focusing on the physical and engineering justifications for our design choices.

The detailed modifications are as follows:

(1) Clarification of the Methodological Framework:

We have revised the Abstract and the end of the Introduction to explicitly state our core contribution. We clarified that conventional algorithms like DDPG suffer from severe Q-value overestimation in highly coupled, multi-axis road excitation environments. The introduction of the TD3 architecture, particularly its twin delayed critics, is specifically leveraged as a fundamental mechanism to ensure stable policy evaluation under these complex dynamics, rather than just a simple algorithm replacement. See in lines 2-9 and 149-157.

*“To improve the convergence issues of deep reinforcement learning in the control of vehicle semi-active suspension, and enhance the control effectiveness of the vehicle's semi-active suspension, a Twin Delayed Deep Deterministic-based control framework tailored for the high-dimensional nonlinear dynamics of the vehicle semi-active suspension model is proposed. Rather than merely applying standard deep reinforcement learning, this study introduces a domain-specific state-action mapping mechanism and a multi-objective reward function to physically constrain the neural network outputs and coordinate the full-vehicle dynamic responses.”*

*“Specifically, the fundamental improvements of the proposed TD3 framework over conventional DRL methods lie in two aspects. First, unlike the DDPG algorithm which suffers from severe Q-value overestimation in the highly coupled 7-DOF full-vehicle environment, the integrated TD3 architecture employs twin delayed critics to ensure stable policy evaluation under complex multi-axis road excitations. Second, a customized state-action mapping and a physically meaningful reward function are designed to bridge the gap between unconstrained DRL algorithms and the strict physical boundaries of magnetorheological fluid dampers (MFDs), ensuring that the generated control currents are both theoretically optimal and physically executable.”*

(2) Physical Justification of the Action Space Mapping:

We have expanded the explanation of the action space design. We clarified that the use of the arctangent function is not merely a mathematical adjustment, but a smooth, continuous constraint mechanism designed to intrinsically map the neural network's exploratory outputs into the strict physical boundaries of the magnetorheological fluid damper (MFD). This specific mapping prevents actuator saturation and high-frequency chattering, which are common issues in conventional DRL applications with unbounded outputs. See in lines 375-382.

*“In conventional DRL applications, unbounded action outputs often lead to actuator saturation or sudden jolts in physical systems. To fundamentally address this, each real number in the action space vector of the proposed TD3 algorithm is processed through the arctangent function. This specific design acts as a smooth, continuous constraint mechanism, intrinsically mapping the neural network's exploratory outputs into the strict physical boundary of the MFD's maximum and minimum current limits, thereby preventing high-frequency chattering in the semi-active dampers.”*

(3) Redefinition of the Reward Function as a Multi-Objective Optimization:

We have entirely rewritten the description of the reward function to emphasize its methodological significance. As requested by the reviewer, we clarified that our reward function is designed as an optimization index with multiple objectives tailored for the dynamics of the entire vehicle. The framework assigns strict weighting coefficients based on human physiological sensitivity. By prioritizing the minimization of vertical acceleration while restricting roll and pitch angular accelerations, this approach compels the agent to learn a globally optimal damping strategy across all four wheels. This represents a significant improvement over conventional decentralized controllers that rely primarily on localized feedback. See in lines 417-427.

*“Unlike standard DRL tasks that rely on simple goals, the reward function in this proposed framework is fundamentally designed as an optimization index with multiple objectives tailored for the dynamics of the entire vehicle. Specifically, the reward for the current action is formulated as a comprehensive weighted combination of the velocities and accelerations associated with the vertical, roll, and pitch degrees of freedom at the vehicle center of mass. The weighting coefficients are meticulously assigned based on human physiological sensitivity, prioritizing the minimization of vertical acceleration to enhance ride comfort, while simultaneously restricting roll and pitch angular accelerations to ensure handling stability. This specialized reward structure compels the TD3 agent to learn a globally optimal damping strategy across all four wheels, representing a clear methodological improvement over conventional decentralized controllers that rely primarily on localized feedback.”*

We believe these revisions address your concerns by firmly grounding our DRL parameter and network modifications in physical vehicle dynamics and engineering methodology. We are deeply grateful for your guidance, which has significantly enhanced the theoretical depth of our manuscript.

2. The design of the state space, action space, and reward function is critical for reinforcement learning-based vehicle control, but the current explanation is insufficient.

**[Response]:** We sincerely thank the reviewer for highlighting the importance of the reinforcement learning design parameters. As detailed in the revised manuscript to address the concerns regarding the reward function we have clarified that it has been tested and employs a weighted sum of the vertical acceleration and vertical velocity at the transformed vehicle center of mass achieving relatively effective control performance. In the vehicle suspension model angular velocity angular acceleration vertical velocity acceleration and vibration frequency at the vehicle center of mass are key parameters for damping while our primary concern is vertical acceleration and frequency. Extensive testing shows that using angular velocity or angular acceleration causes the TD3 control method to favor obtaining more rewards for those parameters and setting a reward cap provides little benefit. When vertical acceleration alone is used as the reward function input the control algorithm tends to prioritize higher rewards at the expense of controlling the suspension deformation direction resulting in uncontrollable vertical displacement changes under control. Therefore introducing velocity weighting not only keeps the suspension motion range within a controllable scope but as tests indicate a reasonable velocity weight can adjust the suspension vibration frequency to a range less sensitive to human perception. For the reward function in the TD3 controller frequency inputs need to be considered holistically in the frequency domain which leads to more complex policy outcomes that are difficult to derive quickly. Thus acceleration weighting can reduce the vibrations transmitted from the suspension to the vehicle body while velocity weighting can control the suspension motion range and implicitly regulate the vibration frequency transmitted to the vehicle body. See in lines 396-414 and 512-528.

*“The reward function has been tested and employs a weighted sum of the vertical acceleration and vertical velocity at the transformed vehicle center of mass, achieving relatively effective control performance: in the vehicle suspension model, angular velocity, angular acceleration, vertical velocity, acceleration, and vibration frequency at the vehicle center of mass are key parameters for damping, while our primary concern is vertical acceleration and frequency. Extensive testing shows that using angular velocity/angular acceleration causes the TD3 control method to favor obtaining more rewards for angular velocity/angular acceleration, and setting a reward cap provides little benefit. When vertical acceleration alone is used as the reward function input, the control algorithm tends to prioritize higher rewards at the expense of controlling the suspension's deformation direction, resulting in uncontrollable vertical displacement changes under control. Therefore, introducing velocity weighting not only keeps the suspension's motion range within a controllable scope but, as tests indicate, a reasonable velocity weight can adjust the suspension's vibration frequency to a range less sensitive to human perception. For the reward function in the TD3 controller, frequency inputs need to be considered holistically in the frequency domain, which leads to more complex policy outcomes that are difficult to derive quickly. Thus, acceleration weighting can reduce the vibrations transmitted from the suspension to the vehicle body, while velocity weighting can control the suspension's motion range and implicitly regulate the vibration frequency transmitted to the vehicle body.”*

*“The Actor and Critic networks are not determined arbitrarily: starting with two-layer networks, we tested results with different numbers of layers and nodes (in increments of 25). For the Critic network, two fully connected layers are sufficient, with the number of nodes being critical. Typically, a two-layer network with the same number of nodes per layer is used. Through testing, 75 nodes per layer achieved relatively high computational efficiency, and after fine-tuning, we adopted 100 nodes in the first layer and 75 nodes in the second layer to ensure convergence. Since the Actor network involves similar computations, it also uses the same first and second layers as the Critic network. Finally, the output must use an arctan layer to ensure that the output range is reliable.”*

3. The reward function mainly considers velocity and acceleration-related variables, while other important suspension performance objectives, such as suspension working space, tire-road contact condition, and actuator constraints, are not explicitly considered.

**[Response]:** We sincerely thank the reviewer for this insightful comment. We have thoroughly addressed the concerns regarding suspension working space, tire road contact condition, and actuator constraints in the revised manuscript.

First actuator constraints are strictly and explicitly guaranteed by the proposed state to action mapping mechanism rather than the reward function. The arctangent activation function inherently bounds the exploratory actions within the strict physical limits of the minimum and maximum currents of the damper.

Furthermore instead of artificially adding penalty terms for the suspension working space and dynamic tire load into the reward function this study relies on the coupled nature of the 7 DOF vehicle dynamics. Such artificial penalty terms often lead to reward shaping complexity and conflicting gradient directions. By suppressing the overall roll and pitch angular accelerations the extreme deflections at the four corners are intrinsically mitigated which implicitly ensures contact between the tires and the road and prevents suspension stroke saturation.

As detailed in the newly added Section 5.7 along with Tables 8 and 9 a systematic evaluation reveals that under standard Class A road excitations the proposed TD3 based controller implicitly maintains these safety constraints within optimal bounds. However under harsher Class B road conditions the unconstrained agent aggressively sacrifices tire force stability and suspension limits to prioritize ride comfort. This finding validates the importance of explicitly monitoring the suspension working space and dynamic tire load and indicates that incorporating these safety constraints into a multi objective reward function will be a crucial step for future physical deployments. See in lines 447-457, 684-736 and 823-830.

*“It is worth noting that while the reward function primarily focuses on variables related to velocity and acceleration to maximize ride comfort actuator constraints are strictly and explicitly guaranteed by the proposed state to action mapping mechanism. As shown in Eq (4.11) the arctangent activation function inherently bounds the exploratory actions within the strict physical limits of the MFD minimum and maximum currents. Furthermore instead of artificially adding penalty terms for the suspension working space and dynamic tire load into the reward function as this often leads to reward shaping complexity and conflicting gradient directions this study relies on the coupled nature of the 7 DOF vehicle dynamics. By suppressing the overall roll and pitch angular accelerations the extreme deflections at the four corners are intrinsically mitigated thus implicitly ensuring contact between the tires and the road and preventing suspension stroke saturation.”*

## ***5.7 Evaluation of Suspension Working Space and Tire-road Contact***

*To address potential concerns regarding the safety constraints of the suspension system, the dynamic tire load (DTL) and suspension working space (SWS) are systematically evaluated. Although these objectives were not explicitly penalized in the reward function, the comprehensive posture optimization of the 7-DOF model implicitly maintains these constraints.*

*To quantitatively verify the performance across all simulated scenarios, the root mean square (RMS) values of SWS and DTL under three typical conditions are summarized in Tables 8 and 9, respectively. Furthermore, the complete time-domain responses of SWS and DTL for the four wheels (FL, FR, RL, RR) are illustrated in Figs 8–13.*

***Fig 8. Time-domain responses of suspension working space (SWS) under Class A road excitation at 60 km/h (Condition 1). (a) FL wheel. (b) FR wheel. (c) RL wheel. (d) RR wheel.***

***Fig 9. Time-domain responses of dynamic tire load (DTL) under Class A road excitation at 60 km/h (Condition 1). (a) FL wheel. (b) FR wheel. (c) RL wheel. (d) RR wheel.***

***Fig 10. Time-domain responses of suspension working space (SWS) under Class A road excitation at 120 km/h (Condition 2). (a) FL wheel. (b) FR wheel. (c) RL wheel. (d) RR wheel.***

***Fig 11. Time-domain responses of dynamic tire load (DTL) under Class A road excitation at 120 km/h (Condition 2). (a) FL wheel. (b) FR wheel. (c) RL wheel. (d) RR wheel.***

***Fig 12. Time-domain responses of suspension working space (SWS) under Class B road excitation at 60 km/h (Condition 3). (a) FL wheel. (b) FR wheel. (c) RL wheel. (d) RR wheel.***

***Fig 13. Time-domain responses of dynamic tire load (DTL) under Class B road excitation at 60 km/h (Condition 3). (a) FL wheel. (b) FR wheel. (c) RL wheel. (d) RR wheel.***

***Table 8. Comparison of the RMS values of suspension working space (SWS) under different driving conditions.***

| **Table 8. Comparison of the RMS values of suspension working space (SWS) under different driving conditions.** | | | | | |
| --- | --- | --- | --- | --- | --- |
| *Driving Condition* | *Position* | *RMS (m)* | | | |
|  |  | *Passive* | *Skyhook* | *TD3-based* | *FUZZY* |
| *Class A (60 km/h)* | *FL* | *0.2206* | *0.2331* | *0.2229* | *0.2612* |
|  | *FR* | *0.2539* | *0.2605* | *0.2517* | *0.2873* |
|  | *RL* | *0.1763* | *0.1783* | *0.1754* | *0.2031* |
|  | *RR* | *0.1679* | *0.1799* | *0.2242* | *0.2002* |
| *Class A (120 km/h)* | *FL* | *0.2285* | *0.2394* | *0.1731* | *0.2591* |
|  | *FR* | *0.2500* | *0.2600* | *0.2509* | *0.2883* |
|  | *RL* | *0.1794* | *0.1854* | *0.1759* | *0.2059* |
|  | *RR* | *0.1678* | *0.1819* | *0.1739* | *0.2024* |
| *Class B (60 km/h)* | *FL* | *0.2291* | *0.2539* | *0.2354* | *0.2978* |
|  | *FR* | *0.3291* | *0.3141* | *0.3326* | *0.3559* |
|  | *RL* | *0.2511* | *0.2149* | *0.1941* | *0.2328* |
|  | *RR* | *0.1899* | *0.2008* | *0.2652* | *0.2254* |

***Table 9. Comparison of the RMS values of dynamic tire load (DTL) under different driving conditions.***

| *Driving Condition* | *Position* | *RMS (N)* | | | |
| --- | --- | --- | --- | --- | --- |
|  |  | *Passive* | *Skyhook* | *TD3-based* | *FUZZY* |
| *Class A (60 km/h)* | *FL* | *5062.93* | *4805.45* | *4937.27* | *6929.79* |
|  | *FR* | *3993.74* | *3400.79* | *3594.86* | *5830.49* |
|  | *RL* | *3931.91* | *3415.10* | *4912.57* | *5338.41* |
|  | *RR* | *5076.81* | *4821.48* | *3611.20* | *6560.95* |
| *Class A (120 km/h)* | *FL* | *5271.74* | *4931.35* | *4989.81* | *7345.98* |
|  | *FR* | *3897.24* | *3528.26* | *3582.37* | *7015.60* |
|  | *RL* | *3830.92* | *3478.21* | *4959.32* | *5978.93* |
|  | *RR* | *5125.91* | *4881.49* | *3634.92* | *7146.13* |
| *Class B (60 km/h)* | *FL* | *5675.98* | *5401.11* | *5894.40* | *8920.70* |
|  | *FR* | *11138.51* | *5047.70* | *11779.85* | *9808.07* |
|  | *RL* | *22891.64* | *6067.07* | *13965.92* | *9022.89* |
|  | *RR* | *12594.65* | *5426.79* | *24778.73* | *9910.89* |

*As presented in Table 8 and visually confirmed by Figs 8 and 10, under standard smooth road conditions, including a normal speed of 60 km/h and a high speed of 120 km/h, the proposed TD3 algorithm effectively restricts the SWS fluctuations within optimal physical bounds. For instance on the Class A road at 60 km/h the RMS of the TD3 method on the front left wheel is 0.2229 m which is smaller than that of the Skyhook control with 0.2331 m and the Fuzzy control with 0.2612 m. Similarly, as shown in Table 9 and Figs 9 and 11, the dynamic tire loads maintain highly stable trajectories during both low-speed and high-speed driving on Class A roads. The RMS values of the TD3 method typically range from 3500 N to 5300 N which are comparable to the vehicle static gravitational load whereas the Fuzzy control exhibits larger fluctuations up to 7345 N thereby implicitly ensuring continuous contact with the road surface without requiring explicit penalty terms.*

*However, an insightful control trade-off is revealed under the harsher Class B road excitation at 60 km/h. As the reward function primarily drives the agent to minimize the sprung mass accelerations, the TD3 algorithm actively allocates much larger dynamic loads to individual wheels to stabilize the vehicle body. Consequently as observed in Table 9 and clearly depicted by the extreme transient peaks in Fig 13 the DTL responses of TD3 exhibit significant increases on the rear left and front right wheels with DTL RMS values reaching up to 24778 N and 11779 N respectively compared to the conservative Skyhook and Fuzzy methods. Although the SWS limits are still physically maintained (Fig 12), this extreme operating condition demonstrates that while unconstrained DRL models excel at isolating vibrations on standard roads, they tend to aggressively sacrifice tire force stability to maximize ride comfort under severe excitations. This finding validates the importance of actuator constraints and indicates that explicitly incorporating DTL and SWS into the multi-objective reward function will be a crucial step for the future physical deployment of DRL-based active suspensions.*

*“(5) The systematic evaluation of the suspension working space and dynamic tire load reveals an insightful control trade off. Under standard Class A road excitations the proposed TD3 based controller implicitly maintains these safety constraints within optimal bounds without requiring explicit penalty terms. However under harsher Class B road conditions the unconstrained DRL agent aggressively sacrifices tire force stability and suspension limits to prioritize ride comfort. This phenomenon validates the necessity of explicitly incorporating these safety constraints into a multi objective reward function which will be an essential step for the future physical deployment of DRL based active suspensions.”*

4. The authors should provide a more comprehensive discussion on how the reward function reflects the multi-objective requirements of semi-active suspension systems.

**[Response]:** We appreciate the reviewer for highlighting the need for a deeper analysis of the relationship between the TD3 controller and the physical characteristics of the semi active suspension system. We have significantly expanded our discussion in the revised manuscript to elaborate on this relationship. The optimization goals are integrated into a cohesive weighting hierarchy to reflect the physical multi objective requirements. To effectively isolate the passenger cabin from road disturbances, the absolute highest penalty weights are assigned to the vertical acceleration and velocity. This establishes ride comfort as the primary optimization objective and ensures the principal direction focuses strictly on minimizing human body vibration exposure. Because semi active suspensions must also maintain the posture and safety of the vehicle, secondary penalty weights are applied to the roll and pitch angular accelerations to ensure handling stability. This secondary weighting deliberately prevents the agent from adopting overly soft damping strategies that would otherwise cause excessive body roll or pitch during dynamic excitations. By balancing these hierarchical weights, the reward function systematically resolves the inherent physical trade offs in full vehicle chassis control, which prevents the agent from greedily optimizing for vertical comfort at the catastrophic expense of vehicle stability. See in lines 428-440.

*“To further elaborate on how the reward function reflects these multi-objective requirements, the optimization goals are integrated into a cohesive weighting hierarchy. To effectively isolate the passenger cabin from road disturbances, the absolute highest penalty weights are assigned to the vertical acceleration and velocity, establishing ride comfort as the primary optimization objective. This ensures that the TD3 agent's principal direction focuses strictly on minimizing human body vibration exposure. Simultaneously, because semi-active suspensions must also maintain the vehicle's posture and safety, secondary penalty weights are applied to the roll and pitch angular accelerations to ensure handling stability. This deliberately prevents the agent from adopting overly soft damping strategies that would otherwise cause excessive body roll or pitch during dynamic excitations. By balancing these hierarchical weights, the reward function systematically resolves the inherent physical trade-offs in full-vehicle chassis control, preventing the DRL agent from greedily optimizing for vertical comfort at the catastrophic expense of vehicle stability.”*

5. The relationship between the TD3 controller and the physical characteristics of the semi-active suspension system requires further analysis.

**[Response]:** We sincerely thank the reviewer for highlighting the need to further analyze the relationship between the TD3 controller and the physical characteristics of the semi active suspension system. As detailed in the revised manuscript the reward function has been tested and employs a weighted sum of the vertical acceleration and vertical velocity at the transformed vehicle center of mass achieving relatively effective control performance. In the vehicle suspension model angular velocity angular acceleration vertical velocity acceleration and vibration frequency at the vehicle center of mass are key parameters for damping while our primary concern is vertical acceleration and frequency. Extensive testing shows that using angular velocity or angular acceleration causes the TD3 control method to favor obtaining more rewards for those parameters and setting a reward cap provides little benefit. When vertical acceleration alone is used as the reward function input the control algorithm tends to prioritize higher rewards at the expense of controlling the suspension deformation direction resulting in uncontrollable vertical displacement changes under control. Therefore introducing velocity weighting not only keeps the suspension motion range within a controllable scope but as tests indicate a reasonable velocity weight can adjust the suspension vibration frequency to a range less sensitive to human perception. For the reward function in the TD3 controller frequency inputs need to be considered holistically in the frequency domain which leads to more complex policy outcomes that are difficult to derive quickly. Thus acceleration weighting can reduce the vibrations transmitted from the suspension to the vehicle body while velocity weighting can control the suspension motion range and implicitly regulate the vibration frequency transmitted to the vehicle body. See in lines 396-414.

*“The reward function has been tested and employs a weighted sum of the vertical acceleration and vertical velocity at the transformed vehicle center of mass, achieving relatively effective control performance: in the vehicle suspension model, angular velocity, angular acceleration, vertical velocity, acceleration, and vibration frequency at the vehicle center of mass are key parameters for damping, while our primary concern is vertical acceleration and frequency. Extensive testing shows that using angular velocity/angular acceleration causes the TD3 control method to favor obtaining more rewards for angular velocity/angular acceleration, and setting a reward cap provides little benefit. When vertical acceleration alone is used as the reward function input, the control algorithm tends to prioritize higher rewards at the expense of controlling the suspension's deformation direction, resulting in uncontrollable vertical displacement changes under control. Therefore, introducing velocity weighting not only keeps the suspension's motion range within a controllable scope but, as tests indicate, a reasonable velocity weight can adjust the suspension's vibration frequency to a range less sensitive to human perception. For the reward function in the TD3 controller, frequency inputs need to be considered holistically in the frequency domain, which leads to more complex policy outcomes that are difficult to derive quickly. Thus, acceleration weighting can reduce the vibrations transmitted from the suspension to the vehicle body, while velocity weighting can control the suspension's motion range and implicitly regulate the vibration frequency transmitted to the vehicle body.”*

6. The proposed method relies on data-driven learning, but the influence of vehicle dynamics characteristics, damper nonlinearities, and actuator limitations on the learned control policy is not sufficiently discussed.

**[Response]:** We sincerely thank the reviewer for highlighting the need to discuss the physical influences on the learned policy. To address actuator limitations, we clarified that our specific mapping mechanism structurally encodes physical current boundaries into the neural network's output via the arctan function. This fundamentally prevents the policy from learning physically un-executable commands. Combined with the discrete simulation time step of 0.01 s which implicitly incorporates the physical response delay of the MFD, the reinforcement learning framework ensures that the obtained control strategy strictly respects both the amplitude and temporal limitations of practical actuators. Regarding vehicle dynamics characteristics, the learned control policy is profoundly shaped by penalizing pitch and roll angular accelerations within the seven degree of freedom full vehicle model. The policy intrinsically accounts for the dynamic cross coupling effects across the longitudinal and lateral axes. This compels the TD3 agent to learn a globally coordinated damping strategy, thereby mitigating the typical waterbed effect wherein enhancing vertical ride comfort at an individual corner severely deteriorates the overall dynamic posture of the vehicle. Finally, to explain the influence of damper nonlinearities, we added discussions showing that the control current profiles demonstrate how the TD3 agent implicitly learns to navigate this physical nonlinearity. Rather than generating aggressive, unbounded current commands that would fall into the unresponsive saturation zones of the damper, the learned policy adapts by continuously adjusting the control current to operate preferentially within the sensitive pseudo-linear regions of the force-velocity profile of MFD, thereby maximizing vibration isolation efficiency. See in lines 388-394, 440-446 and 593-602.

*“This specific mapping mechanism explicitly addresses the actuator limitations of the semi-active suspension system. By structurally encoding the physical current boundaries into the neural network's output via the* $arctan$ *function, the policy is fundamentally prevented from learning physically un-executable commands. Furthermore, combined with the discrete simulation time step of 0.01 s which implicitly incorporates the physical response delay of the MFD, the reinforcement learning framework ensures that the obtained control strategy strictly respects both the amplitude and temporal limitations of practical actuators.”*

*“Therefore, the learned control policy is profoundly shaped by the inherent dynamic characteristics of the vehicle. By penalizing pitch and roll angular accelerations within the seven degree of freedom full vehicle model, the policy intrinsically accounts for the dynamic cross coupling effects across the longitudinal and lateral axes. This compels the TD3 agent to learn a globally coordinated damping strategy, thereby mitigating the typical waterbed effect wherein enhancing vertical ride comfort at an individual corner severely deteriorates the overall dynamic posture of the vehicle.”*

*“More importantly, these control current profiles demonstrate how the inherent damper nonlinearities actively influence the learned policy. As mathematically described in Eq. (5.1), the MFD exhibits strong nonlinear characteristics, particularly near its saturation boundaries represented by the hyperbolic tangent function. Through continuous environmental interactions, the TD3 agent implicitly learns to navigate this physical nonlinearity. Rather than generating aggressive, unbounded current commands that would fall into the unresponsive saturation zones of the damper, the learned policy adapts by continuously adjusting the control current to operate preferentially within the sensitive pseudo-linear regions of the force-velocity profile of MFD, thereby maximizing vibration isolation efficiency.”*

7. The authors should clarify how physical constraints are incorporated into the reinforcement learning framework to ensure the reliability of the obtained control strategy.

**[Response]:** We sincerely thank the reviewer for highlighting the need to clarify how physical constraints are incorporated into our framework. As detailed in the revised manuscript the Actor and Critic networks are not determined arbitrarily. Starting with two layer networks we tested results with different numbers of layers and nodes in increments of 25. For the Critic network two fully connected layers are sufficient with the number of nodes being critical. Typically a two layer network with the same number of nodes per layer is used. Through testing 75 nodes per layer achieved relatively high computational efficiency and after fine tuning we adopted 100 nodes in the first layer and 75 nodes in the second layer to ensure convergence. Since the Actor network involves similar computations it also uses the same first and second layers as the Critic network. Finally to explicitly address the physical constraints and ensure the reliability of the obtained control strategy the output must use an arctan layer to ensure that the output range is reliable. See in lines 512-520.

*“The Actor and Critic networks are not determined arbitrarily: starting with two-layer networks, we tested results with different numbers of layers and nodes (in increments of 25). For the Critic network, two fully connected layers are sufficient, with the number of nodes being critical. Typically, a two-layer network with the same number of nodes per layer is used. Through testing, 75 nodes per layer achieved relatively high computational efficiency, and after fine-tuning, we adopted 100 nodes in the first layer and 75 nodes in the second layer to ensure convergence. Since the Actor network involves similar computations, it also uses the same first and second layers as the Critic network. Finally, the output must use an arctan layer to ensure that the output range is reliable.”*

8. The proposed method is validated mainly under several predefined road excitation conditions, but the generalization capability of the learned control policy is not sufficiently discussed.

**[Response]:** We sincerely thank the reviewer for pointing out the need to further discuss the generalization capability of the learned control policy. As detailed in the revised manuscript the TD3 control method has lower generalization ability than skyhook damping control but higher than fuzzy PID control. For different suspensions of vehicles because road excitations generally remain within the range specified by ISO8608 the variations in suspension output such as suspension velocity and acceleration are limited across different vehicles. Therefore by simply adjusting the reward function a reasonable TD3 controller can be obtained enabling the transfer of the controller from one suspension set to another. At the same time the generalization ability of the TD3 controller results has been preliminarily verified in that when driving speed and road grade change the same training results can produce similar outcomes and the output damping force remains consistently within the normal range without showing prolonged periods of excessively high or low damping. Thus it can be considered that using the TD3 algorithm to design a controller in a suspension model does not lead to significant overfitting under reasonable parameters. Through multiple training sessions it is considered that under the same speed condition approximately 30000 seconds of training is sufficient to obtain relatively reasonable controller results and for suspension variations occurring over time due to usage retraining can be performed to obtain more reasonable controller parameters thereby ensuring the reliability of passenger cars throughout their entire operational cycle. See in lines 280-296.

*“For suspension control, the TD3 control method has lower generalization ability than skyhook damping control but higher than fuzzy PID control. For different suspensions of vehicles, because road excitations generally remain within the range specified by ISO8608, the variations in suspension output, such as suspension velocity and acceleration, are limited across different vehicles. Therefore, by simply adjusting the reward function, a reasonable TD3 controller can be obtained, enabling the transfer of the controller from one suspension set to another. At the same time, the generalization ability of the TD3 controller results has been preliminarily verified: when driving speed and road grade change, the same training results can produce similar outcomes, and the output damping force remains consistently within the normal range without showing prolonged periods of excessively high or low damping. Thus, it can be considered that using the TD3 algorithm to design a controller in a suspension model does not lead to significant overfitting under reasonable parameters. Through multiple training sessions, it is considered that under the same speed condition, approximately 30,000 seconds of training is sufficient to obtain relatively reasonable controller results. For suspension variations occurring over time due to usage, retraining can be performed to obtain more reasonable controller parameters, thereby ensuring the reliability of passenger cars throughout their entire operational cycle.”*

9. Since reinforcement learning controllers may strongly depend on training environments, the authors should analyze the robustness of the proposed method when vehicle parameters, road excitation characteristics, or operating conditions deviate from those used during training.

**[Response]:** We sincerely appreciate the reviewer for highlighting the importance of analyzing robustness against varying environments. As detailed in the revised manuscript the TD3 control method has lower generalization ability than skyhook damping control but higher than fuzzy PID control. For different suspensions of vehicles because road excitations generally remain within the range specified by ISO8608 the variations in suspension output such as suspension velocity and acceleration are limited across different vehicles. Therefore by simply adjusting the reward function a reasonable TD3 controller can be obtained enabling the transfer of the controller from one suspension set to another. At the same time the generalization ability of the TD3 controller results has been preliminarily verified in that when driving speed and road grade change the same training results can produce similar outcomes and the output damping force remains consistently within the normal range without showing prolonged periods of excessively high or low damping. Thus it can be considered that using the TD3 algorithm to design a controller in a suspension model does not lead to significant overfitting under reasonable parameters. Through multiple training sessions it is considered that under the same speed condition approximately 30000 seconds of training is sufficient to obtain relatively reasonable controller results and for suspension variations occurring over time due to usage retraining can be performed to obtain more reasonable controller parameters thereby ensuring the reliability of passenger cars throughout their entire operational cycle. See in lines 280-296.

“For suspension control, the TD3 control method has lower generalization ability than skyhook damping control but higher than fuzzy PID control. For different suspensions of vehicles, because road excitations generally remain within the range specified by ISO8608, the variations in suspension output, such as suspension velocity and acceleration, are limited across different vehicles. Therefore, by simply adjusting the reward function, a reasonable TD3 controller can be obtained, enabling the transfer of the controller from one suspension set to another. At the same time, the generalization ability of the TD3 controller results has been preliminarily verified: when driving speed and road grade change, the same training results can produce similar outcomes, and the output damping force remains consistently within the normal range without showing prolonged periods of excessively high or low damping. Thus, it can be considered that using the TD3 algorithm to design a controller in a suspension model does not lead to significant overfitting under reasonable parameters. Through multiple training sessions, it is considered that under the same speed condition, approximately 30,000 seconds of training is sufficient to obtain relatively reasonable controller results. For suspension variations occurring over time due to usage, retraining can be performed to obtain more reasonable controller parameters, thereby ensuring the reliability of passenger cars throughout their entire operational cycle.”

10. The background should highlight the rapid development of the active suspension technology, thereby enhancing the research value of this paper. Some recent work, such as "Integrating Torque Vectoring and Active Suspension Systems Using a Game Theory-Based Control Framework, IEEE Transactions on Automation Science and Engineering, vol. 23, pp. 703-716, 2026", and "An Integrated Control Framework for Torque Vectoring and Active Suspension System. Chin. J. Mech. Eng. 37, 10 (2024)." can be referred.

**[Response]:** We sincerely thank the reviewer for the constructive suggestion to highlight the rapid development of active suspension technology and for recommending the highly relevant recent works. In response to this valuable feedback, we have expanded the introduction section to emphasize the fast-paced evolution of active and semi-active suspension technologies, which further solidifies the research motivation and value of our work. Furthermore, we have carefully studied the recommended literature concerning the integrated control frameworks for torque vectoring and active suspension systems. These important studies have been incorporated into the revised manuscript as key background references to better illustrate the necessity of comprehensive chassis dynamics modeling in a full-vehicle context. We believe these revisions have significantly enriched the background discussion and strengthened the overall manuscript. See in lines 75-83 and 128-136.

*“Furthermore recent studies emphasize the critical importance of integrated chassis control in vehicle models to meet modern performance demands. For example to simultaneously enhance longitudinal dynamics and vertical ride comfort advanced frameworks based on multi-agent systems have been proposed to integrate active suspension systems with torque vectoring [45]. Similarly model predictive controllers have been developed for such integrated systems to optimize energy saving and safety under linear models [46]. Driven by the necessity of such comprehensive dynamics modeling and multi-axis coordination this paper employs a seven-degree-of-freedom vehicle suspension model, which is a full vehicle model, to thoroughly investigate suspension control effects.”*

*“In recent years, driven by the rapid trends of vehicle electrification [33] and intelligent autonomous driving [34], active and semi-active suspension technologies have experienced unprecedented development to meet increasingly stringent demands for ride comfort and integrated chassis control. While the performance of traditional controllers like Skyhook is strongly influenced by hardware constraints, such as the response time of magnetorheological valves [35], DRL control algorithms using the Actor-Critic structure (such as DDPG) can often achieve more robust and adaptable control. For instance, recent studies have successfully deployed Soft Actor-Critic (SAC) models in real vehicles, enabling the suspension system to dynamically adapt to varying real-road disturbance profiles [36].”*
