# Supplementary material for: A twin delayed deep deterministic-based control method for a full vehicle semi-active suspension system
Source: PLoS One. 2026 Sep 25;21(9):e0359102. doi: 10.1371/journal.pone.0359102 (PMC13614603; doi:10.1371/journal.pone.0359102)
Supplement: Peer review history

# Peer review: Decision Letter 0

**DOI:** 10.1371/journal.pone.0359102.r001

**Authors:** Jinhao Liang

9 Jul 2026

Dear Dr. Zhang,

Thank you for submitting your manuscript to PLOS ONE. After careful consideration, we feel that it has merit but does not fully meet PLOS ONE’s publication criteria as it currently stands. Therefore, we invite you to submit a revised version of the manuscript that addresses the points raised during the review process.

Please submit your revised manuscript by Sep 07 2026 11:59PM. If you will need more time than this to complete your revisions, please reply to this message or contact the journal office at plosone@plos.org. When you're ready to submit your revision, log on to https://www.editorialmanager.com/pone/ and select the 'Submissions Needing Revision' folder to locate your manuscript file.

- A letter that responds to each point raised by the academic editor and reviewer(s). You should upload this letter as a separate file labeled 'Response to Reviewers'.
- A marked-up copy of your manuscript that highlights changes made to the original version. You should upload this as a separate file labeled 'Revised Manuscript with Track Changes'.
- An unmarked version of your revised paper without tracked changes. You should upload this as a separate file labeled 'Manuscript'.

If you would like to make changes to your financial disclosure, please include your updated statement in your cover letter. Guidelines for resubmitting your figure files are available below the reviewer comments at the end of this letter.

If applicable, we recommend that you deposit your laboratory protocols in protocols.io to enhance the reproducibility of your results. Protocols.io assigns your protocol its own identifier (DOI) so that it can be cited independently in the future. For instructions see: https://journals.plos.org/plosone/s/submission-guidelines#loc-laboratory-protocols. Additionally, PLOS ONE offers an option for publishing peer-reviewed Lab Protocol articles, which describe protocols hosted on protocols.io. Read more information on sharing protocols at https://plos.org/protocols?utm_medium=editorial-email&utm_source=authorletters&utm_campaign=protocols.

As the corresponding author, your ORCID iD is verified in the submission system and will appear in the published article. PLOS supports the use of ORCID, and we encourage all coauthors to register for an ORCID iD and use it as well. Please encourage your coauthors to verify their ORCID iD within the submission system before final acceptance, as unverified ORCID iDs will not appear in the published article. *Only*  the individual author can complete the verification step; PLOS staff *cannot*  verify ORCID iDs on behalf of authors.

We look forward to receiving your revised manuscript.

Kind regards,

Jinhao Liang

Academic Editor

PLOS One

Journal Requirements:

When submitting your revision, we need you to address these additional requirements.

1. Please ensure that your manuscript meets PLOS ONE's style requirements, including those for file naming. The PLOS ONE style templates can be found at

https://journals.plos.org/plosone/s/file?id=wjVg/PLOSOne_formatting_sample_main_body.pdf and

https://journals.plos.org/plosone/s/file?id=ba62/PLOSOne_formatting_sample_title_authors_affiliations.pdf

2. Please note that PLOS One has specific guidelines on code sharing for submissions in which author-generated code underpins the findings in the manuscript. In these cases, we expect all author-generated code to be made available without restrictions upon publication of the work. Please review our guidelines at https://journals.plos.org/plosone/s/materials-and-software-sharing#loc-sharing-code and ensure that your code is shared in a way that follows best practice and facilitates reproducibility and reuse.

3. Please amend the manuscript submission data (via Edit Submission) to include author Yongjun Wan, Xiaoming Wang, Lei Yang, Juye Wang and Xiangcheng Zhang.

4. Thank you for stating the following in the Competing Interests section:

“The authors have declared that no competing interests exist.”

We note that one or more of the authors are employed by a commercial company: “China Construction Fifth Bureau (Henan) Construction Co., Ltd.”

1. Please provide an amended Funding Statement declaring this commercial affiliation, as well as a statement regarding the Role of Funders in your study. If the funding organization did not play a role in the study design, data collection and analysis, decision to publish, or preparation of the manuscript and only provided financial support in the form of authors' salaries and/or research materials, please review your statements relating to the author contributions, and ensure you have specifically and accurately indicated the role(s) that these authors had in your study. You can update author roles in the Author Contributions section of the online submission form.

Please also include the following statement within your amended Funding Statement.

“The funder provided support in the form of salaries for authors [insert relevant initials], but did not have any additional role in the study design, data collection and analysis, decision to publish, or preparation of the manuscript. The specific roles of these authors are articulated in the ‘author contributions’ section.”

If your commercial affiliation did play a role in your study, please state and explain this role within your updated Funding Statement.

2. Please also provide an updated Competing Interests Statement declaring this commercial affiliation along with any other relevant declarations relating to employment, consultancy, patents, products in development, or marketed products, etc.

Within your Competing Interests Statement, please confirm that this commercial affiliation does not alter your adherence to all PLOS ONE policies on sharing data and materials by including the following statement: "This does not alter our adherence to  PLOS ONE policies on sharing data and materials.” (as detailed online in our guide for authors http://journals.plos.org/plosone/s/competing-interests) . If this adherence statement is not accurate and  there are restrictions on sharing of data and/or materials, please state these. Please note that we cannot proceed with consideration of your article until this information has been declared.

Please include both an updated Funding Statement and Competing Interests Statement in your cover letter. We will change the online submission form on your behalf.

5. Please include a new copy of Table 3 in your manuscript; the current table is difficult to read. Please follow the link for more information: https://journals.plos.org/plosone/s/tables

6. If the reviewer comments include a recommendation to cite specific previously published works, please review and evaluate these publications to determine whether they are relevant and should be cited. There is no requirement to cite these works unless the editor has indicated otherwise.

[Note: HTML markup is below. Please do not edit.]

Reviewers' comments:

Reviewer's Responses to Questions

**Comments to the Author**

1. Is the manuscript technically sound, and do the data support the conclusions?

Reviewer #1: Yes

Reviewer #2: Yes

**********

2. Has the statistical analysis been performed appropriately and rigorously?

Reviewer #1: Yes

Reviewer #2: Yes

**********

3. Have the authors made all data underlying the findings in their manuscript fully available?

The PLOS Data policy

Reviewer #1: Yes

Reviewer #2: Yes

**********

4. Is the manuscript presented in an intelligible fashion and written in standard English?

Reviewer #1: Yes

Reviewer #2: Yes

**********

Reviewer #1: Abstract

The abbreviation definitions can be moved to the body text.

Mention the quantitative improvements more clearly and compare with which recent sophisticated methods, not just the classical Skyhook.

Introduction

Avoid group referencing.

The literature review needs to be enriched by some recent research works. As the subject is very hot in recent years, it can enrich the literature review. Some suggestive references for enriching the literature review:

Similar data-driven and advanced control approaches for magnetorheological systems are discussed in (DOI: 10.1109/TCYB.2025.3596063).

For efficient deep reinforcement learning frameworks in vehicle control, similar approaches could be strengthened by referring to (DOI: 10.1109/TVT.2025.3629701) and (DOI: 10.1016/j.knosys.2025.114613).

Add a paragraph to introduce the paper organisation at the end of the Introduction.

7-DOF vehicle semi-active suspension model

"Table 4. Parameters of vehicle suspension model." How do you determine the uncertainties? Lack of physical discussion about the parameters. How can the control design be generalized for the other cases?

"The hysteresis characteristics of the MFD were neglected..." Why? This is a major simplification. More discussions are needed about the MFD model and validation method.

Road surface roughness model & 4. TD3 semi-active suspension controller

"The reward function R(s)..." What's the meaning of the weights? How do you determine them? Your design process seems very simple, like a course homework!

How do you evaluate the ride comfort with respect to the ISO 2631 standard criterion and frequency-based calculation?

"Table 5 & 6. Specification of critic/actor network." Why these specific numbers of neurons and layers? Lack of discussion about the hyperparameter tuning and ablation study.

How do you consider the actuator delay and energy effort of the proposed semi-active suspension?

Simulation results

The comparison is only done with passive suspension and the classical linear Skyhook control method. This does not seem like an attractive contribution for a research paper at 2026! The comparison should be done with a more sophisticated method. For integrated vehicle stability and advanced control comparisons, you may refer to (DOI: 10.1109/TFUZZ.2026.3695547).

The results only show time-domain responses. What about the frequency-domain analysis? The vibration performance should be evaluated by the frequency-based approach.

"Fig 5, 6, 7." Please upgrade the page arrangements. Modify the page design to avoid a figure break during the page and column changes!

How do you evaluate the active suspension energy consumption and optimize it?

How much is the possibility of implementing your proposed method on a cost-effective microcontroller?

Reviewer #2: This manuscript proposes a TD3-based deep reinforcement learning control method for a full-vehicle semi-active suspension system. The topic is relevant to intelligent vehicle suspension control, and the proposed framework provides a meaningful attempt to apply DRL methods to a 7-DOF semi-active suspension model. However, the current manuscript still lacks sufficient methodological depth and theoretical analysis to clearly demonstrate the advantages and novelty of the proposed approach. Several key issues related to the control design, reinforcement learning formulation, and practical applicability need to be further clarified and improved before the work can be considered for publication.

1. The main contribution of the proposed TD3-based controller is not sufficiently distinguished from existing DRL-based suspension control methods. The manuscript mainly applies the standard TD3 algorithm with modifications of network structure, training parameters, and reward function, but the specific methodological innovations and their contributions to improving suspension control performance are not clearly explained. The authors should further clarify what fundamental improvements are introduced compared with conventional DRL control frameworks.

2. The design of the state space, action space, and reward function is critical for reinforcement learning-based vehicle control, but the current explanation is insufficient. The reward function mainly considers velocity and acceleration-related variables, while other important suspension performance objectives, such as suspension working space, tire-road contact condition, and actuator constraints, are not explicitly considered. The authors should provide a more comprehensive discussion on how the reward function reflects the multi-objective requirements of semi-active suspension systems.

3. The relationship between the TD3 controller and the physical characteristics of the semi-active suspension system requires further analysis. The proposed method relies on data-driven learning, but the influence of vehicle dynamics characteristics, damper nonlinearities, and actuator limitations on the learned control policy is not sufficiently discussed. The authors should clarify how physical constraints are incorporated into the reinforcement learning framework to ensure the reliability of the obtained control strategy.

4. The proposed method is validated mainly under several predefined road excitation conditions, but the generalization capability of the learned control policy is not sufficiently discussed. Since reinforcement learning controllers may strongly depend on training environments, the authors should analyze the robustness of the proposed method when vehicle parameters, road excitation characteristics, or operating conditions deviate from those used during training.

5. The background should highlight the rapid development of the active suspension technology, thereby enhancing the research value of this paper. Some recent work, such as “Integrating Torque Vectoring and Active Suspension Systems Using a Game Theory-Based Control Framework, IEEE Transactions on Automation Science and Engineering, vol. 23, pp. 703-716, 2026”, and “An Integrated Control Framework for Torque Vectoring and Active Suspension System. Chin. J. Mech. Eng. 37, 10 (2024).” can be referred.

**********

what does this mean?). If published, this will include your full peer review and any attached files.

If you choose “no”, your identity will remain anonymous but your review may still be made public.

**Do you want your identity to be public for this peer review?**  For information about this choice, including consent withdrawal, please see our Privacy Policy

Reviewer #1: **Yes:** Masoud Masih-Tehrani

Reviewer #2: No

**********

[NOTE: If reviewer comments were submitted as an attachment file, they will be attached to this email and accessible via the submission site. Please log into your account, locate the manuscript record, and check for the action link "View Attachments". If this link does not appear, there are no attachment files.]

To ensure your figures meet our technical requirements, please review our figure guidelines: https://journals.plos.org/plosone/s/figures

You may also use PLOS’s free figure tool, NAAS, to help you prepare publication quality figures: https://journals.plos.org/plosone/s/figures#loc-tools-for-figure-preparation.

NAAS will assess whether your figures meet our technical requirements by comparing each figure against our figure specifications.

# Author comment: Author response to Decision Letter 1

**DOI:** 10.1371/journal.pone.0359102.r002

1 Sep 2026

We sincerely thank you for your valuable comments and suggestions. A detailed point-by-point response to all reviewer and editor comments, including all tables, algorithm descriptions, and comparative data, has been uploaded as a separate file (Response to Reviewers.docx). All revisions have been marked in red in the revised manuscript. We hope this response fully addresses your concerns.

Attachment Submitted filename: Response to Reviewers.docx

# Peer review: Decision Letter 1

**DOI:** 10.1371/journal.pone.0359102.r003

**Authors:** Jinhao Liang

9 Sep 2026

A Twin Delayed Deep Deterministic-based control method for a full vehicle semi-active suspension system

PONE-D-26-32255R1

Dear Dr. Zhang,

We’re pleased to inform you that your manuscript has been judged scientifically suitable for publication and will be formally accepted for publication once it meets all outstanding technical requirements.

Within one week, you’ll receive an e-mail detailing the required amendments. When these have been addressed, you’ll receive a formal acceptance letter and your manuscript will be scheduled for publication.

An invoice will be generated when your article is formally accepted. Please note, if your institution has a publishing partnership with PLOS and your article meets the relevant criteria, all or part of your publication costs will be covered. Please make sure your user information is up-to-date by logging into Editorial Manager at Editorial Manager® and clicking the ‘Update My Information' link at the top of the page. For questions related to billing, please contact billing support.

If your institution or institutions have a press office, please notify them about your upcoming paper to help maximize its impact. If they’ll be preparing press materials, please inform our press team as soon as possible -- no later than 48 hours after receiving the formal acceptance. Your manuscript will remain under strict press embargo until 2 pm Eastern Time on the date of publication. For more information, please contact onepress@plos.org.

Kind regards,

Jinhao Liang

Academic Editor

PLOS One

Additional Editor Comments (optional):

Reviewers' comments:

Reviewer's Responses to Questions

**Comments to the Author**

Reviewer #1: All comments have been addressed

Reviewer #2: All comments have been addressed

**********

2. Is the manuscript technically sound, and do the data support the conclusions?

Reviewer #1: Yes

Reviewer #2: Yes

**********

3. Has the statistical analysis been performed appropriately and rigorously?

Reviewer #1: Yes

Reviewer #2: Yes

**********

4. Have the authors made all data underlying the findings in their manuscript fully available?

The PLOS Data policy

Reviewer #1: Yes

Reviewer #2: Yes

**********

5. Is the manuscript presented in an intelligible fashion and written in standard English?

Reviewer #1: Yes

Reviewer #2: Yes

**********

Reviewer #1: Thanks for the deep revision.........................................................................................

Reviewer #2: The revised manuscript has addressed the reviewers’ all concerns adequately, and I recommend acceptance.

**********

what does this mean?). If published, this will include your full peer review and any attached files.

If you choose “no”, your identity will remain anonymous but your review may still be made public.

**Do you want your identity to be public for this peer review?**  For information about this choice, including consent withdrawal, please see our Privacy Policy

Reviewer #1: **Yes:** Masoud Masih-Tehrani

Reviewer #2: No

**********

# Editor report: Acceptance letter

**DOI:** 10.1371/journal.pone.0359102.r004

**Authors:** Jinhao Liang

PONE-D-26-32255R1

PLOS One

Dear Dr. Zhang,

I'm pleased to inform you that your manuscript has been deemed suitable for publication in PLOS One. Congratulations! Your manuscript is now being handed over to our production team.

At this stage, our production department will prepare your paper for publication. This includes ensuring the following:

* All references, tables, and figures are properly cited

* All relevant supporting information is included in the manuscript submission,

* There are no issues that prevent the paper from being properly typeset

You will receive further instructions from the production team, including instructions on how to review your proof when it is ready. Please keep in mind that we are working through a large volume of accepted articles, so please give us a few days to review your paper and let you know the next and final steps.

Lastly, if your institution or institutions have a press office, please let them know about your upcoming paper now to help maximize its impact. If they'll be preparing press materials, please inform our press team within the next 48 hours. Your manuscript will remain under strict press embargo until 2 pm Eastern Time on the date of publication. For more information, please contact onepress@plos.org.

You will receive an invoice from PLOS for your publication fee after your manuscript has reached the completed accept phase. If you receive an email requesting payment before acceptance or for any other service, this may be a phishing scheme. Learn how to identify phishing emails and protect your accounts at https://explore.plos.org/phishing.

If we can help with anything else, please email us at customercare@plos.org.

Thank you for submitting your work to PLOS One and supporting open access.

Kind regards,

PLOS One Editorial Office Staff

on behalf of

Dr. Jinhao Liang

Academic Editor

PLOS One
